# Penpulimab, an anti-PD-1 antibody, for heavily pretreated metastatic nasopharyngeal carcinoma: a single-arm phase II study

**DOI:** 10.1038/s41392-024-01865-6

**Published:** 2024-06-19

**Authors:** Xiaozhong Chen, Wei Wang, Qingfeng Zou, Xiaodong Zhu, Qin Lin, Yi Jiang, Yan Sun, Liangfang Shen, Lin Wang, Guorong Zou, Xiaoyan Lin, Shaojun Lin, Minying Li, Ying Wang, Ruilian Xu, Rui Ao, Rensheng Wang, Haifeng Lin, Shuang Huang, Tingting Xu, Wenting Li, Mengying Xia, Yu Xia, Zhongmin Wang, Baiyong Li, Jingao Li, Chaosu Hu

**Affiliations:** 1 Department of Radiation Oncology, Cancer Hospital of the University of Chinese, Academy of Sciences (Zhejiang Cancer Hospital), Hangzhou, China; 2 Gastroenterology and Urology Department II, Hunan Cancer Hospital/the Affiliated Cancer Hospital of Xiangya School of Medicine, Central South University, Changsha, China; 3 Clinical Research Center for Gastrointestinal Cancer in Hunan Province, Changsha, China; 4 Department of Medical Oncology, Affiliated Cancer Hospital & Institute of Guangzhou Medical University, Guangzhou, China; 5 Department of Radiation Oncology, Guangxi Medical University Affiliated Tumor Hospital & Key Laboratory of Early Prevention and Treatment for Regional High Frequency Tumor (Guangxi Medical University), Ministry of Education & Guangxi Key Laboratory of Early Prevention and Treatment for Regional High Frequency Tumor, Guangxi Medical University, Nanning, China; 6 Department of Radiation Oncology, Cancer Center, The First Affiliated Hospital of Xiamen University, Xiamen, China; 7 Department of Medical Oncology, Cancer Hospital of Shantou University Medical College, Shantou, China; 8 Department of Radiation Oncology, Peking University Cancer Hospital & Institute, Beijing, China; 9 Department of Oncology, Xiangya Hospital Central South University, Changsha, China; 10 Department of Medical Oncology, Hainan General Hospital, Hainan Affiliated Hospital of Hainan Medical University, Haikou, China; 11 Department of Oncology, The Affiliated Panyu Central Hospital of Guangzhou Medical University, Guangzhou, China; 12 Department of Medical Oncology, Fujian Medical University Union Hospital, Fuzhou, China; 13 Department of Radiation Oncology, Clinical Oncology School of Fujian Medical University, Fujian Cancer Hospital, Fuzhou, China; 14 Department of Radiation Therapy for Thoracic Tumors, Zhongshan City People’s Hospital, Zhongshan, China; 15 Tumor Radiotherapy Center, Chongqing University Cancer Hospital, Chongqing Cancer Hospital, Chongqing Cancer Institute, Chongqing, China; 16 Department of Oncology, Shenzhen People’s Hospital (The Second Clinical Medical College, Jinan University; The First Affiliated Hospital, Southern University of Science and Technology), Shenzhen, China; 17 Oncology Center, Sichuan Academy of Medical Sciences & Sichuan Provincial People’s Hospital, School of Medicine UESTC, Chengdu, China; 18 Department of Radiation Oncology, The First Affiliated Hospital of Guangxi Medical University, Nanning, China; 19 Department of Medical Oncology, The Second Affiliated Hospital of Hainan Medica University, Haikou, China; 20 Department of Radiation Oncology, Fudan University, Shanghai Cancer Center, Shanghai, China; 21 Department of Oncology, Shanghai Medical College, Fudan University, Shanghai, China; 22 Shanghai Key Laboratory of Radiation Oncology, Shanghai, China; 23 Akeso Biopharma Inc., Zhongshan, China; 24 Department of Radiation Oncology, Jiangxi Cancer Hospital, Nanchang, China; 25 NHC Key Laboratory of Personalized Diagnosis and Treatment of Nasopharyngeal Carcinoma (Jiangxi Cancer Hospital, Nanchang Medical College), Nanchang, China

**Keywords:** Oncology, Cancer

## Abstract

Penpulimab is an anti-programmed cell death-1 (PD-1) IgG1 antibody with no Fc gamma receptor (FcγR) binding activity, and thus theoretically reduced immune-related adverse events (irAEs) while maintaining efficacy. This single-arm, phase II trial conducted across 20 tertiary care centers in China enrolled adult patients with metastatic nasopharyngeal carcinoma (NPC) who had failed two or more lines of previous systemic chemotherapy. Patients received 200-mg penpulimab intravenously every 2 weeks (4 weeks per cycle) until disease progression or intolerable toxicities. The primary endpoint was objective response rate (ORR) per RECIST (version 1.1), as assessed by an independent radiological review committee. The secondary endpoints included progression-free survival (PFS) and overall survival (OS). One hundred thirty patients were enrolled and 125 were efficacy evaluable. At the data cutoff date (September 28, 2022), 1 patient achieved complete response and 34 patients attained partial response. The ORR was 28.0% (95% CI 20.3–36.7%). The response was durable, with 66.8% still in response at 9 months. Thirty-three patients (26.4%) were still on treatment. The median PFS and OS were 3.6 months (95% CI = 1.9–7.3 months) and 22.8 months (95% CI = 17.1 months to not reached), respectively. Ten (7.6%) patients experienced grade 3 or higher irAEs. Penpulimab has promising anti-tumor activities and acceptable toxicities in heavily pretreated metastatic NPC patients, supporting further clinical development as third-line treatment of metastatic NPC.

## Introduction

Approximately 15% of patients with nasopharyngeal carcinoma (NPC) have metastatic (R/M) disease on initial diagnosis, and about 30% of stage III-IVa NPC patients eventually experience distant recurrence.^1,2^ The standard first-line treatment is systemic chemotherapy with gemcitabine plus cisplatin and immune checkpoint inhibitors (ICIs); single-agent chemotherapy is often used as second-line treatment. No preferred third-line treatment regimen is available for R/M NPC patients.

ICIs have been investigated as second- and later-line therapies for R/M NPC and have exhibited promising activities, highlighting the therapeutic potential of anti-programmed cell death-1 (PD-1) monoclonal antibodies for R/M NPC.^3–12^ However, these ICIs attained an objective response rate (ORR) of 20.5% (toripalimab and nivolumab) and 25.9% (pembrolizumab) as 2^nd^ or later line for R/M NPC, suggesting that most R/M NPC patients do not exhibit demonstrable clinical response.^3,10,12^ Besides, toripalimab, tislelizumab, and camrelizumab are all humanized, IgG4 monoclonal antibodies with binding specificity for PD-1; these IgG4 antibodies are similar to wildtype human IgG4 and possesses effector-binding capabilities, which negatively impacts on anti-PD-1 antibody-mediated anti-cancer activities.^13–17^ Anti-PD-1 IgG4 antibodies could also engage FcγRI^+^ macrophages, induce antibody-dependent cellular phagocytosis, damage PD-1^+^ T cells, and stimulate the release of inflammatory cytokines, which are responsible for toxicities associated with PD-1/PD-L1 blockade.^17^ IgG4 Fc binding to Fc gamma receptors (FcγRs) also induces antibody-dependent cell-mediated cytotoxicity (ADCC) and releases inflammatory cytokines, which may cause immune-related adverse events (irAEs).^18^

To minimize the toxicities and improve the clinical efficacy of PD-1/PD-L1 blockade, we developed penpulimab (AK105), a human IgG1 monoclonal antibody against human PD-1. Penpulimab has undergone fragment crystallizable (Fc) mutation and thus has eliminated Fc receptor and complement-mediated effector function.^19^ As a result, antibody-dependent cell-mediated cytotoxicity and complement-dependent cytotoxicity are avoided, theoretically lessening the occurrences of irAEs.^20^ A pooled analysis of 465 patients from 6 trials showed 3.5% grade 3 or higher irAEs. Penpulimab has also been explored as monotherapy for advanced upper gastrointestinal cancers and refractory or relapsed classical Hodgkin lymphoma.^21–23^ Given the encouraging data on ICIs for R/M NPC and the overall benign safety profile of penpulimab, we hypothesized that penpulimab could offer advanced NPC patients an effective and safe treatment option.

Currently, there is no standard third-line treatment for patients with metastatic NPC. Also, no anti-PD-1 IgG1 antibody with complete elimination of FcγR binding activity has been examined for this subset of patients. In this trial, we evaluated the efficacy and safety of penpulimab in metastatic NPC patients who failed first-line platinum-based chemotherapy and second-line chemotherapy. Prespecified subgroup analysis was conducted to identify the subpopulations of patients who could benefit from the treatment.

## Results

### Patients

Between March 6, 2019, and September 14, 2020, 171 NPC patients were screened and 130 (median age 49.5 years and men 76.0%) were enrolled and constituted the intention-to-treat population. Four patients with no measurable lesions per the Independent Radiological Review Committee (IRRC) and one patient who had received only one prior line of chemotherapy were excluded from efficacy analysis. The FAS per IRRC included 125 patients. In addition, one patient who had no baseline measurable lesion according to investigators, and one patient who did not receive at least 2 prior lines of chemotherapy were excluded. The FAS per investigators included 128 patients. Forty-six (36.8%) patients in the FAS per IRRC were PD-L1-positive. Serum EBV copy number at baseline was ≥500 IU/mL in 103 (82.4%) patients. Seventy-seven (61.6%) patients had lung metastasis, 62 (49.6%) had bone metastasis, and 60 (48.0%) had liver metastasis. For prior targeted therapies, 36 patients (27.7%) received prior nimotuzumab, 4 (3.1%) received endostatin and 2 (1.5%) received cetuximab. Forty-six (36.8%) patients had received at least three prior lines of systemic therapy (Table 1).

**Table 1:** Patient demographics and baseline characteristics-ITT

Variables	N = 125
Age, years, median (range)	49.6 (20.8–65.7)
≤65	123 (98.4)
>65	2 (1.6)
Sex, n (%)	
Male	95 (76.0)
Female	30 (24.0)
ECOG PS, n (%)	
0	39 (31.2)
1	86 (68.8)
Histological types, n (%)	
Nonkeratinizing differentiated NPC	37 (29.6)
Nonkeratinizing undifferentiated NPC	84 (67.2)
Missing	4 (3.2)
PD-L1 expression, n (%)	
Positive (TPS ≥ 50%)	46 (36.8)
Negative (TPS < 50%)	76 (60.8)
Not available	3 (2.4)
Metastatic sites at baseline, n (%)	
Lung	77 (61.6)
Bone	62 (49.6)
Liver	60 (48.0)
Lymph Node	44 (35.2)
Others	15 (12.0)
Number of metastatic sites, n (%)	
<3	84 (67.2)
≥3	41 (32.8)
Time from initial diagnosis to 1^st^ penpulimab dose, n (%)	
<12 months	9 (7.2)
≥12 months	116 (92.8)
Serum EBV copy number at baseline, n (%)	
<500 IU/mL	22 (17.6)
≥500 IU/mL	103 (82.4)
Baseline LDH, n (%)	
LDH < ULN	64 (51.2)
LDH ≥ ULN	61 (48.8)
Prior radiotherapy, n (%)	115 (92.0)
Prior primary site radiotherapy, n (%)	106 (84.8)
Prior anti-cancer surgery, n (%)	20 (16.0)
Number of prior lines of therapy, n (%)	
2	79 (63.2)
≥3	46 (36.8)

The normal reference value for EBV DNA in the testing laboratories was 500 IU/mL and any samples ≥500 IU/mL were considered positive for EBV DNA

*ECOG PS* Eastern Cooperative Oncology Group performance status score, *LDH* lactate dehydrogenase, *NPC* nasopharyngeal carcinoma, *PD-L1* programmed death-ligand 1, *TPS* tumor proportion score, *ULN* upper limit normal

**Fig. 1:**
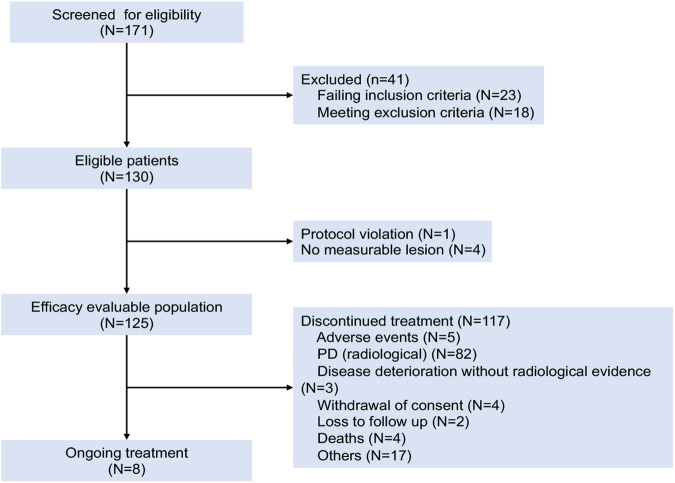
The study flowchart. PD progressive disease

The data cutoff date was September 28, 2022. The median follow-up was 29.6 months. The median treatment duration was 4.1 months (range, 0.03–40.9 months). Eight (6.4%) patients were still receiving treatment. One hundred seventeen (93.6%) patients discontinued treatment because of radiologic disease progression (n = 82), clinical deterioration without radiologic evidence (n = 3), adverse events (n = 5), withdrawal of consent (n = 4), loss to follow-up (n = 2), death (n = 4), and other causes (n = 17) (Fig. 1). Sixty-five out of 93 patients (69.9%) who had disease progression continued treatment with penpulimab and 53.9% went on to receive additional anticancer treatment.

### Efficacy measures

**Fig. 2:**
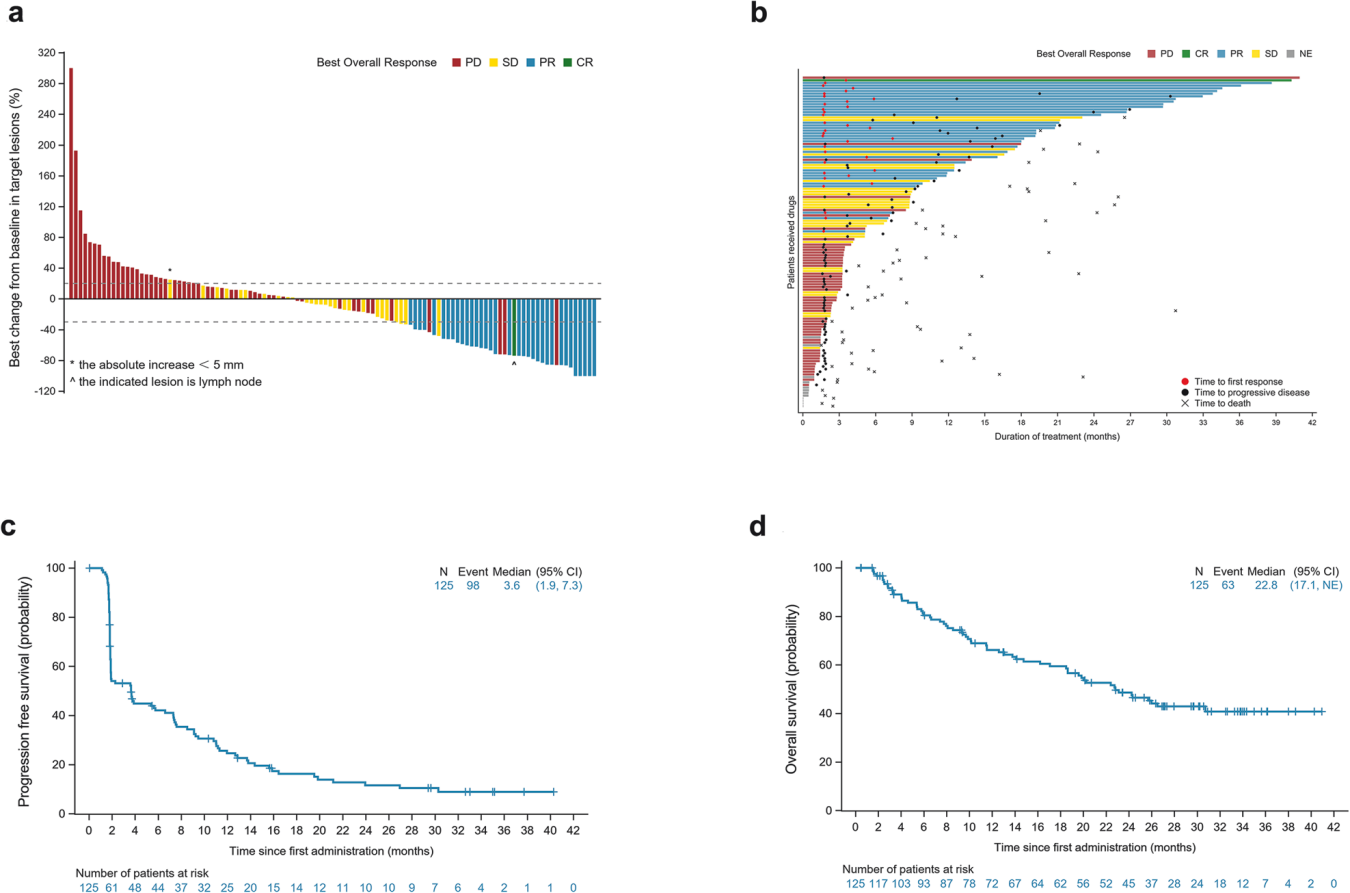
Treatment response and survival outcomes. **a** Waterfall plot of the best percentage changes for the sum of target lesion diameters for patients received at least one time of radiographic evaluation. *This patient had a >20% increase in the sum of diameter, but with absolute increase <5 mm, per the IRRC and, therefore, stable disease (SD) was documented instead of progressive disease (PD). ^The indicated lesion in this patient is lymph node. **b** Swimmer plots of time to tumor response (months) of individual patients with metastatic nasopharyngeal carcinoma as assessed by the independent radiological review committee (IRCC) according to the Response Evaluation Criteria in Solid Tumors (RECIST), version 1.1. Each swim lane represents one patient in the full-analysis set (FAS) per IRRC. Patient responses are color coded. CR complete response, NE not evaluable, PD progressive disease, PR partial response, SD stable disease. Kaplan–Meier-estimated progression-free survival (PFS) curves (**c**) and overall survival (OS) curves (**d**) of NPC patients in the FAS per IRRC

In the FAS per IRRC, 1 (0.8%, 95% CI = 0.0–4.4%) patient achieved complete response (CR) and 34 (27.2%, 95% CI = 19.6–35.9%) achieved partial response (PR) (Fig. 2a). The ORR was 28.0% (95% CI = 20.3–36.7%). The median time to response was 1.8 months (95% CI = 1.6–7.4 months), and the median duration of response was 14.8 months (95% CI = 8.9–25.3 months). At 9 months, 66.8% (95% CI = 48.1–80.0%) of the patients were in response (Fig. 2b). At the data cutoff, 10.4% patients were still in remission. Additionally, 27 patients had SD and 51 developed PD. The disease control rate (DCR) was 49.6% (95% CI = 40.5–58.7%).

Eighty-six PFS events occurred, and the median PFS was 3.6 months (95% CI = 1.9–7.3 months) (Fig. 2c). The 6-month and 12-month PFS rate was 42.1% (95% CI = 32.9–51.0%), and 24.7% (95% CI = 17.0–33.1%), respectively. At the data cutoff, 48 (36.9%) deaths were reported in the FAS. The median OS was 22.8 months (95% CI = 17.1 months to not reached) (Fig. 2d). The 12 and 24-month OS rate was 66.1% (95% CI = 56.7–74.0%) and 48.6% (95% CI = 38.9–57.7%), respectively.

### Safety

All 130 patients were included in the safety analysis. TRAEs occurred in 71.5% of the patients. The three most frequent any grade TRAEs were hypothyroidism (30.0%), anemia (15.4%), and aspartate aminotransferase (AST) increased (14.6%). Fourteen (10.8%) patients experienced grade ≥ 3 TRAEs (Table 2). The rate of irAEs was 48.5% (63/130). The most frequent irAEs were hypothyroidism (20.8%), blood thyroid stimulating hormone increased (12.3%), rash (6.2%), and AST increased (6.2%). Ten (10/130, 7.6%) patients experienced grade 3 or higher irAEs, including grade 4 abnormal hepatic function in 1 patient. No grade 5 irAEs were reported (Supplementary Table 1). Four (3.1%) patients permanently discontinued penpulimab due to TRAEs/irAEs, including increased transaminase levels, disseminated herpes zoster, pemphigoid, and pleural effusion.

**Table 2:** Treatment-related adverse events, all grades (occurring in ≥5% of patients) and grade 3–5

TRAEs	Any grade	Grade 3–5
All	93 (71.5)	14 (10.8)
Serious TRAEs	8 (6.2)	
Treatment interruption due to TRAEs	21 (16.2)	
Permanent treatment discontinuation due to TRAEs	4 (3.1)	
Death due to TRAEs	0 (0.0%)	
TRAEs (≥5%)		
Hypothyroidism	39 (30.0)	0 (0.0)
Anemia	20 (15.4)	1 (0.8)
Aspartate aminotransferase increased	19 (14.6)	1 (0.8)
Blood thyroid stimulating hormone increased	18 (13.8)	0 (0.0)
Alanine aminotransferase increased	16 (12.3)	0 (0.0)
Pyrexia	9 (6.9)	0 (0.0)
Rash	9 (6.9)	2 (1.5)
Proteinuria	8 (6.2)	0 (0.0)
Hyponatraemia	7 (5.4)	0 (0.0)
Gamma-glutamyl transferase increased	4 (3.1)	1 (0.8)
Hepatic function abnormal	4 (3.1)	2 (1.5)
Platelet count decreased	4 (3.1)	1 (0.8)
Ascites	1 (0.8)	1 (0.8)
Death	1 (0.8)	1 (0.8)
Herpes zoster disseminated	1 (0.8)	1 (0.8)
Immune-mediated enterocolitis	1 (0.8)	1 (0.8)
Malnutrition	1 (0.8)	1 (0.8)
Pemphigoid	1 (0.8)	1 (0.8)
Pleural effusion	1 (0.8)	1 (0.8)
Transaminases increased	1 (0.8)	1 (0.8)

### Subgroup analysis

In the subgroup analysis of 60 patients with liver metastases at the baseline, the ORR and DCR were 30.0% (95% CI = 18.8–43.2%) and 40.0% (95% CI = 27.6–53.5%), respectively. The median PFS and OS 1.9 months (95% CI = 1.8–6.6 months) and 18.6 months (95% CI = 9.4–not estimable), respectively. Objective response was observed across all subgroups stratified by demographic and clinical characteristics, except in patients aged ≥65 years (only two patients).

**Fig. 3:**
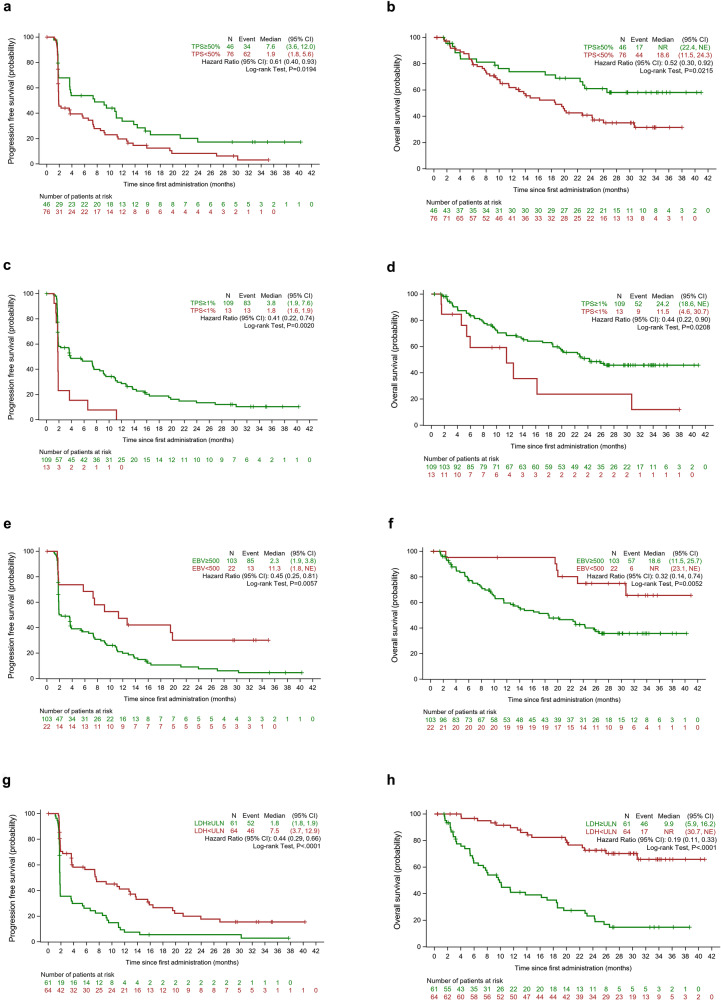
Survival outcomes stratified by key baseline characteristics. Kaplan–Meier-estimated progression-free survival (PFS) curves and overall survival (OS) curves of NPC patients stratified by PD-L1 expression (TPS ≥ 50% vs. <50%) (**a**, **b**) and (TPS ≥ 1% vs. < 1%) (**c**, **d**), EBV DNA levels (≥ 500 IU/mL vs. <500 IU/mL) (**e**, **f**), and baseline lactate dehydrogenase (LDH) levels (≥the upper limit, ULN vs. <ULN) (**g**, **h**)

Among 122 patients assessed for PD-L1 expression, the ORR was 43.5% (95% CI = 28.9–58.9%) *vs*. 19.7% (95% CI = 11.5–30.5%) in the patients with a tumor proportion score (TPS) ≥ 50% (n = 46) *vs*. < 50% (n = 76) (Supplementary Table 2). Higher PD-L1 expression was also associated with longer median PFS (7.6 months, 95% CI = 3.6–12.0 months *vs*. 1.9 months, 95% CI = 1.8–5.6 months; HR = 0.61, 95% CI = 0.40–0.93) as well as OS (not reached, 95% CI = 22.4 months to not estimable *vs*. TPS < 50%: 18.6 months, 95% CI = 11.5–24.3 months; HR = 0.52, 95% CI = 0.30–0.92) (Fig. 3a, b).

In addition, 89.3% of the patients had a TPS ≥ 1% and they had a notably higher ORR than those with TPS < 1% (32.1%, 95% CI = 23.5–41.7% *vs*. 0%). They also had longer median PFS (3.8 months, 95% CI = 1.9–7.6 months *vs*. 1.8 months, 95% CI = 1.6–1.9 months; HR = 0.41, 95% CI = 0.22–0.74) and OS (24.2 months *vs*. 11.5 months; HR = 0.44, 95% CI = 0.22–0.90) (Fig. 3c and d).

Lower baseline EBV DNA levels predicted better efficacy.^24^ Patients with EBV DNA level < 500 IU/mL (n = 22) had higher ORR than those with EBV DNA level ≥ 500 IU/mL (45.5%, 95% CI = 24.4–67.8% *vs*. 24.3%, 95% CI = 16.4–33.7%). Lower baseline EBV DNA level was also associated with longer PFS (11.3 months, 95% CI = 1.8 months to not estimable *vs*. 2.3 months, 95% CI = 1.9–3.8 months; HR, 0.45, 95% CI = 0.25–0.81) and OS (not reached, 95% CI = 23.1 months to not estimable *vs*. 18.6 months, 95% CI = 11.5 to 25.7 months; HR = 0.32, 95% CI = 0.14–0.74) (Fig. 3e, f). Eighty-eight patients were evaluated for EBV DNA levels after two cycles of treatment and 46.6% experienced > 50% decline in EBV DNA levels. Nevertheless, they experienced no notable improvement in PFS and OS compared to those with ≤50% decline or an increase in EBV DNA levels (PFS: 9.1 months *vs*. 1.9 months; OS: not reached *vs*. 30.7 months).

Sixty-one (48.8%) patients had higher baseline lactate dehydrogenase (LDH) levels (≥the upper limit, ULN). Higher baseline LDH level was associated with poor response (ORR = 16.4%, 95% CI = 8.2–28.1% *vs*. 39.1%, 95% CI = 27.1–52.1%), shorter median PFS (1.8 months *vs*. 7.5 months; *P* < 0.001) and OS (9.9 months *vs*. not reached) (Fig. 3g, h).

## Discussion

In this trial, penpulimab monotherapy achieved an ORR of 28.0% (95% CI = 20.3–36.7%), with the lower limit of 95% CI exceeding the prespecified threshold (15%) for trial success. This finding is generally comparable to that obtained for other ICIs in patients with R/M NPC (ORR of 20–25% for pembrolizumab and nivolumab, and 20.5% for toripalimab).^3,10,12^ Notably, the 6-month and 12-month PFS rate in this trial was 42.1% and 24.7%, respectively. The median PFS and OS (3.6 and 22.8 months, respectively) in this trial seemed to be longer than that reported for other ICIs, although direct comparisons between studies are difficult due to the differences in patient characteristics. In patients with R/M NPC, the OS with single-agent chemotherapy was < 15 months.^25^ In the KEYNOTE-122 study, the OS with single chemotherapy was 15.3 months, but 29.3% patients received subsequent immunotherapy. The OS with immunotherapy was slightly prolonged (17.2 months), but not statistically significant.^26^ Considering that all the enrolled patients had received at least two lines of systemic chemotherapy, penpulimab demonstrated notable survival benefit in this heavily pretreated population. Treatment with penpulimab appeared to achieve better long-term (≥6 months) disease control compared to that reported in the CAPTAIN study or KEYNOTE 122 study. However, direct comparisons between studies are difficult and reliable conclusions are hard to draw given differences among studies in patient population characteristics. Though it would be more convincing to compare patient populations with the same or similar characteristics across studies such as ethnicity, disease progression, and PD-L1 level, there is a paucity of data in the literature on a population that closely matches the characteristics of the participants in this study.

Liver metastasis in patients with R/M NPC is associated with poor prognosis and limited response to ICI therapy.^27,28^ In the POLARIS-02 study, the ORR was only 16.8% in patients with liver metastases.^12^ Our subgroup analysis revealed an ORR of 30.0% and a median OS of 18.6 months, indicating that penpulimab may benefit R/M NPC patients with liver metastasis.

Despite a median treatment duration of 4.1 months, the incidence of grade 3 or 4 TRAEs was 11.2% compared to 14.2% for toripalimab to 22.2% for nivolumab.^3,29^ Serious TRAEs occurred in 8 (6.4%) patients. In the KEYNOTE-028 study, the rate of irAEs was 37% for pembrolizumab and 18.5% patients discontinued treatment due to irAEs. Though 48.5% of the patients in this trial developed irAEs, the incidence of grade ≥3 irAEs was low (7.6%). Most grade 3 or 4 irAEs were dermatologic toxicities, with one case each of pneumonia and elevated transaminase levels. Only 4 (3.2%) patients discontinued treatment due to TRAEs/irAEs. The unique properties of penpulimab as an IgG1 antibody with eliminated Fc may have contributed to its favorable safety profile.

Patients with positive PD-L1 expression attained an ORR of 43.5%, and patients with TPS ≥ 1% had an ORR of 32.1% *versus* 0% for those with TPS < 1%, suggesting that tumor response was indeed due to blockade of the PD-1/PD-L1 axis. In addition to PD-L1, EBV DNA and LDH have been identified as predictive biomarkers and patients expressing lower levels of PD-L1 (≤50%) could also benefit from penpulimab. Also consistent with previous studies in R/M NPC,^12,18^ lower LDH level and lower EBV DNA level were associated with higher ORR, longer PFS and OS in this trial.

This trial has several limitations, including the fact that it only involved Chinese subjects and did not include patients with keratinizing tumors due to their rarity in China. Furthermore, only 2 elderly patients (≥65 years) were included. The efficacy and safety profile of penpulimab in patients older than 65 years require further investigation. Additionally, as no standard treatment is currently available for patients with R/M NPC after multiple lines of systemic therapy, we did not include a positive control in this trial. A confirmatory phase III study comparing penpulimab in combination with chemotherapy *vs*. chemotherapy as the first-line treatment for RM/NPC is ongoing (ClinicalTrials.gov identifier: NCT04974398). This phase III trial also does not have a positive control arm since PD-1 inhibitors were not a part of the standard of care in China at the time of trial initiation. Head-to-head comparison of penpulimab with a positive comparator would help determine whether penpulimab is better than or superior to other ICIs.

In conclusion, penpulimab has an acceptable safety profile and durable antitumor activities in heavily pretreated R/M NPC patients. Treatment response was associated with higher PD-L1 expression, lower baseline EBV DNA copy number, and LDH level. R/M NPC patients with hepatic metastasis may benefited from the treatment.

## Materials and methods

### Study design and patients

This single-arm, open-label phase II trial was conducted between March 6, 2019, and September 28, 2022, across 20 centers in China. Patients aged 18–75 years with pathologically confirmed nonkeratinizing metastatic NPC (American Joint Committee on Cancer stage IVb) that had progressed after first-line platinum-based chemotherapy and second-line therapy were eligible. Neoadjuvant, adjuvant, or concurrent chemoradiotherapy was considered as first-line treatment if metastasis occurred within 6 months after the end of the last chemotherapy. Key inclusion criteria included at least one measurable lesion per Response Evaluation Criteria in Solid Tumors (RECIST; version 1.1), adequate organ function, and an Eastern Cooperative Oncology Group (ECOG) performance status (PS) score of 0 or 1. Patients who had previously received penpulimab or other ICIs were excluded. The full eligibility criteria are available in the trial protocol.

The trial was conducted in accordance with the provisions of the Declaration of Helsinki and International Conference on Harmonization Guidelines for Good Clinical Practice. The trial protocol was approved by the institutional ethics committees of all the participating centers (Fudan University Shanghai Cancer Center, approval ID: 1810192-18). All participants provided written informed consent. This trial is registered at ClinicalTrials.gov (NCT03866967).

### Treatment and assessments

Patients received 200-mg penpulimab (Chia Tai Tianqing, China) intravenously every 2 weeks, with 4 weeks per cycle, until disease progression, death, intolerable toxicities, or withdrawal of consent. Patients who had progressive disease (PD), assessed by investigators per RECIST version 1.1, were allowed to continue penpulimab treatment if they continued to benefit from and tolerate penpulimab as deemed by investigators. Penpulimab treatment was interrupted upon grade 2 or 3 TRAEs, and terminated upon grade 3 pneumonitis, liver enzyme abnormalities, and neurotoxicities, and all grade 4 TRAEs.

Best supportive care was provided to all participants. Drugs that may interfere with the study medication were not allowed.

Responses were evaluated radiologically per IRRC using RECIST version 1.1 every 8 weeks for the first 12 cycles and every 12 weeks thereafter. Complete response (CR) and partial response (PR) were confirmed radiologically after at least 4 weeks. Survival was monitored every 3 months. The analysis was performed when the last patient completed at least 24 weeks of follow-up.

### Safety evaluation

AEs were recorded during treatment and until 90 days after the final dose of penpulimab. Safety assessments were based mainly on the occurrence, frequency, and severity of AEs that were graded per National Cancer Institute Common Terminology Criteria for Adverse Events (NCI CTCAE) version 4.03 and coded using MedDRA 22.0. Safety events included AEs and serious AEs (SAEs). IrAEs were TRAEs that were consistent with immune-related causes in the absence of other causes.

### Biomarkers

PD ligand-1 (PD-L1) expression was measured at baseline using SAB028 antibody (Signalway Antibody LLC, MD, USA). A TPS ≥ 50% was considered PD-L1-positive. Plasma Epstein–Barr virus (EBV) DNA was tested every 4 weeks for the first two cycles and every 12 weeks thereafter during treatment and 30 days after the final dose.

### Endpoints

The primary endpoint was the ORR, defined as the proportion of patients who achieved an IRRC-confirmed CR or PR. The secondary endpoints included DCR, defined as the proportion of patients who achieved CR, PR, or stable disease (SD) lasting for at least 4 weeks; duration of response; time to response; progression-free survival (PFS), defined as the time from the first dose to IRRC-confirmed PD or death from any cause, whichever occurred first; OS, defined as the time from the first dose to death from any cause.

### Statistical analysis

The study was considered successful if the lower limit of the 95% confidence interval (CI) for the ORR was ≥ 15% in the full analysis set (FAS) per RECIST version 1.1. The efficacy measures were based on all patients with centrally confirmed NPC who received at least one dose of penpulimab, had at least one measurable lesion according to RECIST 1.1 at baseline, had baseline data and completed at least two follow-up evaluation. For assessment by IRRC, measurable lesion at baseline was determined by IRRC. Assuming an ORR of 26%, one-sample exact test at a one-sided alpha level of 0.025, and 80% power, 110 evaluable patients were needed. This corresponds to the minimum number of observed responses with a lower limit of the 95% CI (Clopper-Person) at >15%. At an expected dropout rate of 15%, 130 patients were required.

Duration of response, PFS, and OS were calculated using the Kaplan–Meier method. Sex was not a biological variable in the trial. The safety set included all patients who received at least one dose of penpulimab. Safety assessments were analyzed using descriptive statistics.

### Supplementary information

Sigtrans_Supplementary_Materials

protocol

## Data Availability

The datasets used and/or analyzed during the current study are available from the corresponding author on request.
