# Supplementary material for: Penpulimab, an anti-PD-1 antibody, for heavily pretreated metastatic nasopharyngeal carcinoma: a single-arm phase II study
Source: Signal Transduct Target Ther. 2024 Jun 19;9:148. doi: 10.1038/s41392-024-01865-6 (PMC11189389; doi:10.1038/s41392-024-01865-6)
Supplement: Supplementary file 1 — Sigtrans_Supplementary_Materials [file 41392_2024_1865_MOESM1_ESM.docx]

Supplementary Materials for

Penpulimab, an anti-PD-1 antibody, for heavily pretreated metastatic nasopharyngeal carcinoma: a single arm phase II study

Xiaozhong Chen, Wei Wang, Qingfeng Zou, Xiaodong Zhu, Qin Lin, Yi Jiang, Yan Sun, Liangfang Shen, Lin Wang, Guorong Zou, Xiaoyan Lin, Shaojun Lin, Minying Li, Ying Wang, Ruilian Xu, Rui Ao, Rensheng Wang, Haifeng Lin, Shuang Huang, Tingting Xu, Wenting Li, Mengying Xia, Yu Xia, Zhongmin Wang, Baiyong Li, Jingao Li, Chaosu Hu

Correspondence to: [hucsu62@163.com](mailto:hucsu62@163.com) and [lijingao@hotmail.com](mailto:lijingao@hotmail.com)

**This PDF file includes:**

Tables S1 to S2

Table S1.

|  | Any grade | Grade 3-5 |
| --- | --- | --- |
| Hypothyroidism | 27 (20.8) | 0 (0.0) |
| Blood thyroid stimulating hormone increased | 16 (12.3) | 0 (0.0) |
| Aspartate aminotransferase increased | 8 (6.2) | 1 (0.8) |
| Rash | 8 (6.2) | 2 (1.5) |
| Alanine aminotransferase increased | 7 (5.4) | 0 (0.0) |
| Anaemia | 7 (5.4) | 1 (0.8) |
| Hepatic function abnormal | 2 (1.5) | 1 (0.8) |
| Pneumonitis | 2 (1.5) | 1 (0.8) |
| Herpes zoster disseminated | 1 (0.8) | 1 (0.8) |
| Immune-mediated enterocolitis | 1 (0.8) | 1 (0.8) |
| Pemphigoid | 1 (0.8) | 1 (0.8) |
| Transaminases increased | 1 (0.8) | 1 (0.8) |

**Immune-related adverse events, all grades (occurring in ≥ 5% of patients) and grade 3-5**

Table S2.

| ORR | Baseline TPS≥50%  N=46 | Baseline TPS<50%  N=76 |
| --- | --- | --- |
| ORR (%, 95% CI) | 43.5 (28.9, 58.9) | 19.7 (11.5, 30.5) |
|  | Baseline TPS≥10%  N=98 | Baseline TPS<10%  N=24 |
| ORR (%, 95% CI) | 33.7 (24.4, 43.9) | 8.3 (1.0, 27.0) |
|  | Baseline EBV≥500 IU/mL  N=103 | Baseline EBV<500 IU/mL  N=22 |
| ORR (%, 95% CI) | 24.3 (16.4, 33.7) | 45.5 (24.4, 67.8) |
|  | LDH≥ULN  N=61 | LDH<ULN  N=64 |
| ORR (%, 95% CI) | 16.4 (8.2, 28.1) | 39.1 (27.1, 52.1) |

**Stratified treatment response in the study subjects**
