# Supplementary material for: Penpulimab, an anti-PD-1 antibody, for heavily pretreated metastatic nasopharyngeal carcinoma: a single-arm phase II study
Source: Signal Transduct Target Ther. 2024 Jun 19;9:148. doi: 10.1038/s41392-024-01865-6 (PMC11189389; doi:10.1038/s41392-024-01865-6)
Supplement: Supplementary file 2 — protocol [file 41392_2024_1865_MOESM2_ESM.pdf]

Clinical Study Protocol

A Single-arm, Open-label, Multicenter Phase II Clinical Study of AK105 in Patients with Metastatic Nasopharyngeal Carcinoma after Failure of Second and Subsequent Lines of Chemotherapy

AK105-202

Version: 3.0, 06 Jul 2020

Akeso Tiancheng Guangdong Co., Ltd.

Confidentiality Statement

Information contained in this document is proprietary to Akeso Tiancheng Guangdong Co., Ltd. Confidential information is provided here in in accordance with the requirements of the Confidentiality and Disclosure Agreement that has been agreed upon and signed. Please do not provide this document or any copy or reveal any proprietary information contained here in to any third party (other than those in your organization who are assisting you in this work and are bound by the Confidentiality and Disclosure Agreement) without prior written permission of an authorized representative of Akeso Tiancheng.

Protocol Summary

Study Title

A Single-arm, Open-label, Multicenter Phase II Clinical Study of AK105 in Patients with Metastatic Nasopharyngeal Carcinoma after Failure of Second and Subsequent Lines of Chemotherapy

Objectives

Primary Objective:

- To evaluate the anti-tumor activity of AK105 in the treatment of metastatic nasopharyngeal carcinoma, as assessed by an Independent Radiology Review Committee (IRRC) based on RECIST v1.1.

Secondary Objectives:

- To evaluate the anti-tumor activity of AK105 in the treatment of metastatic nasopharyngeal carcinoma by the investigator.
- To evaluate the pharmacokinetic profile of AK105 in subjects with metastatic nasopharyngeal carcinoma.
- To evaluate the immunogenicity of AK105 in the treatment of metastatic nasopharyngeal carcinoma.
- To evaluate the safety and tolerability of AK105 in the treatment of metastatic nasopharyngeal carcinoma.
- To evaluate the correlation between EBV-DNA expression in blood and anti-tumor response in nasopharyngeal carcinoma.

Endpoints

Primary Endpoint:

- Objective response rate (ORR) assessed by IRRC per RECIST v1.1 for anti-tumor activity in the Full Analysis Set (FAS) population.

Secondary Endpoints:

- ORR assessed by investigator per RECIST v1.1 for anti-tumor activity in the FAS population.
- Endpoints assessed by IRRC and investigator per RECIST v1.1 include disease control rate (DCR), duration of response (DoR), time to response (TTR), and progression-free survival (PFS).
- Overall survival (OS).
- Pharmacokinetic profile: including the serum concentrations of AK105 in individual subject at different time points after AK105 administration. PK parameters will be measured with non-compartmental analysis, including maximum concentration (C_{max}), AUC, CL, and $t_{1/2}$.
- Immunogenicity assessment: number and percentage of subjects with detectable ADA.
- Safety: incidence and severity of adverse events, abnormal laboratory findings with clinical significance.
- Correlation analysis between the expression of EBV-DNA in blood and the anti-tumor activity of AK105.

Study Design

This study is a multicenter, single-arm, open-label, Phase II clinical study to evaluate the anti-tumor activity, safety, PK and immunogenicity of AK105 in the treatment of metastatic nasopharyngeal carcinoma. Approximately 130 subjects are proposed to be enrolled. The target population of the study is subjects with metastatic stage IVb nasopharyngeal carcinoma who have failed prior first-line platinum-containing chemotherapy and second-line monotherapy or combination chemotherapy.

Based on safety, pharmacokinetic, and pharmacodynamic data from a previous Phase Ia study of AK105, 200 mg once every 2 weeks (Q2W) is determined as the recommended dose for this study. Subjects receive intravenous infusion of AK105 monotherapy until, in the judgment of the investigator, the subject is unable to continue to benefit, the disease progresses, intolerable toxicity occurs, the investigator decides, the subject withdraws the informed consent form（ICF）, or the subject dies, whichever occures first. The study treatment is conducted in a 4-week cycle.

Regular tumor response assessments (per RECIST v1.1) are performed for all subjects, and ORR is the primary efficacy variable. For ongoing subjects, disease progression is assessed and confirmed by the investigator. If the performance status of the subject remains stable, the investigator believes that the subject can benefit from continuous treatment, and the subject meets other criteria listed in the protocol, the subject may continue to receive study treatment until occurrence of intolerable toxicity, no more clinical benefit as judged by the investigator (as assessed by the investigator via radiology, laboratory tests, clinical status), death or loss to follow-up, subject’s withdrawal of consent, and study termination by the sponsor, whichever occurs first.

The primary endpoint of this study is ORR assessed by IRRC per RECIST v1.1 in the FAS population. The analysis of the primary endpoint is performed after the last subject has completed at least 24 weeks of follow-up (an interval between the data cutoff date and the first dose date ≥ 24 weeks) (this follow-up assessment is not required if the subject has achieved radiographic disease progression before 24 weeks). The study will be completed at least 12 months after the first dose of the last subject, and data analysis is performed then. If there are still subjects receiving the study drug treatment at that time, after signing the informed consent form, these subjects can be transferred to an extension study to continue treatment with this study drug until the subjects are unable to continue to benefit as judged by the investigator, experience disease progression and intolerable toxicity, withdraw the informed consent or die, or the study is discontinued due to the investigator's decision.

Target Subjects

Subjects to be included in this study are male or female aged ≥ 18 years and ≤ 75 years, with good organ function and a performance status score of 0 or 1 as assessed by the Eastern Cooperative Oncology Group (ECOG). Histopathologically confirmed nonkeratinizing differentiated or undifferentiated nasopharyngeal carcinoma (WHO Classification II or III). Patients with metastatic nasopharyngeal carcinoma diagnosed with stage IVb [Chinese Nasopharyngeal Carcinoma Staging 2017 (2008 Expert Consensus on Nasopharyngeal Cancer Staging Revision) at enrollment, who are not eligible for radical local therapy， failure of prior first-line platinum-containing chemotherapy (monotherapy or concomitant medication) and second-line monotherapy or concomitant medication. Treatment failure is defined as disease progression occurring during or after chemotherapy. For subjects who have received prior neoadjuvant chemotherapy, concurrent chemoradiotherapy or adjuvant chemotherapy, the original treatment regimen should be the first-line regimen if recurrence/metastasis occurs within 6 months after the end of the last treatment. All treatment changes due to drug intolerance are not considered as treatment failure. Subjects must have at least one measurable lesion (as defined by RECIST v1.1). Subjects agree to provide previously archived tumor tissue samples (tissue samples collected within 3 years prior to enrollment) or undergo biopsies to collect tumor lesion tissue (at least 3 unstained FFPE pathological sections. If the central laboratory determines that the samples are insufficient for PD-L1 IHC testing, 3 additional unstained FFPE pathological sections are required) for PD-L1 immunohistochemistry (IHC) testing by the central laboratory (preferably tumor tissue samples obtained recently).

Treatment Regimen

AK105 is administered by intravenous infusion (200 mg, Q2W), with 4 weeks as a treatment cycle. Until intolerable toxicity occurs, there is no more clinical benefit as judged by the investigator (as assessed by the investigator by radiography, laboratory tests, clinical status), death or loss to follow-up, subject's withdrawal of consent, and study termination by the sponsor, whichever occurs first. The infusion time is 60 min (± 15 min). Ongoing monitoring of potential infusion reactions will be performed and pre-treatment of hypersensitivity and/or adjustment of infusion rate will be allowed. The infusion may be extended to a maximum of 120 min（± 15 min）for subjects unable to tolerate the 60 min infusion.

Statistical Method

Estimation of Sample Size

This study is a single-arm clinical study with the primary endpoint of ORR in the FAS population. The lower limit of the 95% CI for ORR in the FAS population is not less than 15% in the efficacy analysis. If an ORR of 26% is assumed for the efficacy of AK105 in the FAS population, there is 82% power to observe at least 25 CR or PR in 110 evaluable subjects, with the lower limit of the 95% confidence interval not less than 15% (Table 1). Considering dropouts and others, assuming a dropout rate of 15%, a total of approximately 130 subjects are planned to be enrolled to ensure 110 evaluable cases.

Table 1 ORR and 95% CI estimates in 110 evaluable subjects

**Number (%) of subjects achieving CR or PR**	**21(19.1)**	**23(20.9)**	**24(21.8)**	**25(22.7)**	**27(24.5)**	**29(26.4)**

**among 110 subjects**						
Lower limit of 95% CI	12.2%	13.7%	14.5%	15.3%	16.8%	18.4%
Upper limit of 95% CI	27.7%	29.7%	30.7%	31.7%	33.7%	35.6%

Statistical Analysis

The evaluation analysis of efficacy endpoints is based on the FAS, which includes all subjects enrolled in the analysis set who have received at least one dose of AK105 at any dose and have measurable disease at baseline (as defined per RECIST v1.1) and met the primary inclusion criteria: patients with stage IVb at enrollment who have failed first-line platinum-containing chemotherapy (monotherapy or concomitant medication) and second-line monotherapy or concomitant medication. If used for the data analysis of IRRC assessment, the presence or absence of measurable disease should be determined based on the baseline assessed by IRRC. Safety analyses are performed based on the Safety Analysis Set, which included all enrolled subjects who received at least one dose of study drug.

Efficacy Analysis

The primary endpoint of this study is ORR assessed by IRRC per RECIST v1.1. The analysis of the primary endpoint is performed after the last subject has completed at least 24 weeks of follow-up (an interval between the data cutoff date and the first dose date ≥ 24 weeks) (this follow-up assessment is not required if the subject has achieved radiographic disease progression before 24 weeks).

ORR, DCR, and PFS will be analyzed based on the FAS. The exact two-sided 95% confidence intervals for ORR and DCR is estimated with the Clopper Pearson method. Time to event endpoints (DoR, PFS, and OS) will be analyzed using the Kaplan-Meier method. The graphical analysis will include a spider gram of the tumor burden percentage of the target lesion changing from baseline over time, and a waterfall gram of the optimum percentage of the target lesion tumor burden changing from baseline.

Safety

The summary statistical results of AE will be listed according to the seriousness (SAE), severity and causality with the study drug, and AEs and AESIs that caused the discontinuation will also be summarized. AEs will be graded according to version 4.03 of the National Cancer Institute Common Terminology Criteria for Adverse Events (NCI CTCAE), and will be described with the preferred term in the Medical Dictionary for Regulatory Activities (MedDRA). Laboratory abnormalities and toxicity grades will be derived and summarized according to NCI CTCAE (version 4.03), and two-way frequency table for CTCAE grade at baseline and worst post baseline grade will be displayed in shift tables for clinical laboratory tests, and CTCAE Grade 3 or 4 clinical laboratory abnormalities will be presented.

Pharmacokinetics

Individual AK105 concentrations will be tabulated by descriptive statistics. The relevant descriptive statistics of the PK parameters of the non-compartmental model may include AUC, C_{max} , CL, $t_{1/2}$, etc. A population PK model will be established in this trial. The impact of physiological relevant subjects characteristics (covariates) on PK will be evaluated. The relationship between exposure and response or

safety will be evaluated.

Immunogenicity

The immunogenic potential of AK105 will be assessed by summarizing the number and percentage of subjects who develop detectable ADA. The impact of ADA on PK will be assessed if data is available. Samples will be collected for evaluating the neutralizing capacity of ADAs in the future. Only subjects who have received at least one dose of AK105 and provided a baseline sample and at least one post-treatment sample will be evaluated.

Subgroup Analysis

In this study, the cut point of tumor proportion score (TPS) for PD-L1 expression is pre-specified as 50% until the data for subject's individual PD-L1 expression are available (blinded to the individual PD-L1 data). Based on this cut point and PD-L1 expression at baseline, TPS is defined as ≥ 50% in the PD-L1 positive group and < 50% in the negative group. The analysis of ORR, DCR, and PFS will be performed in the PD-L1 subgroup (positive and negative) based on the FAS, respectively. The analytical procedures are described in the Statistical Analysis Plan (SAP).

Interim Analysis

In this study, an interim analysis may be planned after at least 110 subjects have been enrolled and completed at least 16 weeks of follow-up (an interval between the data cutoff date and the date of first dose ≥ 16 weeks), and the results of the interim analysis will likely be used to communicate with CDE. Efficacy analysis will be performed primarily based on the FAS with at least 16-week follow-up (at least 2 response assessments at the time of data cutoff). Safety analyses will be performed based on the Safety Analysis Set. The sponsor does not plan to prematurely discontinue the study based on these efficacy or safety findings of planned analysis.

Table of Contents

Clinical Study Protocol 1
Protocol Summary 2
Table of Contents 7
List of In-text Tables 12
List of In-text Figures 13
List of Abbreviations 14
1. Background 17
1.1. Study Drug 17
1.1.1. Drug Name 17
1.1.2. Description 17
1.1.3. Intended Use Under Investigation 17
1.1.4. Summary of Preclinical Studies 17
1.1.5. Summary of Clinical Studies 19
1.2. Trial Rationale 20
1.3. Study Risk Assessment 22
1.3.1. Potential Risks Associated with AK105 22
1.3.2. Potential Benefits Associated with AK105 23
1.4. Study Hypotheses 23
2. Study Objectives and Endpoints 24
2.1. Objectives 24
2.1.1. Primary Objective 24
2.1.2. Secondary Objectives 24
2.2. Endpoints 24
2.2.1. Primary Endpoint 24
2.2.2. Secondary Endpoints 24
3. Study Design 25
3.1. Overall Design 25
3.1.1. Treatment Regimen 26
3.1.2. Treatment after Progression 26
3.1.3. Study Drug-related Toxicity Management and Dose Modifications 27

3.1.4. Duration of Study......38

3.1.5. Duration of Subject Participation......38

3.1.6. Study Discontinuation Criteria......39

3.2. Study Design Rationale......39

3.2.1. Rationale for Single-arm Studies......39

3.2.2. Rationale for Selection of 200 mg Q2W as Treatment Regimen......39

3.2.3. Rationale for Selection of 24-week Follow-up after the First Dose for the Analysis of the Primary Endpoint......40

3.2.4. Rationale for Treatment after Disease Progression......40

4. Study Population......41

4.1. Enrollment......41

4.1.1. Inclusion Criteria......41

4.1.2. Exclusion Criteria......43

4.2. Subject Withdrawal from Treatment......46

4.2.1. Reasons for Withdrawal......46

4.2.2. Withdrawal Procedure......47

4.2.3. Subject Re-screening Procedures......47

5. Study Drugs and Other Treatments......48

5.1. Study Drug......48

5.1.1. Methods of Assigning Subjects to Treatment Groups and Blinding......48

5.1.2. Preparation......48

5.1.3. Administration of Therapeutic Drugs......49

5.1.4. Monitoring of Dose Administration......49

5.1.5. Labeling and Packaging......50

5.1.6. Storage......50

5.1.7. Drug Accountability......50

5.1.8. Reporting Drug Complaints......51

5.2. Concomitant Medications and Therapies......51

5.2.1. Prohibited Concomitant Medications......51

6. Study Procedures......53

6.1. Enrollment/Screening Period......53

6.2. Treatment Period......54

6.3. End of Treatment and Follow-up Period....63

7. Efficacy Assessments....65

7.1. Tumor Response Assessed per RECIST v1.1....65

7.2. Exploratory Evaluation of Tumor Response Using irRECIST....66

7.3. Survival Assessment....67

8. Pharmacokinetic/Immunogenicity Assessment....68

8.1. Pharmacokinetics....68

8.2. Immunogenicity....69

8.3. Biomarkers....69

8.3.1. Archival Tumor Tissue Samples and Tumor Biopsies....69

8.3.2. Biomarkers in Blood....70

9. Safety Assessments....71

9.1. Adverse Event....71

9.1.1. Definition....71

9.1.2. Recording of Adverse Events....76

9.2. Serious Adverse Event Reporting-Investigator Process....78

9.2.1. Initial Report....78

9.2.2. Follow-up Report....79

9.2.3. Notifying Investigators or Ethics Committee/Institutional Review Board....79

9.3. Other Events Requiring Immediate Reporting....80

9.3.1. Overdose....80

9.3.2. Combined Elevations of Transaminases and Bilirubin....80

9.3.3. Other Adverse Events of Special Interest....80

9.3.4. Reporting and Follow-up of Pregnancy during Clinical Study....81

9.4. Clinical Laboratory Evaluation....81

9.5. Vital Signs....82

9.6. Electrocardiogram....82

9.7. Physical Examination....82

9.8. ECOG Pperformance Sstatus....83

10. Other Aassessments....84

11. Statistical Method....85

11.1. Analysis Set 85
11.1.1. Enrollment Analysis Set *85*
11.1.2. Full Analysis Set *85*
11.1.3. Safety Analysis Set *85*
11.2. General Statistical Considerations 85
11.3. Study Population Data 86
11.4. Efficacy Analysis 86
11.5. Pharmacokinetic/Immunogenicity Analysis 87
11.5.1. Pharmacokinetic Analysis *87*
11.5.2. Immunogenicity Analysis *87*
11.6. Safety Analysis 88
11.6.1. Adverse Events Analysis *88*
11.6.2. Clinical Laboratory Evaluation Analysis *88*
11.6.3. Vital Signs Analysis *88*
11.6.4. Electrocardiogram Analysis *88*
11.7. Biomarker Analysis 89
11.8. Interim Analysis 89
11.9. Sample Size Determination 90
12. Data Integrity and Quality Assurance 91
12.1. Monitoring and Audit 91
12.2. Data Collection 91
12.3. Data Management 92
12.4. Study Documents and Storage 92
12.5. Records Retention 93
13. Ethical and Regulatory Requirements 94
13.1. Ethical Conduct of the Study 94
13.2. Confidentiality of Subjects 94
13.3. Compliance 94
13.4. Informed Consent Process 95
13.5. Changes to the Protocol and Informed Consent Form 96
14. Financing and Insurance 97

14.1. Finance....97

14.2. Reimbursement, Compensation and Insurance....97

15. Publication Policy....98

16. References....99

17. Protocol changes....101

18. Appendices....102

Appendix 1: Response Evaluation Criteria in Solid Tumors Version 1.1 and Iimmune-related Rresponse Eevaluation Ccriteria....102

Appendix 2: Immune-related Response Evaluation Criteria in Solid Tumors (irRECIST)....109

Appendix 3: Listing of Aautoimmune Ddiseases Ppresent prior to Iinclusion....110

Appendix 4: New York Heart Association Classification Criteria for Cardiac Function....111

Appendix 5: Guidelines for Ddiagnostic Aassessment of Iimmune-related Aadverse Eevents....112

Signature Page of Sponsor....114

Investigator's Signature Page....115

List of In-text Tables

Table 1 ORR and 95% CI estimates in 110 evaluable subjects.. 4
Table 2 Treatment modification for infusion-related reactions caused by AK105.................. 29
Table 3 AK105 treatment modification and recommended toxicity management guidelines for immune-related adverse events...31
Table 4 AK105 treatment modification and recommended toxicity management guidelines for non-immune-related reactions... 38
Table 5 Effective methods of contraception (2 methods must be used).................................. 43
Table 6 Screening Procedure Schedule...53
Table 7 Study procedures from Cycle 1 to Cycle 6 during the treatment period......................56
Table 8 Study procedures from Cycle 7 to Cycle 13 and subsequent cycles during treatment 60
Table 9 End of treatment schedule and follow-up procedure.. 63
Table 10 PK sampling schedule for AK105 (intensive sampling)...68
Table 11 PK sampling schedule for AK105 (sparse sampling)..69
Table 12 Criteria for causality determination of adverse drug reactions................................. 75
Table 13 ORR and 95% CI estimates in 110 evaluable subjects... 90
Table 14 Overall response...107

List of In-text Figures

Figure 1 Overall study design chart..25

List of Abbreviations

Abbreviations or Specific Terms	Definition
ADA	Anti-drug antibodies
AE	Adverse event
AESI	Adverse event of special interest
ALP	Alkaline phosphatase
ALT	Glutamic-pyruvic transaminase
ANC	Absolute neutrophil count
AST	Aspartate aminotransferase
AUC	Area under the concentration-time curve
CHO	Chinese hamster ovary
CI	Confidence interval
CL	Clearance
C_{max}	Maximum observed concentration
CNS	Central nervous system
CR	Complete response
CRA	Clinical Research Associate
CRO	Contract research organization
CrCl	Creatinine clearance
CT	Computed tomography
DCR	Disease control rate
DLT	Dose-limiting toxicity
DoR	Duration of response
ECG	Electrocardiogram
ECOG	Eastern Cooperative Oncology Group
eCRF	Electronic case report form
EDC	Electronic data capture
Fc	Crystallizable fragment
FDA	Food and Drug Administration
FFPE	Formalin-fixed paraffin-embedded
GCP	Good Clinical Practice
GLP	Good Laboratory Practice
HBV	Hepatitis B virus
HC	Heavy chain
HCV	Hepatitis C virus
HIV	Human immunodeficiency virus
ICF	Informed consent form
ICH	International Council on Harmonization
IEC	Independent Ethics Committee

**Abbreviations or Specific Terms**	**Definition**
Ig	Immunoglobulins
IgG1	Immunoglobulin G1
IHC	Immunohistochemistry
irAE	Immune-related adverse event
IRB	Institutional Review Board
IRRC	Independent Radiology Review Committee
irCR	Immune-related complete response
irPD	Immune-related progressive disease
irPR	Immune-related partial response
irRECIST	Immune-related Response Evaluation Criteria in Solid Tumors
irSD	Immune-related stable disease
IV	Intravenous
IVIG	Immunoglobulins given intravenously
LC	Light chain
LFT	Liver function test
LLN	Lower limit of normal
mAb	Monoclonal antibodies
MedDRA	Medical Dictionary for Regulatory Activities
MRI	Magnetic resonance imaging
NCI CTCAE	National Cancer Institute Common Terminology Criteria for Adverse Events
NOAEL	No observed adverse effect level
NPC	Nasopharyngeal carcinoma
NSAID	Non-steroidal anti-inflammatory drug
ORR	Objective response rate
OS	Overall survival
PD	Progressive disease
PD-1	Programmed cell death-1
PD-L1	Programmed cell death ligand-1
PD-L2	Programmed cell death ligand-2
PFS	Progression-free survival
PK	Pharmacokinetics
PR	Partial response
Q2W	Once every 2 weeks
Q8W	Once every 8 weeks
QTcB	QT interval corrected for heart rate using Bazett's formula
QTcF	QT interval corrected for heart rate using Fridericia's formula
RECIST	Response Evaluation Criteria in Solid Tumors
RO	Receptor occupancy

Abbreviations or Specific Terms	Definition
SAE	Serious adverse event
SAP	Statistical analysis plan
SD	Stable disease
SID	Subject identification
$t_{1/2}$	Half-life
TTR	Time to response
TBil	Total bilirubin
TIL	Tumor infiltrating lymphocyte
TSH	Thyroid stimulating hormone
ULN	Upper limit of normal
WHO-DD	World Health Organization Drug Dictionary

1. BACKGROUND

1.1. Study Drug

1.1.1. Drug Name

AK105

1.1.2. Description

AK105 is a human immunoglobulin G1 (IgG1) monoclonal antibody (mAb) that directly acts on human programmed cell death-1 (PD-1). AK105 can effectively block the binding of human PD-1 to programmed cell death protein ligand-1 (PD-L1) and programmed cell death protein ligand-2 (PD-L2).

AK105 has a typical antibody structure consisting of 2 heavy chains of the IgG1 subclass and 2 light chains of the κ subclass, which are covalently linked through disulfide bonds. The AK105 antibody carrying crystallizable fragment (Fc) is specifically mutated in amino acids by bioengineering technology to effectively remove the binding capacity to Fcγ receptor I, thereby, immune cell damage due to ADCC effects and possible attenuation of the anti-tumor effect of PD-1 antibodies are avoided (**Dahan R, 2015**).

AK105 is expressed in a Chinese hamster ovary cell line with a total molecular weight (including that of oligosaccharides) of approximately 150 kDa. Each heavy chain is composed of 448 amino acids with a molecular weight of 48923.56 Da (theoretical without glycosylation). Each light chain is composed 214 amino acids with a molecular weight of 23,598.38 Da.

1.1.3. Intended Use Under Investigation

The intended indication is metastatic nasopharyngeal carcinoma after failure of second and subsequent lines of chemotherapy.

1.1.4. Summary of Preclinical Studies

Please refer to the Investigator's Brochure for specific preclinical study results.

1.1.4.1. Summary of Preclinical Pharmacokinetics

The pharmacokinetic (PK) profile of AK105 was investigated in cynomolgus monkeys. After AK105 was injected at doses of 1, 3, and 10 mg/kg, the clearance (CL) observed in males and females was 0.33 and 0.32 mL/h/kg, 0.32 and 0.27 mL/h/kg, 0.25 and 0.20 mL/h/kg, respectively. In males and females, the distribution volume of AK105 at 1, 3 and 10 mg/kg was 57.40 mL/kg and 63.69 mL/kg, 54.73 mL/kg and 62.54 mL/kg, 22.04 mL/kg and 44.01 mL/kg, respectively. In males and females, the half-life ($t_{1/2}$) of AK105 at 1, 3, and 10 mg/kg was 122.80 h and 140.31 h, 121.65 h and 166.94 h, 64.03 h and 167.16 h, respectively.

Following intravenous administration of 1, 3, and 10 mg/kg to cynomolgus monkeys, the mean C_{max} ratio of AK105 was 1:2.94:13.03 and 1:2.90:12.01 in males and females, respectively. The mean AUC_{0-t} ratio of AK105 in male and female monkeys was 1:3.14:14.63

and 1:4.07:19.44, respectively. These results demonstrated that the PK of AK105 was linear from 1 to 10 mg/kg. There were no significant differences in PK parameters of AK105 between female and male monkeys.

In the repeat-dose toxicology study, toxicokinetic parameters were evaluated in cynomolgus monkeys following intravenous infusion of AK105 (4, 15, and 60 mg/kg) once every 2 weeks for 6 weeks (3 doses). The $AUC_{0\text{-}336h}$ ratio of the last dose to the first dose of AK105 was 0.55, 0.70 and 1.35 in males and 0.05, 1.07 and 1.03 in females at the dose of 4, 15, and 60 mg/kg, respectively. There was no obvious accumulation, but the systemic exposure decreased significantly for females at 4 mg/kg after repeat administration. These results suggested that the systemic exposure to AK105 (in females at 4 mg/kg) might be reduced by the production of anti-drug antibodies (ADA) or that the bioanalytical results were interfered with by ADA.

1.1.4.2. Summary of Preclinical Pharmacology

PD-1 is a member of the Ig superfamily involved in the regulation of T cell activation, and this receptor is found in T cells, B cells, macrophages, NK cells, dendritic cells, and mast cells. PD-1 is involved in the inhibition of T cells activation and downstream effects of cytokine production, proliferation, cell survival, and transcription factors associated with effector T cell function.

AK105 exhibits the following activities as a PD-1 monoclonal antibody:

- AK105 binds to human PD-1 with high affinity, and the dissociation rate of AK105 from PD-1 is significantly decreased, suggesting that AK105 binds to target antigen more stably.
- AK105 can effectively block the human PD-1/PD-L1 and PD-1/PD-L2 interactions.
- AK105 can significantly increase the secretion of IL-2 and IFN-γ by human primary T cells co-cultured with cells expressing PD-L1 (e.g., dendritic cells).
- AK105 significantly inhibits tumor growth in a SCID/Beige mouse Raji-PL-L1 cell xenograft model, in which Raji-PD-L1 is co-injected with human peripheral blood mononuclear cells (PBMCs) into immunocompromised mice to determine the inhibition of AK105 on the growth of inoculated tumor cells.
- AK105 inhibits the growth of mouse colorectal cancer tumors in a PD-1 knock-in mouse colorectal cancer cell (MC38 cell) xenograft model.

1.1.4.3. Summary of Preclinical Toxicology

Cynomolgus monkeys are considered to be the relevant animal species for nonclinical safety studies with AK105. A 6-week repeat-dose toxicity study was performed in cynomolgus monkeys to explore the safety of AK105 in accordance with Good Laboratory Practice (GLP), with a maximum dose tested of 60 mg/kg/2 weeks.

AK105 was still well tolerated by intravenous bolus at doses up to 60 mg/kg. Treatment-related histopathological changes in the kidneys (increased pigment in renal

cortical tubules or pigment interstitial macrophages) were observed in some animals but had no effect on renal function. No specific effects was identified on vital functions, including cardiovascular system, central nervous system and respiratory system. Under the conditions of the study, the no-observed-adverse-effect-level (NOAEL) was 60 mg/kg.

1.1.5. Summary of Clinical Studies

Subjects with advanced solid tumors who have failed standard of care were enrolled in the Phase Ia dose-escalation trial of AK105-101 in Australia, using a classical “3 + 3” design, with three dose levels (1 mg/kg, 3 mg/kg, and 10 mg/kg, Q2W) evaluated. As of February 12, 2019, a total of 16 advanced subjects who have failed multiple treatments in the 1 mg/kg (n = 3), 3 mg/kg (n = 6), and 10 mg/kg (n = 7) dose groups have been enrolled, and 14 of these subjects completed at least one post-dose tumor imaging assessment, and preliminary results showed that ORR was 29% and DCR was 57%, with long-term responses and continued treatment for response. A total of 18 subjects were enrolled in the Phase Ib extension study with colorectal cancer (n = 1), gastric or gastroesophageal adenocarcinoma (n = 8), liver cancer (n = 4), esophageal squamous cell carcinoma (n = 5), and received AK105 at a fixed dose of 200 mg Q2W. 9 subjects with advanced disease who have failed multiple treatments had at least one post-dose tumor imaging assessment, with an ORR of 22% and a DCR of 67%. The median number of doses administered to 34 subjects was 5, with a maximum of 29, of which 14 (41%) subjects experienced treatment-related adverse events (TRAEs), 4 (12%) subjects experienced drug-related Grade 3 adverse events, no Grade 4 adverse events were reported, and no DLT events occurred. 3 (9%) subjects had dose interruption due to AEs and no AEs led to dose discontinuation. The most common TRAEs (> 5%) were hyperthyroidism (9%), hypothyroidism (6%), fatigue (6%), and rash (6%). There was no report of any grade of drug-related colitis or pneumonia.

Similar PD-1/PD-L1 inhibitors have been extensively studied in various malignancies. These clinical studies quickly obtained major breakthrough results, and based on those study, PD-1/PD-L1 inhibitors (currently including atezolizumab, nivolumab, pembrolizumab, avelumab, and dulvacizumab) have been approved by FDA for the treatment of multiple malignancies. Four PD-1 inhibitors have been approved for marketing in China, of which nivolumab is indicated for the treatment of adult patients with locally advanced or metastatic non-small cell lung cancer (NSCLC) who are negative for epidermal growth factor receptor (EGFR) gene mutation and negative for anaplastic lymphoma kinase (ALK), have disease progression after prior platinum-containing chemotherapy, or are intolerable for platinum-containing chemotherapy. Pembrolizumab is indicated for the treatment of unresectable or metastatic melanoma after failure of first-line therapy, toripalimab for unresectable or metastatic melanoma after failure of prior systemic therapy, and sintilimab for recurrent or refractory classical Hodgkin lymphoma after at least second-line systemic chemotherapy. At present, no PD-1 inhibitors have been approved for the treatment of nasopharyngeal carcinoma in China. Refer to the package inserts of similar PD-1/PD-L1 inhibitors for safety and efficacy at home and abroad.

1.2. Trial Rationale

Nasopharyngeal carcinoma (NPC) is a kind of malignancy arising in the top or lateral wall of nasopharyngeal cavity. Its epidemiology has obvious regional characteristics, and it is a tumor type with high incidence in Southeast Asia. In Southeast Asia, the predominant histologic type is nonkeratinizing differentiated or undifferentiated carcinoma (WHO classification type II and III) with an incidence of approximately 4.3/100,000 persons/year. In western population, the squamous cell carcinoma is more common (WHO classification type I) with an incidence of < 1%/100,000 persons/year. On the basis of high-level evidence, radiotherapy or chemoradiotherapy has become the mainstay of treatment for patients with early or locally advanced nasopharyngeal carcinoma, and the 5-year survival rate is approximately 85%. However, tumor metastasis remains the leading cause of treatment failure in these patients. Moreover, approximately 15% of NPC patients develop distant metastases at initial diagnosis, and patients with recurrent/metastatic nasopharyngeal carcinoma have a very poor prognosis, with a median overall survival of approximately 20 months.

Platinum-containing chemotherapy is generally considered the standard of care for patients with recurrent/metastatic nasopharyngeal carcinoma. Currently, cisplatin in combination with continuous intravenous infusion of fluorouracil is widely used in patients with recurrent/metastatic NPC, and available Phase II studies have reported that objective response rates (ORR) ranged from 54% to 78% and the median progression-free survival was 7 to 11 months (**Ngan R, 2002**). However, short-term reaction, common mucosal complications, and requirements for deep venous catheterization remain major limitations to the fluorouracil plus cisplatin regimen. A Phase III randomized controlled study demonstrated that gemcitabine plus cisplatin significantly improved the objective tumor response rate from 42% to 64% and the median progression-free survival from 5.6 months to 7.0 months compared to fluorouracil plus cisplatin (**Zhang L, 2016**).

For patients who have failed platinum-containing first-line chemotherapy, the subsequent second-line therapy depends on patient performance status, toxicity, and the time interval to recurrence after a platinum-containing regimen. For patients who have failed platinum-containing first-line chemotherapy but have no disease progression for more than 1 year, the efficacy of re-treatment with platinum-containing combination chemotherapy may be considered. Carboplatin may be an acceptable alternative to cisplatin, but it usually results in more hematologic toxicity. For patients who have failed first-line platinum-containing chemotherapy and whose disease has recurrent within one year after the chemotherapy regimen, the second-line chemotherapy, including gemcitabine, capecitabine, or paclitaxel, may be considered. However, to date, there is no recognized standard third-line systemic therapy. Increasing evidence suggests that further use of chemotherapy after second- or third-line chemotherapy may not prolong the significant meaningful survival in most patients. The development of new therapies in NPC has been somewhat slow, with little progress excepting for standard cytotoxic approaches and only a few exploratory phase II studies. A multicenter Phase II study evaluating the combination of cetuximab and carboplatin in

platinum-resistant recurrent NPC (**Chan A, 2005**) showed an ORR of 11.7%, with acceptable safety profile. However, ORR and PFS did not appear to be superior to chemotherapy alone. A Phase II study evaluating the effect of pazopanib (an anti-angiogenesis inhibitor) in patients with recurrent or metastatic NPC who have failed at least one chemotherapy (**Lim WT, 2011**) showed an ORR of 6.1%, SD in more than 50% of patients, and PR/SD persisted for at least 6 months in 21% of patients, but there were two Grade 5 events of tumor hemorrhage.

It has been reported that NPC is closely associated with EB virus infection, and there is high PD-L1 expression in the tumor, suggesting that PD-1 blockade may be an ideal treatment option for NPC. PD-1 is a negative immunomodulatory molecule that mediates tumor immunosuppression in the local tumor microenvironment. PD-1 is expressed on the surface of activated T cells with two ligands, PD-L1 and PD-L2 (**Sharpe AH, 2007**). PD-L1 is widely expressed on the surface of antigen-presenting cells (APC) and tumor cells, and the expression of PD-L1 is mainly upregulated by interferon produced by effector T cells (**Schreiner B, 2004; Nakae S, 2006**). The binding of PD-1 and PD-L1 significantly inhibits T cell proliferation and activation, ultimately inducing antigen-specific T cell apoptosis to prevent peripheral tissue damage and autoimmune diseases (**Okazaki T, 2006**). Tumor cells inhibit anti-tumor immunity by blocking the PD-1/PD-L1 pathway, and it has been reported in previously published studies that multiple cancer cells escape immune surveillance by upregulating PD-L1 (**Chen, L, 2004**). PD-L1 overexpression has been reported in several clinical studies to be associated with poor prognosis in several types of tumors, including renal cell carcinoma, bladder cancer, esophageal cancer, gastric cancer, hepatocellular cancer, and ovarian cancer (**Wang X, 2016**). These findings suggest that blocking PD-1 signaling may improve the prognosis of patients with these malignancies.

PD-1 blockade therapy has unique characteristics compared to standard anti-tumor therapies. Conventional chemotherapy usually targets specific molecules in tumor cells. Tumor cells can evade treatment by mutating target molecules, leading to rapid disease deterioration. However, PD-1 blockade is indicated for various types of cancer and can maintain a therapeutic response for longer periods of time because it can activate the anti-tumor immune system, targeting the mutant protein (**Tran E, 2017**). In addition, PD-1 blockade therapy has significantly lower higher-grade toxicity compared to other immunotherapies or standard of care because the anti-tumor immunity preferentially recognizes tumor antigens rather than autoantigens (**Iwai Y**, **2002**).

The KEYNOTE-028 Phase Ib study (NCT 02054806) evaluated the clinical efficacy of the immune checkpoint blocker pembrolizumab in the treatment of recurrent/metastatic NPC (**Hsu C, 2017**). 27 patients with PD-L1-positive recurrent/metastatic NPC who were treated with 10 mg/kg pembrolizumab every 2 weeks achieved an ORR of 25.9% and a median duration of response of 17.1 months as assessed by the investigator, one additional patient had an unconfirmed partial response. Confirmed partial response was in 5 of 19 (26.3%) subjects as assessed by IRRC. The median overall survival with pembrolizumab for recurrent/metastatic NPC was 16.5 months and the overall 1-year survival rate was 63%. In

some other cytotoxic and non-cytotoxic drug studies, the overall 1-year survival was reported to be approximately 45%.

A Phase II study (NCT02339558) evaluated the clinical efficacy of the immune checkpoint blocker nivolumab in the treatment of recurrent/metastatic NPC (**Ma BBY, 2018**). 44 patients with recurrent/metastatic NPC who were treated with 3 mg/kg nivolumab every 2 weeks achieved an ORR of 20.5%, a median duration of response of 9.3 months, a median overall survival of 17.1 months, and an overall 1-year survival rate of 59% as assessed by the investigator. The ORR was 33% in 18 PD-L1-positive patients and 13% in 23 PD-L1-negative patients.

The latest clinical progress and data of the anti-PD-1 monoclonal antibody JS001 on multiple indications were reported by TopAlliance Biosciences at the 2017 Chinese Society of Clinical Oncology (CSCO 2017) (CDE Registration No. CTR20160740). As of September 11, 2017, 39 NPC patients were evaluable for response, of which 12 had PR, 13 had SD, with an ORR of 30.8% and a DCR of 64.1%. Of the 19 PD-L1-positive patients, ORR reached 42.1% and DCR reached 73.7%. In 20 PD-L1-negative patients, the clinical response rate was slightly lower, but there was still an ORR of 20% and a DCR of 55%.

AK105 is a human IgG1 monoclonal antibody that binds to PD-1 and blocks the interactions of PD-1 with PD-L1 and PD-L2, thereby inhibiting immunosuppressive responses (including anti-tumor immune responses) mediated via the PD-1 pathway. In view of the recent significant efficacy obtained from similar PD-1 inhibitors in patients with advanced recurrent or metastatic NPC who have failed standard therapy, it is suggested that AK105 may also achieve good efficacy in similar patients, bringing a new treatment modality for Chinese patients with NPC.

Therefore, the purpose of this study is to evaluate the efficacy and safety of AK105 in patients with metastatic NPC after failure of second and subsequent lines of chemotherapy, and to determine its PK and immunogenicity characteristics, so as to provide a basis for subsequent studies.

1.3. Study Risk Assessment

1.3.1. Potential Risks Associated with AK105

Potential risks include immune-mediated reactions (immune-related adverse events, irAEs) based on the mechanism of action of AK105 and similar PD-1/PD-L1 blockers (e.g., pembrolizumab, nivolumab, atezolizumab, durvalumab, and avelumab). irAEs, including immune-mediated colitis, dermatitis, pneumonia, hepatitis, encephalitis, nephritis, and endocrine disorders, may be similar to those with anti-PD-1/L1 inhibitor (**Boutros C, 2016**). According to the available clinical data of anti-PD-1 monoclonal antibody drugs, although the incidence of adverse reactions is high, these antibodies are well tolerated, and only a small proportion of subjects may discontinue the drug due to adverse reactions, and most adverse reactions can be resolved with treatment.

Similar to some therapeutic antibodies, other potential risks associated with AK105 include infusion-related reactions or allergic reactions. Subjects may develop ADA that neutralizes AK105 and causes infusion reactions or systemic allergic reactions, or may induce or enhance the toxicity of AK105. Standard clinical assessments and interventions for infusion-related reactions or systemic allergic reactions are recommended.

The proposed exclusion criteria, safety monitoring, discontinuation criteria, and toxicity management guidelines (Section 3.1.3) are intended to minimize the potential risk for patients participating in this study. The study specifies exclusion criteria to ensure that subjects who may be at risk will not be included (i.e., patients with prior autoimmune diseases, inflammatory bowel disease, unresolved toxicities from prior therapies, blood disease or organ dysfunction will not be included to avoid exacerbations of pre-existing disease due to immune system activation). Toxicity management guidelines have been developed based on other anti-PD-1/L1 agents, with a focus on frequent monitoring and early detection of potential immune-related adverse events. Measures used to manage toxicity include dose interruption and/or use of glucocorticoids or more effective immunosuppressants as needed.

1.3.2. Potential Benefits Associated with AK105

Phase Ia clinical data of AK105 have preliminarily shown that AK105 is safe and well tolerated in patients with advanced tumors, and its anti-tumor activity and definite pharmacological activity are preliminarily observed. In view of the recent significant efficacy obtained from similar PD-1 inhibitors in patients with platinum-resistant recurrent or metastatic NPC, it is suggested that AK105 may also achieve good efficacy in these patients, and treatment with AK105 may achieve response and prolonged PFS.

1.4. Study Hypotheses

The hypothesis of this study is that AK105 is safe, tolerable and has significant anti-tumor activity in patients with metastatic nasopharyngeal carcinoma after failure of second and subsequent lines of chemotherapy.

2. STUDY OBJECTIVES AND ENDPOINTS

2.1. Objectives

2.1.1. Primary Objective

- To evaluate the anti-tumor activity of AK105 in the treatment of metastatic nasopharyngeal carcinoma, as assessed by IRRC based on RECIST v1.1.

2.1.2. Secondary Objectives

- To evaluate the anti-tumor activity of AK105 in the treatment of metastatic nasopharyngeal carcinoma by the investigator.
- To evaluate the pharmacokinetic profile of AK105 in subjects with metastatic nasopharyngeal carcinoma.
- To evaluate the immunogenicity of AK105 in the treatment of metastatic nasopharyngeal carcinoma.
- To evaluate the safety and tolerability of AK105 in the treatment of metastatic nasopharyngeal carcinoma.
- To evaluate the correlation between EBV-DNA expression in blood and anti-tumor response in nasopharyngeal carcinoma.

2.2. Endpoints

2.2.1. Primary Endpoint

- ORR assessed by IRRC per RECIST v1.1 for anti-tumor activity in the Full Analysis Set (FAS) population.

2.2.2. Secondary Endpoints

- ORR as assessed by investigator per RECIST v1.1 for anti-tumor activity in the FAS population.
- Endpoints assessed by IRRC and investigator per RECIST v1.1 include disease control rate (DCR), duration of response (DoR), time to response (TTR), and progression-free survival (PFS).
- Overall survival (OS).
- Pharmacokinetic profile: including the serum concentrations of AK105 in individual subject at different time points after AK105 administration. PK parameters will be measured with non-compartmental analysis, including maximum concentration (C_{max}), AUC, CL, and $t_{1/2}$.
- Immunogenicity assessment: number and percentage of subjects with detectable ADA.
- Safety: incidence and severity of adverse events, abnormal laboratory findings with clinical significance.
- Correlation analysis between the expression of EBV-DNA in blood and the anti-tumor activity of AK105.

3. STUDY DESIGN

3.1. Overall Design

This is a multicenter, open-label, single-arm, Phase II clinical study to evaluate the anti-tumor activity, safety, PK and immunogenicity of AK105 in the treatment of metastatic nasopharyngeal carcinoma. Approximately 130 subjects are proposed to be enrolled in this study. The target population of the study is subjects with metastatic nasopharyngeal carcinoma who have failed prior first-line platinum-containing chemotherapy and second-line monotherapy or combination chemotherapy.

Based on safety, pharmacokinetic, and pharmacodynamic data from a previous Phase Ia study of AK105, 200 mg once every 2 weeks (Q2W) is determined as the recommended dose for this study (see Section 3.2.2 for dose selection rationale). Subjects receive intravenous infusion of AK105 monotherapy until the subject is unable to continue to benefit judged by the investigator, the disease progresses, intolerable toxicity occurs, the investigator decides, the subject withdraws the informed consent form（ICF）, or the subject dies, whichever occurs first. The study treatment is conducted in a 4-week cycle.

Figure 1 Overall study design chart

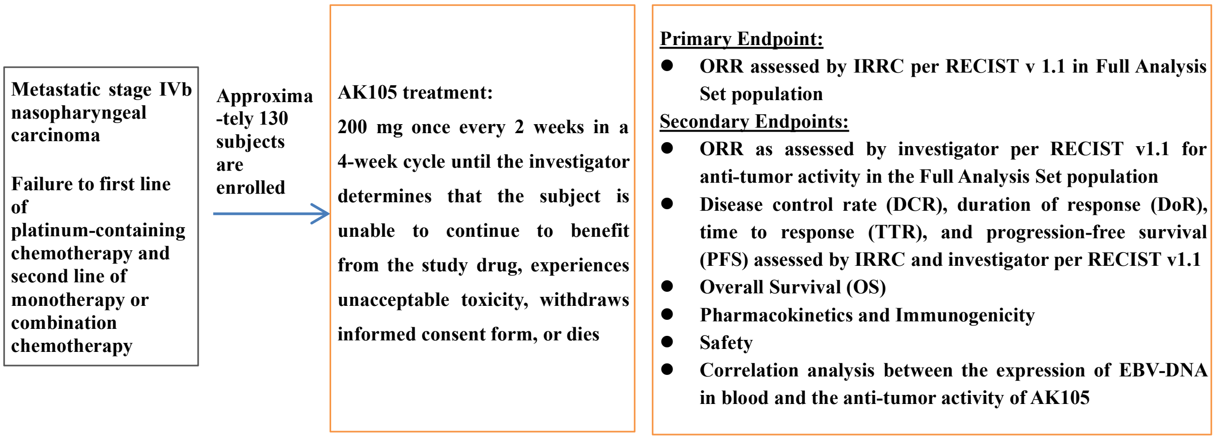

Regular tumor response assessments (using RECIST v1.1) will be performed for all subjects, and ORR is the primary efficacy variable in the FAS (refer to Section 7 for description of tumor imaging assessments). For onging subjects, disease progression is assessed and confirmed by the investigator per RECIST v1.1. If the performance status of the subject remaines stable, the investigator believes that the subject can benefit from continuous treatment, and the subject meets other criteria (see Section 3.1.2) listed in the protocol, the subject may continue to receive study treatment until intolerable toxicity occurs, no longer clinical benefit as judged by the investigator (as assessed by the investigator via radiology, laboratory tests, clinical status), death or loss to follow-up, subject’s withdrawal of consent, and study termination by the sponsor, whichever occurs first. Endpoints to be measured in this study are presented in Section 2.2.

The primary endpoint is ORR as assessed by IRRC per RECIST v1.1. Analysis of the primary endpoint is performed after the last subject has completed at least 24 weeks of follow-up (≥

24 weeks between the data cutoff date and the date of the first dose) (this follow-up assessment is not required if the subject has achieved radiographic disease progression before 24 weeks). The study will be completed at least 12 months after the first dose of the last subject, and data analysis is performed then. If there are still subjects receiving the study drug treatment at that time, after signing the ICF, these subjects can be transferred to an extension study to continue treatment with this study drug until the subjects are unable to continue to benefit as judged by the investigator, experience disease progression and intolerable toxicity, withdraw the informed consent or die, or the study is discontinued due to the investigator's decision.

3.1.1. Treatment Regimen

AK105 monotherapy (200 mg, Q2W, IV) is administrated until the subject is unable to continue to benefit as judged by the investigator (The investigators are assessed by radiography, laboratory tests, and clinical status), experiences intolerable toxicity, starts a new anti-tumor therapy, withdraws ICF, dies, lost to follow-up, or other conditions that require discontinuation as specified in the protocol (Whichever occurs first). The study treatment is conducted in a 4-week cycle. AK105 will be administered as an infusion within 60 min (± 15 min). Ongoing monitoring of potential infusion reactions will be performed and pretreatment of hypersensitivity and/or adjustment of infusion rate will be allowed per protocol guidelines (Section 3.1.3.1and Table 2). The infusion may be extended to a maximum of 120 min (± 15 min) for subjects unable to tolerate the 60 min infusion. Dose modification is not allowed for AK105, but dose delay is allowed up to 8 weeks, calculated from the date when the last dose is administered, otherwise the treatment should be discontinued. If the dose is delayed within 3 days or, in exceptional cases, for more than 3 days but less than 7 days, the actual dosing time is recommended to be used for the calculation of the time window of next dose, and then time window of subsequent dosing thereafter is still within 3 days. If the delay exceeds 7 days, it is recommended that this dose is discontinued and the next dose should be continued on the scheduled dosing date.

3.1.2. Treatment after Progression

Subjects treated with AK105 are allowed to continue study treatment if they are assessed as clinically beneficial and tolerated to the study drug after disease progression assessed by the investigator per RECIST v1.1 (see Section 3.1.2 Rationale for Treatment after Disease Progression). Until the subject is unable to continue to benefit as judged by the investigator (The investigators are assessed by imaging, laboratory tests, and clinical status), experiences intolerable toxicity, starts a new anti-tumor therapy, withdraws ICF, dies, lost to follow-up, or other conditions that require discontinuation as specified in the protocol (Whichever occurs first).

During the treatment with AK105, subjects who are firstly judged as disease progression (initial disease progression) by investigator per RECIST v1.1 must meet all of the following criteria if they continue to receive AK105 treatment:

- The investigator assesses that continued treatment can still provide clinical benefit to subjects (eg, immune-related partial response (irPR); immune-related stable disease (irSD)) (**Appendix 1**); the assessment of clinical benefit should take into account whether the subject has experienced clinical deterioration and is unlikely to benefit further from continued treatment.
- The subject can tolerate the study drug.
- ECOG PS score is stable;
- Management of serious complications requiring urgent intervention (e.g., metastases to the CNS) will not be delayed;
- Before continuing with AK105, the subject needs to be fully informed and the investigator needs to elaborate on all foreseeable risks or discomfort and alternative treatments.

A scan to confirm progression should be performed at the next follow-up and within not less than 4 weeks of the initial assessment of disease progression (without clinical deterioration).

Progression can be confirmed if the following criteria (based on irRECIST) are met:

- The overall tumor burden (sum of the diameters of target lesions and measurable new lesions) in 2 consecutive visits is ≥ 20% from the lowest post-dose overall tumor burden value and increases by an absolute value of 5 mm from the lowest value;
- and/or compared to non-target lesions or non-measurable new lesions when the first progression is observed, there is significant definite progression (worsening) in non-target lesions or non-measurable new lesions (including additional non-measurable new lesions) when the imaging assessments are performed to confirm progression.

New lesions will be considered measurable at the time of initial progression if the longest diameter is ≥10 mm (excluding pathological lymph nodes, which should have a minimum of 15 mm in the short diameter). Any new lesion assessed non-measurable at the time of initial progression may become measurable if its longest diameter increases to more than 10 mm (excluding pathological lymph nodes, whose short diameter must increase to more than 15 mm) , and therefore needs to be included in tumor burden measurements.

3.1.3. Study Drug-related Toxicity Management and Dose Modifications

Refer to Table 2, Table 3, and Table 4 for guidelines on treatment modification and toxicity management of infusion-related reactions, immune-related AEs, and non-immune-related AEs. Additionally, guidelines for treatment modification and toxicity management of other immune-related AEs not mentioned in the tables above can be found in management of immune-related adverse events in patients treated with checkpoint inhibitors: American Society of Clinical Oncology Clinical Practice Guidelines (**Brahmer JR, 2018**).

3.1.3.1. Infusion Reactions

Subjects will be monitored for signs and symptoms of infusion-related reactions (eg, fever and/or chills, flushing and/or pruritus, changes in heart rate and blood pressure, dyspnea or chest discomfort, rash, etc.) and systemic allergic reactions (eg, generalized urticaria, angioedema, asthma, hypotension, tachycardia).

In this study, to avoid confusion of potential safety signals regarding the assessment of infusion reactions:

1) Primary prophylaxis for infusion reactions (prophylaxis in subjects without infusion events) is not permitted.

2) Appropriate secondary prophylaxis (i.e., prevention of infusion-related reactions after the initial episode) should be performed at the discretion of the investigator:

   - Acetaminophen/paracetamol and/or antihistamines (e.g. diphenhydramine) may be administered within approximately 30 min prior to the start of subsequent infusions, and/or glucocorticoids or equivalents may be administered according to the routine diagnosis and treatment of each site.
   - Non-sedating antihistamines (eg, cetirizine) may be considered for subjects with recurrent infusion reactions.
   - After these treatments above, glucocorticoid therapy should be considered if symptoms persist.
   - In accordance with the routine of diagnosis and treatment of each site, pethidine/morphine sulfate and promethazine or their equivalents may be administered intravenously at the discretion of the investigator prior to the start of subsequent infusions.

Reuse of pretreatment medications may be required during infusion; therefore, this should be taken into account when deciding the dose of the pretreatment medication. Any major reactions and use requirements related to glucocorticoids must be discussed with the medical monitor.

In the event of ≤ Grade 2 infusion-related reactions, the infusion rate of AK105 may be reduced by 50%, or infusion may be interrupted until resolution of the event (up to 4 hours)， and infusion can be resumed at an initial infusion rate of 50% after resolution of the event until the infusion is completed. Once the infusion rate of AK105 is reduced by 50% or the infusion is interrupted due to an infusion-related reaction, and it is recommended that all subsequent infusions must be continued at a reduced infusion rate. If a subject experiences a second infusion reaction of ≥ Grade 2 at a slower infusion rate, the infusion should be discontinued and the subject must discontinue AK105 treatment. If a subject experiences a Grade 3 or 4 infusion reaction at any time, AK105 treatment must be discontinued.

If a serious hypersensitivity reaction (CTCAE Grade 3 or 4) is observed during the infusion, the infusion will be stopped immediately and the subject will no longer be given further AK105 treatment. Supportive care should be provided according to standard medical practice. Concerning the complete guidelines of the Resuscitation Council (UK) Working Group on the emergency treatment of systemic anaphylactic reactions, please visit https://www.resus.org.uk/anaphylaxis/emergency-treatment-of-anaphylactic-reactions/. If a subject experiences symptoms or signs of systemic allergic reactions or severe type I hypersensitivity reactions during AK105 administration, the subject will be treated with appropriate medications and medical equipment, which should be readily available at all study sites. Glucocorticoids, epinephrine, anti-allergic agents (antihistamines) or equivalent drugs should be readily available. Subjects should be instructed to report any delayed reactions immediately to the investigator.

AK105 treatment modifications and recommended toxicity management guidelines related to infusion reactions are shown in Table 2. In the event of multiple concurrent low-grade AEs (permanent discontinuation of AK105 will not be required when occurring alone), AK105 will be discontinued permanently at the discretion of the investigator.

Table 2 Treatment modification for infusion-related reactions caused by AK105

NCI-CTCAE Grade	Treatment Modification for AK105
**Grade 1-Mild**<br>• Minor, transient reaction; infusion interruption not required; and intervention not required.	• Reduce the AK105 infusion rate by 50% and closely monitor for any worsening.<br>• The total infusion time of AK105 should not exceed 120 min.
**Grade 2-Moderate**<br>Treatment or infusion interruption required and respond promptly to symptomatic treatment (e.g. antihistamine, NSAID, glucocorticoids, infusion therapy); prophylactic medications for < 24 hours.	• Interruption of AK105 infusion.<br>• When the infusion-related reaction has resolved or decreased in severity to at least Grade 1, resume the infusion at 50% of the previous infusion rate and closely monitor for any worsening.<br>• If a subject experiences a second infusion reaction of ≥ Grade 2 at a slower infusion rate, the infusion should be discontinued and the subject must withdraw from AK105 treatment.
**Grade 3 or 4-Serious or Life-threatening**<br>• Grade 3: Delayed symptom relief (e.g. failure to respond rapidly to symptomatic treatment and/or infusion interruptions); recurrence after symptom improvement; hospitalization is required for sequelae.<br>• Grade 4: Life-threatening; urgent treatment	• Stop the AK105 infusion immediately and disconnect infusion tube from the subject.<br>• Subjects must withdraw from AK105 treatment immediately and must not receive any further AK105 treatment.

required.	

Abbreviations: IV = intravenously; NCI-CTCAE = National Cancer Institute Common Terminology Criteria for Adverse Events; NSAID = non-steroidal anti-inflammatory drug.

3.1.3.2. Immune-related adverse event

An irAE is defined as an AE which is associated with drug exposure and consistent with an immune-mediated mechanism of action, with no clear alternative etiology. Based on the mechanism of action of AK105 targeting PD-1 to cause T cell activation and proliferation, it is possible to observe irAEs during this study. Potential irAEs, including immune-mediated colitis, dermatitis, pneumonia, hepatitis, encephalitis, nephritis, and endocrine disorders, may be similar to those with anti-PD-1/L1 drugs. Subjects should be monitored for signs and symptoms of irAEs. In the absence of an alternative etiology (e.g., infection or PD), an immune-related etiology associated with clinical signs or symptoms of colitis, dermatitis, pneumonia, hepatitis, encephalitis, nephritis, and endocrine disorders should be considered. Guidelines for the diagnostic evaluation of immune-related adverse events are detailed in Appendix 5.

Treatment-emergent AEs are graded according to NCI CTCAE 4.03, and treatment of AK105 is adjusted based on grade to manage potential irAEs.

- For Grade 1 irAE, no dose modification is needed.
- For Grade 2 irAE, treatment with AK105 will be discontinued until Grade 2 resolves to ≤ Grade 1:
  1) If toxicity worsens, treat as Grade 3 or 4.
  2) If toxicity improves to baseline, treatment should be performed at the next scheduled treatment visit.
  3) If a Grade 2 irAE does not resolve to ≤ Grade 1 or baseline within 8 weeks, AK105 will be permanently discontinued or decision will be made after communication between the investigator and the sponsor.
- In the event of a Grade 3 irAE, AK105 treatment may be permanently discontinued based on individual toxicity.
- In the event of a Grade 4 irAE, AK105 treatment must be permanently discontinued.
- The maximum interval allowed for drug interruption is 8 weeks. If AK105 can not be resumed within 8 weeks, the subject will permanently discontinue AK105 and enter the follow-up phase. The following two conditions are excluded: glucocorticoids for irAEs, AK105 dose interruption for more than 8 weeks due to the glucocorticoid dose reduction, or AK105 interruption for more than 8 weeks due to treatment of AEs that are possibly unrelated or not related to AK105. In these cases, discussion with the

medical monitor is required to decide whether AK105 treatment can be continued.

In general, it is recommended to manage irAEs according to the following guidelines. Subjects should be fully evaluated to exclude any alternative etiology (e.g., disease progression, concomitant medications and infection, etc.). Serologic, immunologic, and histological (biopsy) data should be used to support the diagnosis of irAEs, as appropriate.

- In the absence of a clear alternative etiology, all events should be considered possibly immune-related.
- Symptomatic and topical treatment for low-grade (Grade 1 or 2, unless otherwise specified) events should be considered.
- Treatment with systemic glucocorticoids (e.g, prednisone or intravenous equivalent drugs) for persistent low-grade or serious (≥ Grade 3) events should be considered.
- If symptoms recur or worsen during the gradual reduction of glucocorticoids, increase the dose of glucocorticoids until symptoms stabilize or improve and then the gradual reduction of glucocorticoids will be given again at a slower rate.
- For events that do not respond to systemic glucocorticoids, after discussion with the medical monitor, consideration should be given to the use of more potent immunosuppressants, i.e. TNF antagonists (e.g., infliximab, and mycophenolate mofetil).
- Discontinuation of study drug is not mandatory for Grade 3 or 4 inflammatory reactions (e.g., inflammatory response in metastatic disease sites, and lymph nodes) due to local tumor response.
- Consultations with physicians in other areas (e.g. cardiology or autoimmune disease specialties) should be scheduled in a timely manner to make appropriate treatment decisions.
- In the event of multiple concurrent low-grade AEs (discontinuation will not be required when occurring alone), AK105 will be discontinued at the discretion of the investigator.
- After discontinuation of study drug due to a Grade 3 or 4 event, AK105 may resume after discussion with the medical monitor if the subject rapidly responds well and the toxicity resolves rapidly.

AK105 treatment modification and toxicity management guidelines for immune-related AEs are provided in Table 3.

Table 3 AK105 treatment modification and recommended toxicity management guidelines for immune-related adverse events

**Gastrointestinal irAEs**<br>(Non-inflammatory causes should be excluded. If a non-inflammatory cause is determined, treat accordingly and continue treatment with AK105. Opioids/anesthetics may mask the symptoms of perforation. Infliximab should not be used in cases of perforation or sepsis.)		
**Severity of Diarrhoea/Colitis**	**Treatment**	**Follow-up**

(NCI-CTCAE v4.03)		
**Grade 1**<br>Diarrhoea: Increase of < 4 stools per day over baseline; mild increase in ostomy output compared to baseline<br>Colitis: Asymptomatic; clinical findings or diagnosis only, no treatment is required	• Continue AK105 treatment<br>• Symptomatic treatment (eg, loperamide)	• Closely monitor for worsening symptoms<br>• Instruct subjects to report immediately if worsening occurs<br>If worsens:<br>• Treat as Grade 2 or 3-4
**Grade 2**<br>Diarrhoea: Increase in stool frequency 4-6 times per day, IV fluids replacement less than 24 hours, and moderate increase in ostomy output compared with baseline.<br>Colitis: Abdominal pain; mucous or bloody stool	• Delay AK105 treatment<br>• Symptomatic treatment	If improves to Grade 1:<br>• Resume treatment with AK105 (the dose of glucocorticoids shall be reduced before resumeing if glucocorticoids have been given)<br>• If persists > 5 to 7 days or recurrences:<br>• Methylprednisolone 0.5-1.0 mg/kg/day or equivalent drugs<br>• When symptoms improve to Grade 1, gradually reduce the dose of glucocorticoids for at least 1 month. Consider prophylactic antibiotics for opportunistic infection, and then resume AK105 as per protocol.<br>If symptoms worsen or persist for > 3-5 days after oral glucocorticoids:<br>• Treat as Grade 3 to 4
**Grade 3 to 4**<br>Diarrhea (Grade 3): Increase in stool frequency ≥ 7 times per day; fecal incontinence; hospitalization required; severe increase in ostomy output; affecting personal activities of daily living compared with baseline<br>Colitis (Grade 3): Severe abdominal pain; change in bowel habits; medical treatment required; positive peritoneal irritation sign<br>Grade 4:<br>Life-threatening, life-threatening perforation; urgent treatment indicated	• Grade 3: Delay AK105 treatment<br>Grade 4: Discontinue AK105 permanently<br>• Methylprednisolone 1.0 to 2.0 mg/kg/day IV or equivalent drugs<br>• Add prophylactic antibiotics to prevent opportunistic infections<br>• Consider lower abdominal endoscopy	If improves:<br>• Continue glucocorticoid therapy until symptoms resolve to Grade 1 and then gradually reduce the dose at least 1 month<br>If persists > 3-5 days, or recurrences after improvement:<br>• Add infliximab 5 mg/kg (if no contraindications), note: infliximab should not be used in cases of perforation or sepsis.
**Skin irAEs**		

(Exclude other causes of skin problems, such as infections, effects of other drugs, skin symptoms caused by other diseases or dermatosis, etc., and if non-inflammatory causes are determined, treat accordingly and continue AK105 treatment)		
**Rash Grade**<br>(NCI-CTCAE v4.03)	**Treatment**	**Follow-up**
**Grade 1 to 2**<br>Covering ≤ 30% of body surface area	• Symptomatic treatment (eg, antihistamines, topical glucocorticoids)<br>• Continue AK105 treatment	<u>If persists > 1-2 weeks or recurrences:</u><br>• Consider skin biopsy<br>• Delay AK105 treatment<br>• Consider methylprednisolone 0.5-1.0 mg/kg/day IV or equivalent drugs. If symptoms improve, gradually reduce the dose of glucocorticoids for at least 1 month. Consider prophylactic antibiotics for opportunistic infection, and resume AK105<br><u>If worsens:</u><br>• Treat as Grade 3 to 4
Grade 3 to 4<br>Covering > 30% of body surface area;	• Grade 3: Delay AK105 treatment<br>Grade 4: Discontinue AK105 permanently<br>• Consider skin biopsy<br>• Dermatology consultation<br>• Methylprednisolone 1.0 to 2.0 mg/kg/day IV or equivalent drugs	If Grade 3 rash does not improve to ≤ Grade 1 or baseline within 30 days after a temporary delay in AK105, then<br>• discontinue AK105 permanently.<br><u>If improves to Grade 1:</u><br>• Gradually reduce the dose of glucocorticoids at least 1 month and increase prophylactic antibiotics to prevent opportunistic infections<br>• Resume AK105 (for Grade 3 events that have improved to Grade 1)
**Lung irAEs**<br>(Non-inflammatory causes should be excluded. If a non-inflammatory cause is determined, treat accordingly and continue treatment with AK105. Evaluation is performed by imaging and lung condition consultation.)		
**Pneumonia Grade**<br>(NCI-CTCAE v4.03)	**Treatment**	**Follow-up**
Grade 1<br>Asymptomatic; clinical examination or diagnostic findings only; intervention not required	• Continue AK105 treatment<br>• Monitor symptoms every 2-3 days<br>• Consider respiratory and infectious disease specialist consultation	• Reimaging at least every 3 weeks<br><u>If worsens:</u><br>• Treat as Grade 2 or 3-4
Grade 2<br>Symptomatic; intervention indicated; affecting instrumental activities of daily living	• Delay AK105 treatment<br>• Respiratory and infectious disease specialist consultation<br>• Monitor symptoms daily, consider hospitalization<br>• Methylprednisolone 1.0 mg/kg/day IV or equivalent drugs<br>• Consider bronchoscopy, lung biopsy	• Reimaging every 1 to 3 days<br><u>If improves:</u><br>• When symptoms return to near baseline, gradually reduce the dose of glucocorticoids for at least 1 month, then resume AK105 treatment, and consider prophylactic antibiotics<br><u>If not improving after 2 weeks or worsening:</u><br>• Treat as Grade 3 to 4

Grade 3 to 4<br>Severe symptoms; limited personal independence; oxygen required;<br>life-threatening respiratory disorder; urgent treatment (tracheotomy or intubation) indicated	• Discontinue AK105 permanently<br>• Hospitalization<br>• Respiratory and infectious disease specialist consultation<br>• Methylprednisolone 2 to 4 mg/kg/day IV or equivalent drugs<br>• Add prophylactic antibiotics to prevent opportunistic infections<br>• Consider bronchoscopy, lung biopsy	If symptoms improve to baseline:<br>• Gradually reduce the dose of glucocorticoids for at least 6 weeks<br>If not improving or worsening after 48 h:<br>• Add additional immunosuppression (e.g., infliximab, cyclophosphamide, IVIG, or mycophenolate mofetil).
**Liver irAEs**<br>(Non-inflammatory causes should be excluded. If a non-inflammatory cause is determined, treat accordingly and continue treatment with AK105. Consider imaging for obstruction)		
**Grade of Increase in Liver Test Parameters**<br>(NCI-CTCAE v4.03)	**Treatment**	**Follow-up**
Grade 1<br>Grade 1 AST or ALT > ULN to 3.0 × ULN and/or TBil > ULN to 1.5 × ULN	• Continue AK105 treatment	• Continue monitoring of liver function<br>If worsens:<br>• Treat as Grade 2 or 3-4
Grade 2<br>AST or ALT > 3.0 to ≤ 5 × ULN and/or TBil > 1.5 to ≤ 3 × ULN	• Delay AK105 treatment<br>• Increase frequency of monitoring to every 3 days	If returns to baseline:<br>• Resume routine monitoring and AK105 treatment<br>If the increase persists for > 5 to 7 days or worsens:<br>• Methylprednisolone 0.5 to 1 mg/kg/day or equivalent drugs, gradually reduce the dose of glucocorticoids for at least 1 month when LFTs return to Grade 1 or baseline, consider prophylactic antibiotics to prevent opportunistic infections, and resume AK105 treatment
Grade 3 to 4<br>AST or ALT > 5 x ULN and/or TBil > 3 x ULN	• Permanently discontinue AK105 (AK105 may be delayed if AST/ALT ≤ 8 × ULN and total bilirubin ≤ 5 × ULN)<br>• Increase monitoring frequency to once every 1-2 days<br>• Methylprednisolone (1.0 to 2.0 mg/kg/day, IV) or equivalent (in the presence of Grade 4 hepatitis, the recommended starting dose is 2.0 mg/kg/day IV methylprednisolone or equivalent)<br>• Add prophylactic antibiotics to prevent opportunistic infections<br>• Gastroenterologist consultation<br>• Consider magnetic resonance imaging (MRI)/computed tomography (CT) scan of liver or	If returns to Grade 2:<br>• Gradually reduce the dose of glucocorticoids for at least 1 month<br>If does not improve, worsen, or rebound within > 3 to 5 days:<br>• Add 1 g of mycophenolate mofetil, BID<br>• If no response within an additional 3-5 days, consider other immunosuppressants according to local guidelines.

	liver biopsy, if clinically warranted	
**Renal irAEs**<br>(Non-inflammatory causes should be excluded. If a non-inflammatory cause is determined, treat accordingly and continue with AK105)		
**Grade of Creatinine Elevation**<br>(NCI-CTCAE v4.03)	**Treatment**	**Follow-up**
Grade 1<br>Creatinine > ULN and > baseline, but ≤ 1.5 × baseline	• Continue AK105 treatment<br>• Monitor creatinine levels weekly	If returns to baseline:<br>• Resume routine creatinine monitoring per protocol<br>If worsens:<br>• Treat as Grade 2-3 or 4
**Grade 2 to 3**<br>Creatinine > 1.5 × baseline and ≤ 6 × ULN	• Delay AK105 treatment<br>• Monitor creatinine levels every 2 to 3 days<br>• Consider methylprednisolone 0.5 to 1.0 mg/kg/day IV or equivalent drugs<br>• Consider renal biopsy	If returns to Grade 1:<br>Gradually reduce the dose of glucocorticoids within at least 1 month. Consider prophylactic antibiotics to prevent opportunistic infections, resume AK105 as per protocol and start routine creatinine monitoring if the increase persists for > 7 days or worsens:<br>• Treat as Grade 4
**Grade 4**<br>Creatinine > 6 × ULN	• Discontinue AK105 permanently<br>• Monitor creatinine levels once daily<br>• Methylprednisolone 1.0 to 2.0 mg/kg/day IV or equivalent drugs<br>• Nephrology consultation<br>• Consider renal biopsy	If returns to Grade 1:<br>• Gradually reduce the dose of glucocorticoids at least 1 month and increase prophylactic antibiotics to prevent opportunistic infections
**Neurological irAEs**<br>(Non-inflammatory causes should be excluded. If a non-inflammatory cause is determined, treat accordingly and continue with AK105)		
**Neurotoxicity Grade**<br>(NCI-CTCAE v4.03)	**Treatment**	**Follow-up**
Grade 1<br>Asymptomatic or mild symptoms; no therapeutic intervention required	• Continue AK105 treatment	• Continuous monitoring of patients<br>If worsens:<br>• Treat as Grade 2 or 3-4
Grade 2<br>Moderate symptoms; affecting instrumental activities of daily living	• Delay AK105 treatment<br>• Treat symptoms according to local guidelines<br>• Consider methylprednisolone 0.5 to 1.0 mg/kg/day IV or equivalent drugs	If symptoms improve to baseline:<br>• Resume AK105 treatment<br><br>If worsens:<br>• Treat as Grade 3 to 4
Grade 3 to 4<br>Serious symptoms; limiting self-care; life-threatening; urgent	• Discontinue AK105 permanently<br>• Neurology consultation<br>• Treat symptoms according to	If improving to Grade 2:<br>• Gradually reduce the dose of glucocorticoids for at least 1 month

intervention indicated	local guidelines<br>• Consider methylprednisolone 1.0 to 2.0 mg/kg/day IV or equivalent drugs<br>• Add prophylactic antibiotics to prevent opportunistic infections	If symptoms worsen or atypical manifestations occur:<br>• Consider IVIG or other immunosuppressive therapy according to local guidelines
**Endocrine irAEs**<br>(Non-inflammatory causes should be excluded. If a non-inflammatory cause is determined, treat accordingly and continue treatment with AK105. Consider visual field testing, endocrinology consultation, and imaging.)		
**Endocrine Toxicity Grade**<br>(NCI-CTCAE v4.03)	**Treatment**	**Follow-up**
Grade 1<br>(Definition of CTCAE Grade 1 per NCI CTCAE v4.03, depending on the type of endocrinopathy)	No dose modification	For Grade 1 (including asymptomatic patients with TSH elevation):<br>• Perform appropriate endocrine function tests to monitor subjects<br>• If TSH < 0.5 × LLN or TSH > 2 × ULN, or consistently out of range in 2 subsequent measurements, add free T4 levels in subsequent cycles as clinically indicated and consider endocrine consultation
Grade 2	Discontinue study drug/regimen until the event resolves to ≤ Grade 1<br>• If toxicity worsens, treat as Grade 3 or 4<br>• If toxicity improves to baseline, treatment should be performed at the next scheduled treatment date	For Grade 2 (including patients with symptomatic endocrine disease):<br>• Initiate hormone replacement therapy as needed<br>• Evaluate endocrine function and consider pituitary scan as clinically indicated<br>• Short-term high-dose glucocorticoid therapy (e.g. methylprednisolone 1 to 2 mg/kg/day or equivalent drugs by intravenous injection) and relevant hormone replacement therapy (e.g. levothyroxine, hydrocortisone, or sex hormones) could be applied for subjects with endocrine dysfunction<br>• If symptoms improve, gradually reduce the dose of glucocorticoids for ≥ 4 weeks and use prophylactic antibiotics to prevent opportunistic infections<br>• Repeat laboratory/MRI is performed as clinically indicated for subjects with normal endocrine function (laboratory or MRI scan)
**Grade 3**	• Discontinue study drug/regimen until endocrine disease symptoms are controlled<br>• Resume study drug/regimen if symptoms are controlled after next scheduled dose	• Initiate IV glucocorticoid therapy (e.g. methylprednisolone IV or equivalent drugs) at 1 to 2 mg/kg/day<br>• Replacement therapy will be performed in the event of isolated hypothyroidism without interruption of

		treatment or use of glucocorticoid<br>• Hormone replacement therapy is used if necessary<br>• For adrenal crisis, severe dehydration, hypotension, or shock: Immediately initiate IV glucocorticoids with mineralocorticoid activity<br>• Endocrinology consultation<br>• If symptoms improve, gradually reduce the dose of glucocorticoids for ≥ 4 weeks and use prophylactic antibiotics to prevent opportunistic infections
Grade 4	Discontinue study drug/regimen permanently	Same treatment as above for Grade 3 events

If sustained clinical improvement is observed, patients receiving IV glucocorticoids may switch to an equivalent dose of oral glucocorticoids (e.g., prednisone) at the start of dose reduction or earlier. Low bioavailability of oral glucocorticoids should be considered when switching to equivalent oral glucocorticoids.

ALT = alanine aminotransferase; AST = aspartate aminotransferase; CT = computed tomography; irAE = immune-related adverse event; IV = intravenously; IVIG = intravenous immunoglobulin; LFT = liver function test; LLN = lower limit of normal; MRI = magnetic resonance imaging; NCI-CTCAE = National Cancer Institute Common Terminology Criteria for Adverse Events; T4 = thyroxine; TSH = thyroid stimulating hormone; ULN = upper limit of normal.

If there are inconsistencies in toxicity management guidelines or clinical routine associated with other immune checkpoint inhibitors, please communicate with the sponsor and deal with them.

3.1.3.3. Non-immune-related Adverse Events

For subjects with grade 3 or higher adverse events associated with AK105 that do not meet the criteria for treatment discontinuation, AK105 may be withheld and no missing dose may be administered thereafter. Dose interruption is not required for AEs that are clearly unrelated to AK105 or laboratory abnormalities that are not clinically significant, and dose reduction is not permitted. All toxicities will be graded according to NCI CTCAE 4.03.

Table 4 AK105 treatment modification and recommended toxicity management guidelines for non-immune-related reactions

Event Grade	Dose Modifications and Toxicity Management
Any grade	• Note: Dose modifications are not required for AEs not related to study treatment (i.e., events due to underlying disease) or clinically insignificant laboratory abnormalities.
Grade 1	• No dose modification<br>• Treatment will be performed according to the institutional standards
Grade 2	• Study drug will be suspended until the event resolves to ≤ Grade 1 or baseline<br>• Treatment will be performed according to the institutional standards
Grade 3	• Study drug will be suspended until the event resolves to ≤ Grade 1 or baseline<br>• For AEs that decrease to ≤ Grade 2 within 7 days or resolve to ≤ Grade 1 or baseline within 14 days, study drug should be resumed at the next scheduled dose. Otherwise, study drug should be discontinued permanently.<br>• Treatment will be performed according to the institutional standards.
Grade 4	• Study drug will be discontinued permanently at the investigator's clinical discretion and in consultation with the medical monitor (Note: for Grade 4 laboratory events, a decision to discontinue treatment permanently will be made based on concomitant clinical signs/symptoms).<br>• If a subject's toxicity resolves rapidly after treatment and the tumor has significantly resolved previously, AK105 treatment may be resumed and study treatment may continue after discussion with the medical monitor.<br>• Treatment will be performed according to the institutional standards

If there are inconsistencies in toxicity management guidelines or routine clinical procedures related to other immune checkpoint inhibitors in the above principles, the investigator will deal with them after communication with the sponsor.

3.1.4. Duration of Study

The study will be completed at least 12 months after the first dose of the last subject, and data analysis is performed. If there are still subjects receiving study drug at that time, they may, after obtaining ICFs, be transferred to an extension study to continue treatment with this study drug until, in the judgment of the investigator, the subject is unable to continue to benefit, the disease progresses, intolerable toxicity occurs, the investigator decides, the subject withdraws the ICF, or the subject dies.

3.1.5. Duration of Subject Participation

The number of treatment cycles is not fixed. Subjects who achieve complete response (CR), partial response (PR), or stable disease (SD) will receive continuous treatment without interruption in the absence of withdrawal of informed consent, definite progression, or unacceptable toxicity.

3.1.6. Study Discontinuation Criteria

The sponsor may decide to terminate the study in accordance with the actual circumstances of clinical development.

3.2. Study Design Rationale

3.2.1. Rationale for Single-arm Studies

Platinum-containing chemotherapy is generally considered the standard of care for patients with recurrent or metastatic nasopharyngeal carcinoma. For patients who have failed first-line platinum-based chemotherapy, subsequent second-line therapy depends on patient performance status, toxicity, and time interval to recurrence after a platinum-based regimen. Re-treatment with platinum-based combination chemotherapy may be considered for patients who have failed first-line platinum-based chemotherapy but have no disease progression for more than 1 year. Second-line chemotherapy, including gemcitabine, capecitabine, or paclitaxel, may be considered for patients who have failed first-line platinum-based chemotherapy and whose disease has recurrent within one year of chemotherapy. However, to date, there is no recognized standard third-line systemic therapy. Treatment with pembrolizumab achieved an ORR of 25.9% in patients with recurrent/metastatic PD-L1-positive NPC, with a median duration of response of 17.1 months (**Hsu C, 2017**). Treatment with nivolumab achieved an ORR of 20.5% in patients with recurrent/metastatic NPC. In the subgroup analysis, ORR of patients in the PD-L1 positive group was 33%, and that of patients in the PD-L1 negative group was 13% (**Ma BBY, 2018**). In 2017, the Chinese Society of Clinical Oncology (CSCO 2017) announced that the ORR of patients with recurrent/metastatic NPC treated with anti-PD-1 monoclonal antibody JS001 was 30.8%. In the subgroup analysis, the ORR of patients in the PD-L1 positive group was 42.1%, and that of patients in the PD-L1 negative group was 20% (CSCO 2017). The above data indicated that anti-PD-1 monoclonal antibody monotherapy had significant therapeutic effect on platinum-resistant recurrent/metastatic NPC.

As patients with recurrent/metastatic NPC have failed to improve after platinum-containing chemotherapy and one or more other chemotherapy, or have failed to improve after platinum-containing chemotherapy and are intolerant to other available chemotherapy, and there is no recognized standard or effective recommended systemic therapy, therefore, this study chose a single-arm design, with ORR as primary endpoint and duration of response as key secondary endpoint. A key feature of immunotherapy is that once a patient has relieved, the response is sustained and durable, which can be expected to reflect clinical benefit.

3.2.2. Rationale for Selection of 200 mg Q2W as Treatment Regimen

In this study, 200 mg AK105 Q2W is proposed to be administered. This mode of administration was based on the safety of Phase Ia dose escalation trial (AK105-101) conducted in Australia, preliminary anti-tumor activity (see Section 1.1.5), and exposure (concentration)-response (PD-1 receptor occupancy) relationship data, as well as preclinical in vitro/in vivo pharmacodynamics and drug-related comparison data of similar drugs. In

pharmacodynamic studies, PD-1 receptor occupancy showed that AK105 rapidly (24 h) saturated occupying peripheral PD-1 at a dose of 3 mg/kg Q2W, and the PD-1 receptor occupancy was up to 85%-95% at 15-57 days after administration.

3.2.3. Rationale for Selection of 24-week Follow-up after the First Dose for the Analysis of the Primary Endpoint

The ORR of nivolumab monotherapy in patients with recurrent/metastatic NPC was 20.5% (9/44), with 7 patients achieving objective response at Week 8 of the first tumor evaluation and 2 patients achieving objective response at Week 16 of the second tumor evaluation. The ORR of pembrolizumab monotherapy in patients with recurrent/metastatic NPC was 25.9% (7/27), with 6 (86%) patients achieving objective response at Week 8 of the first tumor evaluation. In this study, the primary endpoint is analyzed after the last subject is treated for at least 24 weeks (This follow-up assessment is not required if the subject has reached radiographic disease progression by 24 week). It is expected that most objective response events will be observed.

3.2.4. Rationale for Treatment after Disease Progression

Responses to immunotherapy may differ from typical responses observed with cytotoxic chemotherapy (**Wolchok JD, 2009**), including clinical objective responses and/or stable disease that may occur after disease progression, and new lesions that do not represent disease progression with immunotherapy, from which clinical benefit is still possible. There are two possible reasons for this phenomenon. First, worsened inflammation within the tumor may lead to an increase in tumor volume, manifested by measurable lesion enlargement and the appearance of new, visible, unmeasurable lesions. Over time, the malignant and inflammatory portions of the mass may shrink, leading to a marked imaging response and improvement in clinical signs. Second, in some patients, the anti-tumor immune response is initiated slowly, and the inhibitory effect on tumor growth is lower than that of tumor growth kinetics in the early stage. Over time, anti-tumor activity will predominate and manifest as an improvement in imaging response and clinical signs. In a Phase 2 study, a subgroup analysis is performed for patients who continued treatment with nivolumab beyond disease progression (**Escudier B, 2017**). Among 142 advanced RCC patients who continued treatment with nivolumab after disease progression and with pre- and post-progression tumor measurements, 13% of patients had a subsequent reduction in tumor burden of ≥ 30%.

4. STUDY POPULATION

4.1. Enrollment

Relevant information about this study will be provided to each subject and all questions raised will be answered until the subject is satisfied and the ICF will be signed and dated. These will be completed before any study-specific procedures are performed. Further information on the informed consent procedure is provided in Section 13.4. Once the investigator or designee obtains written informed consent from a subject and determines that all inclusion and exclusion criteria are met, the subject will be considered enrolled in the study. For subjects receiving study treatment, data obtained at all study visits will be recorded on the case report form (CRF). For subjects who meet the inclusion criteria, do not meet the exclusion criteria and/or do not receive study drug, only a small number of data (demographics, reason for withdrawal, and AEs) will be recorded on the CRF. Once a subject fails screening or has decided to withdraw from the study prior to receiving study drug, no additional data will be collected for that subject.

4.1.1. Inclusion Criteria

Subjects must meet all of the following inclusion criteria to be enrolled in the study:

1) Signing of written ICFs.
    - Subjects must sign and date an IRB/IEC-approved ICF in accordance with the guidelines of the competent authority and study facility. ICF must be signed prior to the conduct of any protocol-related procedures (not part of the subject's routine medical care).
    - Subjects must be willing and able to comply with the scheduled visits, treatment regimens, laboratory tests, and other requirements in the study.
2) Males or females aged ≥ 18 to ≤ 75 years at enrollment.
3) Eastern Cooperative Oncology Group (ECOG) performance status score of 0 or 1.
4) Expected survival ≥ 3 months.
5) Histopathologically confirmed nonkeratinizing differentiated or undifferentiated nasopharyngeal carcinoma.
6) Patients with stage IVb [Chinese Nasopharyngeal Carcinoma Staging 2017 (2008 Expert Consensus on Nasopharyngeal Cancer Staging Revision)] metastatic nasopharyngeal carcinoma at enrollment, who are not eligible for radical local therapy.
7) Failure of prior first-line platinum-containing chemotherapy (monotherapy or concomitant medication) and second-line monotherapy or concomitant medication. Treatment failure is defined as disease progression occurring during or after chemotherapy. For subjects who have received prior neoadjuvant chemotherapy, concurrent chemoradiotherapy, or adjuvant chemotherapy, the original treatment regimen should be the first-line regimen if recurrence/metastasis occurs within 6 months after the end of the last treatment. All treatment changes due to drug

intolerance are not considered as treatment failure.

8) At least one measurable lesion (per RECIST v1.1), and the lesion can be considered a target lesion if it has been treated with radiotherapy, has unequivocally progressed and is measurable as judged by radiology.

9) Subjects agree to provide previously archived tumor tissue samples (tissue samples collected within 3 years prior to enrollment) or undergo biopsies to collect tumor lesion tissue (at least 3 unstained FFPE pathological sections. If the central laboratory determines that the samples are insufficient for PD-L1 IHC testing, 3 additional unstained FFPE pathological sections are required) for PD-L1 immunohistochemistry (IHC) testing by the central laboratory (preferably tumor tissue samples obtained recently). Tumor lesions planned for biopsy should not be used as target lesions for disease assessment unless there are no other lesions suitable for biopsy. If there is no archival tumor tissue samples within 3 years, and biopsy may increase the subject's risk as judged by the investigator, the archival tumor tissue sample beyond 3 years may be collected after discussion with the medical monitor.

10) Adequate organ function is determined by the following requirements (supportive treatment with any blood components and cell growth factors is not allowed within 2 weeks prior to starting study treatment):

a) Hematology:

i. Absolute neutrophil count (ANC) ≥ 1.5 × 10^9/L (1,500/mm^3);

ii. Platelet count ≥ 100 × 10^9 /L (100,000/mm^3);

iii. Hemoglobin ≥ 9.0 g/dL.

b) Kidney:

i. Serum creatinine ≤ 1.5 × ULN or calculated creatinine clearance* (CrCl) ≥ 50 mL/min

* CrCl will be calculated using the Cockcroft-Gault formula (**Cockcroft DW, 1976**)

CrCL (mL/min) = {(140 - age) × body weight (kg) × F}/(SCr (mg/dL) × 72)

where F = 1 for men and 0.85 for women; SCr = serum creatinine.

c) Liver:

i. Serum total bilirubin (TBil) ≤ 1.5× ULN; TBil ≤ 3× ULN for subjects with liver metastases or documented/suspected Gilbert's disease

ii. AST and ALT ≤ 2.5 × ULN; for subjects with liver metastasis, AST and ALT ≤ 5 × ULN

d) Coagulation

i. International normalized ratio (INR) and activated partial thromboplastin time (APTT) ≤1.5× ULN (unless participant is receiving anticoagulant therapy, and coagulation parameters (PT/INR and APTT) are within the expected range of anticoagulant treatment at screening).

11) Female subjects of childbearing potential must have a negative serum pregnancy test (if serum pregnancy test is not available, urine pregnancy test may be adopted) within 3 days prior to the first dose. If a female subject of childbearing potential has sexual intercourse with a nonsterile male partner, the subject must use 2 acceptable effective methods of contraception (see Table 5) from screening and must agree to continue these precautions until 120 days after the last dose of study drug; periodic abstinence, safe-term contraception, and extracorporeal ejaculation are unacceptable contraceptive methods.

    a) Females of childbearing potential are those who have not been surgically sterile (i.e., bilateral tubal ligation, bilateral oophorectomy, or complete hysterectomy) or who are not postmenopausal (menopause is defined as menolipsis for at least 12 consecutive months without alternative medical reasons and serum follicle-stimulating hormone levels within the laboratory reference range for postmenopausal women).

    b) Highly effective methods of contraception are defined as those that result in a low failure rate (i.e. less than 1% per year) when used consistently and correctly. Acceptable effective methods of contraception are shown in Table 5. Not all methods of contraception are highly effective. In addition to barrier contraception (e.g., male condom plus spermicide), female subjects of childbearing potential must also use hormonal contraception (e.g., contraceptives) alone to ensure that pregnancy does not occur.

12) If a nonsterile male subject has sexual intercourse with a female partner of childbearing potential, the subject must use an effective contraception method (see Table 5) from the start of screening until Day 120 after the last dose of study drug. Childbearing potential female partners of male subjects should also use effective methods of contraception throughout the study.

Table 5 Effective methods of contraception (2 methods must be used)

Barrier contraception	Hormonal contraception
• Male condom + spermicide<br>• Copper T-type intrauterine device<br>• T-type intrauterine device containing progesterone<br>• Levonorgestrel-releasing intrauterine device (e.g., mirena)	• Implant<br>• Hormonal contraceptive injection<br>• Compound contraceptives<br>• Low-dose oral contraceptive pills<br>• Contraceptive patch

4.1.2. Exclusion Criteria

Subjects who meet any of the following criteria are not eligible to participate in this study:

1) Subjects who have participated in the study of an investigational drug, received study treatment or used investigational device within 4 weeks prior to the first dose of AK105.

2) Subjects who have enrolled in another clinical study also, unless it is an observational (non-interventional) clinical study or a follow-up period for an

interventional study (defined as the first dose of study drug in the current study is at least 4 weeks after the last dose of study drug or after at least 5 half-lives of the study drug in the previous clinical study, whichever occurs later).

3) Subjects who have received the last radiotherapy or anti-tumor therapy (chemotherapy, targeted therapy, immunotherapy, traditional Chinese medicine with anti-tumor activity, or tumor embolization, etc.) within 4 weeks prior to the first dose of AK105. Subjects who have been treated with nitrosourea or mitomycin C within 6 weeks prior to the first dose of AK105. Local palliative radiotherapy for symptom control is allowed, but must be completed at least 2 weeks prior to the first dose of AK105, and no additional radiotherapy is planned for the same lesion.

4) Subjects who have previously received any anti-PD-1, anti-PD-L1, anti-CTLA-4 antibody, or any other antibody or drug therapy targeting T-cell costimulation or checkpoint pathway, e.g., ICOS or agonists (e.g., CD40, CD137, GITR, and OX40, etc.).

5) Subjects with other active malignancies within 2 years prior to enrollment. Except for locally curable cancers (which appear cured), such as basal or cutaneous squamous cell carcinoma, superficial bladder cancer, carcinoma in situ of the cervix or breast.

6) Subjects with active, known or suspected autoimmune disease, or with a history of autoimmune disease, with the following exceptions: vitiligo, alopecia, Graves' disease, psoriasis, or eczema that does not require systemic treatment within recent 2 years, hypothyroidism(due to autoimmune thyroiditis) that is asymptomatic or only requires a stable dose of hormone replacement therapy to achieve stable status , type I diabetes that only requires stable dose of insulin replacement therapy, or asthma that has resolved completely in childhood and requires no intervention in adulthood, or the diseases that will not recur in the absence of external triggers (see **Appendix 3** for listing of autoimmune diseases).

7) Subjects with active or previously documented inflammatory bowel disease (e.g., Crohn's disease, ulcerative colitis, or chronic diarrhea).

8) Subjects who require systemic treatment with corticosteroids (> 10 mg/day of prednisone or equivalent) or other immunosuppressive agents within 14 days prior to the first dose of study drug. With the following exceptions:

   a) Inhaled or topical steroids and adrenal corticosteroids with a dose >10 mg/day of prednisone equivalent are allowed in the absence of active autoimmune disease.

   b) Physiologic doses of systemic glucocorticoids do not exceed 10 mg/day prednisone, or equivalent doses of other glucocorticoids.

   c) Glucocorticoids are used as prophylaxis for hypersensitivity reactions (e.g., before CT examination).

9) Subjects who are positive for HIV.

10) Known history of primary immunodeficiency.

11) Known history of active pulmonary tuberculosis (TB). Subjects with suspected

active TB should be examined through tests of chest x-ray, sputum, clinical symptoms and signs to exclude this condition.

12) Known history of allogeneic organ transplantation and allogeneic hematopoietic stem cell transplantation.

13) Subjects with history of gastrointestinal perforation and/or fistula within 6 months prior to enrollment (surgically removed gastrointestinal perforation or fistula are allowed).

14) Subjects with necrotizing lesions found within 4 weeks prior to enrollment, and are at risk of major hemorrhage as judged by the investigator.

15) Known history of interstitial lung disease.

16) Serious infection within 4 weeks prior to first dose, including but not limited to complications requiring hospitalization, sepsis, or severe pneumonia.

17) Active infection requiring systemic treatment.

18) Patients with untreated chronic hepatitis B, or chronic hepatitis B virus (HBV) carriers with HBV DNA exceeding 500 IU/ml, or patients with active hepatitis C should be excluded. Inactive HBsAg carriers, patients with hepatitis B who have been treated and in stable condition (HBV DNA < 500 IU/mL), and patients with cured hepatitis C can be enrolled. Subjects who are positive for HCV antibody are eligible to participate in the study only if they are negative for HCV RNA.

19) Subjects who are scheduled for major surgeries within 30 days (decided by the investigator) after the first dose of AK105 or who have not fully recovered from previous surgery. Local surgery (e.g., placement of systemic ports, core needle biopsy, and prostate biopsy) is allowed provided that the surgery is completed at least 24 h before the first dose of study drug.

20) Subjects with known meningeal metastases, spinal cord compression, leptomeningeal disease, or active brain metastases prior to enrollment. Subjects with central nervous system (CNS) metastases who have been treated to be asymptomatic (e.g., without neurological dysfunction, epilepsy, or other typical symptoms and signs of central nervous system metastases) and who meet the following requirements prior to the first dose of AK105 may be included:

    a) No concurrent treatment (including but not limited to surgery, radiotherapy, and/or corticosteroids)

    b) After the last treatment, there is radiographic evidence of no progression of CNS metastasis at least 4 weeks prior to the first dose of AK105

    c) Systemic hormonal therapy has been discontinued for more than 2 weeks

21) Subjects with uncontrolled pleural effusion, pericardial effusion, or ascites requiring repeated drainage.

22) Subjects with uncontrolled intercurrent illness, including symptomatic congestive heart failure (New York Heart Association functional class III or IV in Appendix 4), uncontrolled hypertension, unstable angina, poorly controlled arrhythmias, acute or ongoing evidence of myocardial ischemia, severe active peptic ulcer disease or

gastritis, or mental illness/social condition that would limit the subject's compliance with the requirements of the study or affect the subject's ability to provide written informed consent. Any arterial thromboembolic event within 6 months prior to enrollment, including myocardial infarction, cerebrovascular accident, or transient ischemic attack; a history of deep vein thrombosis, pulmonary embolism, or any other serious thromboembolism.

23) Unresolved toxicity to prior anti-tumor therapy, which is defined as failure to recover to NCI CTCAE v4.03 Grade 0 or 1, or to the level specified in the inclusion/exclusion criteria, with the exception of alopecia. Subjects with irreversible toxicity that are not expected to worsen after study drug administration (e.g., hearing loss) may be included in the study after consultation with the medical monitor. Subjects with ≤ Grade 2 neuropathy are evaluated on a case-by-case basis after consultation with the medical monitor. Subjects with long-term toxicity due to radiotherapy that cannot be recovered at the discretion of the investigator may be included in the study after consultation with the medical monitor.

24) Subjects who are vaccinated with live or attenuated vaccines within 30 days prior to the first dose of AK105, or who plan to receive live or attenuated vaccines during the study.

25) Subjects with known history of severe hypersensitivity reactions to other monoclonal antibodies.

26) Subjects with known allergy to any component of the AK105 formulation.

27) Subjects with known history of psychotropic drug abuse, alcoholism, or drug addiction.

28) Pregnant or lactating women.

29) Any condition that, in the investigator, may result in a risk when receiving study drug, or would interfere with the study drug evaluation of the interpretation of subject safety or study results.

4.2. Subject Withdrawal from Treatment

All subjects may discontinue study treatment or withdraw from the study at any time. If a subject withdraws from study treatment or the study, the reason for withdrawal will be recorded in the CRF.

4.2.1. Reasons for Withdrawal

4.2.1.1. Withdrawal from Study Treatment

Reasons for withdrawal/discontinuation of study treatment:

- AEs (any AE that meets the criteria for permanent discontinuation of study drug as defined in Section 3.1.3, or that the investigator or sponsor considers to be contraindicated for further administration)
- Subject is lost to follow-up
- Subject dies

- Significant protocol deviations (e.g., non-compliance of subjects believed by the investigator or sponsor, justified withdrawal, pregnancy or planned pregnancy, requirements not met, etc.)
- Subject withdraws ICF
- Subject starts a new anti-cancer therapy
- Study termination by the sponsor
- Progressive disease (radiological progression); disease progression should be confirmed without significant clinical deterioration (see Section 3.1.2)
- Others (e.g., at the discretion of the investigator, clinical progression occurs)

For subjects who discontinue study treatment, please refer to Section 6.3 for the end of treatment and follow-up procedures. Subjects who discontinue study treatment due to an AE will be followed up by the investigator until the AE resolves or stabilizes.

4.2.1.2. Study Withdrawal

Withdrawal from the study includes discontinuation of all follow-up procedures. Reasons for withdrawal include:

- Subject dies
- Subject is lost to follow-up
- Subjects withdraw the ICF for participating in the study (including the follow-up period)
- Study termination by the sponsor

4.2.2. Withdrawal Procedure

Study treatment follow-up will end 90 days after the last dose of AK105. The procedures described in Section 6.3 will be performed at this visit.

4.2.3. Subject Re-screening Procedures

Re-screening (only once) will be allowed for subjects who failed to meet eligibility criteria at initial screening. Subject identification (SID) must remain the same at re-screening. The initial screening information and the reason why the subject is ineligible for the initial evaluation will be recorded on the Screening Log. For re-screened subjects, data from the initial screening evaluation will not be recorded in the clinical database.

5. STUDY DRUGS AND OTHER TREATMENTS

5.1. Study Drug

Akeso Tiancheng (Guangdong) Co., Ltd. will provide sufficient quantity of study drug to the investigator or provide them to the investigator through designated distribution center.

AK105 will be provided as a sterile, liquid solution. AK105 is an injection (solvent type) and the strength is 100 mg/10 mL/vial. Each glass vial contains 10 mL of study drug at a concentration of 10 mg/mL. Each vial of the drug product contains: AK105 monoclonal antibody (100 mg), sodium acetate (16.4 mg), sorbitol (450.0 mg), and polysorbate 80 (2 mg). Each vial is intended for single use only and should not be used to treat more than one subject.

AK105 should be used before the expiry date indicated on the package. AK105 should be stored at 2-8 °C and protected from light. Freezing and vigorous shaking are prohibited. AK105 does not contain any antimicrobial preservatives, therefore, care must be taken to ensure the sterility of the prepared solutions.

5.1.1. Methods of Assigning Subjects to Treatment Groups and Blinding

5.1.1.1. Subject Identification (SID)

Each subject will be assigned a unique SID. The SID number will be used to identify subjects throughout the study and will be entered in all study documents. The SID will consist of 5 digits, with the first 2 digits being the study site number and the last 3 digits being the sequential number assigned to the study site at screening. If a subject discontinues from the study at any time, the SID number may not be reassigned to another subject.

5.1.1.2. Randomized

Not applicable.

5.1.1.3. Blinding

The study is an open-label design and is not blinded. Subjects, study personnel, and sponsor will not be aware of the individual PD-L1 expression data of the subject and will be blinded to the individual PD-L1 expression data.

5.1.2. Preparation

This product is a sterile injection, colorless to pale yellow clear liquid. Careful examination is required before use to confirm that each vial of injection is free of damage, and that the solution in the vial is not coagulated, turbid or precipitated. If there are any defects in the investigational product, the investigator and the sponsor should be informed immediately. Refer to the Product Complaints section (Section 5.1.8) for further clarification.

For AK105 preparation, an intravenous bag containing 100 mL normal saline (0.9% (w/v) sodium chloride injection) is used. The study drug must be prepared using aseptic techniques.

First, the injection port of the infusion bag should be wiped and normal saline (20 mL) equal to the dose of AK105 drug product to be administered is withdrawn from the injection bag. Then, 20 mL of AK105 is added to the injection bag by injection. The bag should be gently inverted to thoroughly mix the solution. Do not shake the infusion bag. The final solution is visually inspected. If the infusion solution is not clear or the contents contain precipitation, the solution should be discarded (as described in Section 5.1.7) and recorded in the drug accountability log.

AK105 does not contain preservatives, and the solution containing AK105 must be used immediately after preparation. If the AK105 diluent cannot be used immediately and requires storage, the total storage time from opening the AK105 vial to the start of injection should not exceed 24 h in the freezer (2°C to 8°C) or 4 h at room temperature.

Prior to dosing, all prepared AK105 intravenous infusion bags must be labeled with the following: SID, subject initials, expiry date and time, and infusion personnel initials. Further instructions on the preparation and administration of the study drug will be provided to the site pharmacy in a manual.

5.1.3. Administration of Therapeutic Drugs

AK105 200 mg is administered by IV (Q2W, 4-week cycle) with an infusion time of 60 min ± 15 min. The infusion may be extended to a maximum of 120 min ± 15 min for subjects unable to tolerate the 60 min ± 15 min infusion.

After dilution in the IV bag, the entire contents of the IV bag should be administered by IV over 60 minutes using an in-line filter of 0.2 μm or 0.22 μm. After completion of the infusion, the IV line is flushed with the same volume of normal saline as the perfusion volume of the infusion device to ensure that full infusion is administered to the subject.

Since the compatibility of AK105 with other intravenous infusion drugs and solutions other than normal saline (0.9% (w/v) sodium chloride injection) is not known, AK105 solutions should not be infused using an IV line that infuses other solutions or drugs. The date, start time, interruption time, and completion time of AK105 administration must be recorded in the source document.

5.1.4. Monitoring of Dose Administration

Subjects will be monitored during and after the completion of infusion, and vital signs will be assessed within 30 min before the start of infusion, 30 min (± 10 min) during the 1-h infusion period, at the end of infusion (+ 10 min), 30 min (± 10 min) and 60 min (± 10 min) after the end of infusion. On Day 1 of Cycle 1, additional vital sign measurements will be performed 3 h (± 15 min) after the first infusion. For subsequent dosing, a 3-h observation period is not required unless the subject experiences an infusion-related reaction. If a subject experiences an infusion-related reaction, additional vital sign measurements will be performed 3 h after infusion and 3 h after each subsequent infusion. Additional monitoring and assessment of vital signs will be performed at the discretion of the investigator in accordance with clinical routine

practice.

Subjects may develop the same allergic reactions to study drug as all antibodies. Therefore, it is essential to provide appropriate drugs and medical equipment immediately to treat acute allergic reactions, and the study staff must be trained to recognize and treat systemic allergic reactions. Subjects will be monitored for signs and symptoms of infusion-related reactions and serious systemic allergic reactions. Refer to Section 3.1.3and Table 3 for toxicity management guidelines of infusion reactions.

5.1.5. Labeling and Packaging

Akeso Tiancheng (Guangdong) Co., Ltd. shall uniformly provide labeling and packaging for clinical study drugs for clinical trials.

5.1.6. Storage

AK105 is stored at 2 °C to 8 °C and protected from light. Avoid exposure to temperatures beyond the recommended range and avoid vigorous shaking. If the storage temperature is above 8°C or below 2°C, a temperature excursion will need to be reported and the sponsor will be notified promptly and the impact of this excursion on the product will be evaluated by the sponsor.

Records of the actual storage conditions during the study (e.g., date and time, continuous temperature records of the freezer used to store trial supplies, or maintenance records of instruments such as temperature alarm systems used in conjunction with temperature recorders) must be maintained.

5.1.7. Drug Accountability

The medications provided in this study may only be used under the direction of the protocol. The investigator/institution is responsible for establishing a study treatment drug (including investigational product) management process to ensure that:

- Delivered study drugs are correctly received by authorized personnel.
- Such deliveries are recorded.
- Study treatment medications are handled and stored safely and appropriately as specified on the label.
- Only study treatment medications are assigned to study subjects per protocol.
- Unused products should be destroyed after contacting the clinical research associate. After the sponsor receives a copy of the SOP for Drug Handling and Destruction from the site, unused study drug supplies may be returned to the sponsor for destruction, or destroyed at the site with written approval from the sponsor, in accordance with the SOP of the site.

At the end of the study, the use and destruction records must be consistent with the delivery records. Use records should include the identification of the person dispensing the study treatment medication and the quantity and date dispensed. This record complements any drug

accountability information recorded in the CRF. Any discrepancies must be explained in appropriate forms.

All study drug-related forms must be inspected by an authorized representative or designee of the sponsor and inspectors from regulatory authorities. The investigator or appropriate authorized person is responsible for the accountability of all used and unused study drugs at the study site.

5.1.8. Reporting Drug Complaints

Any deficiencies in the study drug must be immediately reported to the sponsor's Product Complaints Department by study site and further notified to the monitor in the study site. All deficiencies will be notified to the sponsor and further investigated with Product Complaints Department. During the investigation of product complaints, all study drugs must be stored under labeling conditions unless otherwise indicated. Contact Information for Reporting Product Complaints:

Email:	productcomplaints@akesobio.com
Tel.:	+86-760-89873999
Fax:	+86-760-89873900
Company	Akeso Tiancheng (Guangdong) Co., Ltd.
Department	Product Complaints Department
Contact Address	Building A1, No. 6, Shennong Road, Torch Development Zone, Zhongshan City, Guangdong Province, China

5.2. Concomitant Medications and Therapies

If appropriate supportive care is deemed necessary, the investigator may prescribe concomitant medications or treatments, with the exception of "excluded" medications listed in Section 5.2.1. All concomitant medications taken during the study will be recorded in the CRF. Medications taken within 30 days prior to screening will also be recorded in the CRF.

5.2.1. Prohibited Concomitant Medications

Subjects should be informed that all medications taken during the study should be truthfully informed to the investigator.

The following medications are prohibited during the study: if a subject receives any of the following medications during the study, the sponsor must be notified.

1) Any Other Investigational Drug.
2) Any anti-cancer therapy, antibody-based therapy, retinoid therapy, hormonal therapy, treatment with nitrosourea, mitomycin C, small molecule tyrosine kinase inhibitor, Chinese patent medicines with anti-tumor activity, or radiotherapy (except for palliative radiotherapy for known metastatic sites, provided that the tumor imaging assessment is not affected or the time of interruption not exceed the maximum time

of dose modification specified in Section 3.1.4). Palliative radiotherapy for target lesions is not allowed.

- Concomitant use of hormones for non-cancer-related diseases (eg, insulin and hormone replacement therapy for diabetes) is acceptable.

3) Immunosuppressive drugs include, but not limited to, systemic glucocorticoids at doses exceeding 10 mg daily prednisone or equivalent drugs, methotrexate, azathioprine, and TNF-α antagonists.

   - The use of immunosuppressive medications for study drug-related AEs or immunosuppressive medications in subjects with contrast agent allergy is acceptable.
   - In addition, inhaled, topical, and intranasal glucocorticoids are allowed.
   - Short-term use of glucocorticoids for underlying or concurrent conditions may be allowed after discussion with the medical monitor.

4) Live or attenuated vaccine (during the study until Day 120 after the last dose).

5) Concomitant use of dietary supplements, non-investigator-prescribed medications, and alternative/complementary therapies is discouraged but not prohibited, and should be discussed with the medical monitor.

6) Prior medications to prevent infusion-related reactions (i.e., primary prevention) are not allowed prior to the first infusion of AK105; after an infusion-related reaction, any planned prophylaxis is allowed in subsequent infusions of AK105 (i.e., secondary prevention, see Section 3.1.3.1).

6. STUDY PROCEDURES

6.1. Enrollment/Screening Period

A signed ICF must be obtained from the subject before any study-specific procedures or assessments are performed.

Table 6 shows all procedures performed during screening.

Table 6 Screening Procedure Schedule

Study Period	Screening
procedure	Day -28 to Day -1
ICF/assignment of SID number	X
Demographic data and medical history (including smoking history) [a]	X
Tumor history and prior tumor therapy [b]	X
Inclusion/exclusion criteria	X
Physical examination, height, weight	X
Vital signs	X
ECG[c]	X
Echocardiography [d]	X
ECOG performance status	X
Routine Urinalysis (see Section 9.4)	X
Blood chemistry (see Section 9.4)	X
Hematology (see Section 9.4)	X
Thyroid function test (TSH, free T3, free T4)	X
Pregnancy test, serum β-hCG [e]	X
Coagulation (see Section 9.4)	X
AE/SAE assessment	X
Concomitant medications/concomitant therapy [f]	X
Hepatitis B and C; HIV [g]	X
Archival tumor tissue sample/fresh tissue biopsy [h]	X
Tumor imaging assessments (RECIST v1.1) [i, j]	X

Abbreviations: AE = adverse event; β-hCG = subunit of beta human chorionic gonadotropin; CT = computed tomography; ECG = electrocardiogram; ECOG = Eastern Cooperative Oncology Group; HIV = human immunodeficiency virus; MRI = magnetic resonance imaging; RECIST = Response Evaluation Criteria in Solid Tumors; SAE = serious adverse event; SID = subject identification; TSH = thyroid stimulating hormone

a. Demographic data: including date of birth, gender, ethnicity; medical history (all medical history and drug allergy history started before administration and outside this indication);

b. Tumor history and prior tumor therapy: including pathological diagnosis of tumor, previous surgical

history, chemotherapy history, targeted therapy history, immunotherapy history, radiotherapy history;

c. At screening, a single ECG will be performed;

d. Screening only and subsequent testing as clinically indicated;

e. Only for women of childbearing potential, if serum pregnancy tests are not available, urine pregnancy tests are available;

f. Concomitant medications/concomitant therapies: Record medications and concomitant therapies for diseases other than those for indication within the first 30 days prior to screening;

g. Testing will be performed by the local laboratory. Hepatitis B virus screening includes HBsAg, HBsAb, and HBcAb (HBV DNA by hepatitis B virus DNA load test if necessary) and hepatitis C antibody (qualitative testing for hepatitis C RNA if antibody test is positive) and HIV-Ab antibodies;

h. For all subjects, if archival tumor biopsy samples (tissue samples within 3 years prior to enrollment) are available, no fresh tumor biopsy is required at screening and archival tumor biopsy samples must be provided, both optional. If an archival tumor tissue sample is not suitable for use, a fresh tumor biopsy at baseline will be required prior to the subject's first dose of study drug. It is preferred that newly obtained tumor tissue samples should be sent to a central laboratory for IHC determination of PD-L1 (see Section 8.3.1 for instructions on tumor tissue collection).

i. CT scan or MRI of the nasopharynx, neck, chest, abdomen, and pelvis are required for baseline assessments at screening. Bone scans are also required for baseline assessments at screening. If there is evidence of metastases to any other site at screening, CT scans of these sites should be performed based on the clinical symptoms and signs of the individual subject and followed throughout the study. Brain MRI (CT may be used instead of MRI if MRI is contraindicated) is required for suspected and confirmed brain metastases . All known lesion sites must be recorded at screening and reassessed at each subsequent tumor assessment. At the investigator's discretion, other methods may be used to assess measurable disease per RECIST v1.1. To assess response, baseline and post-treatment test methods should be consistent to facilitate comparisons.

j. Baseline assessments should be completed within 28 days prior to the first dose and ideally as close as possible to the time of first dose of AK105. The results of tumor assessments performed prior to obtaining informed consent and within 28 days prior to the first dose can be used for screening assessments rather than repeating such assessments. Bone scans are not required to be repeated if they have been performed within 42 days prior to dosing on Day 1.

6.2. Treatment Period

Each cycle is 28 days during the treatment period. The study procedures during the treatment period from Cycle 1 to Cycle 6 are presented in Table 7. The study procedures from Cycle 7 to Cycle 13 and subsequent cycles are presented in Table 8. During the treatment period, the investigator may increase the number of laboratory tests or other laboratory tests as required by the patient's condition.

If the following assessments have been completed within 72 h prior to the first dose of study drug, repeated assessments are not required on Day 1: 12-lead ECG, hematology, blood

chemistry, routine urinalysis, and pregnancy tests; if the following assessments have been completed within 1 week prior to the first dose of study drug, repeated assessments are not required on Day 1: thyroid function tests.

Table 7 Study procedures from Cycle 1 to Cycle 6 during the treatment period

**Procedures/Study Days** [a]	**During the Treatment Period**											
	**Cycle 1**		**Cycle 2**		**Cycle 3**		**Cycle 4**		**Cycle 5**		**Cycle 6**	
	**D1**	**D15 ±3**	**D1 ±3**	**D15 ±3**	**D1 ±3**	**D15 ±3**	**D1 ±3**	**D15 ±3**	**D1 ±3**	**D15 ±3**	**D1 ±3**	**D15 ±3**
	**D1**	**D15 ±3**	**D29 ±3**	**D43 ±3**	**D57 ±3**	**D71 ±3**	**D85 ±3**	**D99 ±3**	**D113 ±3**	**D127 ±3**	**D141 ±3**	**D155 ±3**
**Tumor Imaging Assessment**												
Tumor imaging assessments by RECIST v1.1 Assessment (including use of appropriate imaging techniques) [b, c]					X (D56±7)				X (D112±7)			
**Study Procedures and Test Items**												
Inclusion/exclusion criteria	X											
Physical examination (including body weight) [d]	X	X	X	X	X	X	X	X	X	X	X	X
ECOG performance status	X	X	X	X	X	X	X	X	X	X	X	X
ECG[e]	X		X		X				X			
Vital signs [f]	X	X	X	X	X	X	X	X	X	X	X	X
Echocardiography [g]												
Concomitant medications/treatment	X	X	X	X	X	X	X	X	X	X	X	X
AE/SAE assessment	X	X	X	X	X	X	X	X	X	X	X	X
**Laboratory Tests**												
Hematology (see Section 9.3)	X	X	X	X	X	X	X	X	X	X	X	X
Blood chemistry (see Section 9.3)	X	X	X	X	X	X	X	X	X	X	X	X
Thyroid function test: TSH, free T4 and free T3	X		X		X		X		X		X	
Routine Urinalysis (see Section 9.3) [h]	X	X	X	X	X	X	X	X	X	X	X	X

Procedures/Study Days [a]	During the Treatment Period											
	Cycle 1		Cycle 2		Cycle 3		Cycle 4		Cycle 5		Cycle 6	
	D1	D15 ±3	D1 ±3	D15 ±3	D1 ±3	D15 ±3	D1 ±3	D15 ±3	D1 ±3	D15 ±3	D1 ±3	D15 ±3
	D1	D15 ±3	D29 ±3	D43 ±3	D57 ±3	D71 ±3	D85 ±3	D99 ±3	D113 ±3	D127 ±3	D141 ±3	D155 ±3
Pregnancy test [i]	X	As clinically indicated										
**Pharmacokinetics and Immunogenicity**												
AK105 PK (intensive sampling) (refer to Table 10) [j]	X [k,l]	X [k]	X [k]	X [k]	X [k]	X [k,m]	X [k]		X [k]		X [k]	
AK105 PK (sparse sampling) (see Table 11) [j]	X [k]		X [k]		X [k]		X [k]		X [k]		X [k]	
Immunogenicity [n]	X		X		X		X		X		X	
**Study Drug Administration**												
AK105 administration	X	X	X	X	X	X	X	X	X	X	X	X
**Biomarkers**												
Plasma EBV-DNA	X		X		X				X			

Abbreviations: AE = adverse event; D = day; ECG = electrocardiogram; ECOG = Eastern Cooperative Oncology Group; PK = pharmacokinetics; Q8W = once every 8 weeks; RECIST = Response Evaluation Criteria in Solid Tumors; SAE = serious adverse event; TSH = thyroid stimulating hormone.

Note: all samples to be collected on dosing days are collected prior to dosing unless otherwise specified. AK105 will be administered by infusion every 2 weeks. Continuous infusion of AK105 must maintain a dosing interval of at least 12 days and no more than 18 days. Each cycle is 28 days.

a. Nominal assessments and laboratory tests on Day 1 will be performed prior to AK105 administration. If the following assessments have been completed within 72 h prior to the first dose of study drug, repeated assessments are not required on Day 1 of Cycle 1: 12-lead ECG, hematology, blood chemistry, routine urinalysis, and pregnancy tests; if the following assessments have been completed within 1 week prior to the first dose of study drug, repeated assessments are not required on Day 1 of Cycle 1: thyroid function tests.

b. During AK105 treatment, assessments will be performed every 8 weeks (± 7 days) in the first year until initiation of new anti-tumor therapy, first or re-occurrence of

disease progression, subject's withdrawal of ICF, or death. Assessments should be obtained before the scheduled time of study drug administration. If radiological disease progression is observed in the absence of clinical deterioration, a confirmatory scan should be performed no less than 4 weeks later. For subjects who discontinue treatment for reasons other than radiological disease progression, tumor assessments should be continued whenever possible: assessments should be performed every 8 weeks (± 7 days) in the first year (relative to the date of the first dose of AK105), until initiation of new anti-tumor therapy, disease progression, withdrawal of ICF by subject, or death. The investigator may perform additional scans or more frequent assessments if clinically indicated. Unscheduled radiographic tests may be performed when disease progression is suspected (eg, worsening symptoms). The same radiology procedures must be followed throughout the study for each subject. Assessments should be performed by the same assessor, if possible, to ensure internal consistency across visits. In the event of treatment delay, tumor assessments should be performed as originally planned.

c. Bone scans should be repeated if bone metastases are present at screening but are not seen on CT or MRI scans, or if clinically indicated, when complete response is identified in target lesions or when progression of bone metastases is suspected. Objective response should be confirmed by repeat assessments ≥4 weeks after the first demonstration of response. Following confirmatory scans, tumor assessments should be performed as originally planned.

d. A targeted physical examination should be performed based on symptoms and prior to administration of AK105.

e. A single examination is performed for all ECGs. ECGs will be recorded within 24 h prior to the start of infusion on the date of administration on which an ECG is required, unless otherwise stated or clinically indicated. In the event of chest pain, palpitations, or other cardiac symptoms, ECG or echocardiography may be additionally performed at any time as clinically indicated.

f. On the dosing date of AK105, vital signs will be assessed within 30 min before the start of infusion, 30 min (± 10 min) during 1 h infusion, (+ 10 min) after the end of infusion, 30 min (± 10 min) and 60 min (± 10 min) after the end of infusion. On Day 1 of Cycle 1, additional vital sign measurements will be performed 3 h (± 15 min) after the first infusion. For subsequent infusions, a 3-h observation period is not required unless the subject experiences an infusion reaction. If a subject experiences an infusion-related reaction, additional vital sign measurements will be performed 3 h after infusion and 3 h after each subsequent infusion. Additional monitoring and assessment of vital signs will be performed at the discretion of the investigator in accordance with clinical routine practice.

g. Testing is performed as clinically indicated.

h. A 24-h urine collection should be provided for further analysis if the subject shows proteinuria 2+ or higher on urine dipstick or routine analysis.

i. Only for women of childbearing potential. A serum pregnancy test (if serum pregnancy test is not available, urine pregnancy test may be adopted) will be performed and documented as negative within 3 days prior to the first dose at screening. Urine or serum pregnancy tests will be performed as clinically indicated during treatment and at the safety follow-up. If a urine pregnancy test is positive, a serum pregnancy test must be performed (at each study site or designated laboratory), whichever is the serum pregnancy result.

j. Unless otherwise specified, PK blood sampling will be performed within 30 min before the start of AK105 infusion.

k. PK blood sampling will be performed within 30 min before the start of AK105 infusion and (+ 5 min) at the end of infusion.

l. For PK blood sampling only for subjects requiring intensive sampling. A total of 6 PK blood sampling points are set after the first dose: within 30 min before the start of AK105 infusion, immediately (+ 5 min) after the end of infusion, 3 h ± 15 min, 24 h ± 3 h (Day 2), 48 h ± 3 h (Day 3), 168 h ± 1 day (Day 8) after the end of infusion;

m. For PK blood sampling only for subjects requiring intensive sampling. In Cycle 3, a total of 6 PK blood sampling points are set for the sixth dose (Day 71): within 30 min before the start of AK105 infusion, immediately (+ 5 min) after the end of infusion, 3 h ± 15 min, 24 h ± 3 h (Day 72), 48 h ± 3 h (Day 73), 168 h ± 1 day (Day 78) after the end of infusion.

n. Blood samples for immunogenicity are collected prior to study drug infusion in each cycle.

Table 8 Study procedures from Cycle 7 to Cycle 13 and subsequent cycles during treatment

Procedure/Study Days	During the Treatment Period													
	Cycle 7		Cycle 8		Cycle 9		Cycle 10		Cycle 11		Cycle 12		Cycle 13 and subsequent cycles	
	D1 ±3	D15 ±3	D1 ±3	D15 ±3	D1 ±3	D15 ±3	D1 ±3	D15 ±3	D1 ±3	D15 ±3	D1 ±3	D15 ±3	D1 ±3	D15 ±3
	D169 ±3	D183 ±3	D197 ±3	D211 ±3	D225 ±3	D239 ±3	D253 ±3	D267 ±3	D281 ±3	D295 ±3	D309 ±3	D323 ±3	D337 ±3	D351 ±3
**Tumor Imaging Assessment**														
Tumor imaging assessments by RECIST v1.1 (including use of appropriate imaging techniques) [a, b]	X (D168±7)				X (D224±7)				X (D280±7)				X (every 12 weeks ± 7)	
**Study Procedures and Test Items**														
Physical examination (including body weight) [c]	X	X	X	X	X	X	X	X	X	X	X	X	X	X
ECOG performance status	X	X	X	X	X	X	X	X	X	X	X	X	X	X
ECG[d]	X				X				X				X (every 8 weeks)	
Vital signs [e]	X	X	X	X	X	X	X	X	X	X	X	X	X	X
Echocardiogram [f]														
Concomitant medications/treatment	X	X	X	X	X	X	X	X	X	X	X	X	X	X
AE/SAE assessment	X	X	X	X	X	X	X	X	X	X	X	X	X	X
**Laboratory Tests**														
Hematology (see Section 9.3)	X	X	X	X	X	X	X	X	X	X	X	X	X	X
Blood chemistry (see Section 9.3)	X	X	X	X	X	X	X	X	X	X	X	X	X	X
Thyroid function test: TSH, free T4 and free T3	X		X		X		X		X		X		X	
Routine Urinalysis (see Section 9.3) [g]	X	X	X	X	X	X	X	X	X	X	X	X	X	X

Procedure/Study Days	During the Treatment Period													
	Cycle 7		Cycle 8		Cycle 9		Cycle 10		Cycle 11		Cycle 12		Cycle 13 and subsequent cycles	
	D1 ±3	D15 ±3	D1 ±3	D15 ±3	D1 ±3	D15 ±3	D1 ±3	D15 ±3	D1 ±3	D15 ±3	D1 ±3	D15 ±3	D1 ±3	D15 ±3
	D169 ±3	D183 ±3	D197 ±3	D211 ±3	D225 ±3	D239 ±3	D253 ±3	D267 ±3	D281 ±3	D295 ±3	D309 ±3	D323 ±3	D337 ±3	D351 ±3
Pregnancy test [h]	As clinically indicated													
**Pharmacokinetics and Immunogenicity**														
AK105 PK[i]	X						X						X (every 12 weeks)	
Immunogenicity [j]	X						X						X (every 12 weeks)	
**Study Drug Administration**														
AK105 administration	X	X	X	X	X	X	X	X	X	X	X	X	X	X
**Biomarkers**														
Plasma EBV-DNA	X				X				X				X (every 12 weeks)	

Abbreviations: AE = adverse event; D = day; ECG = electrocardiogram; ECOG = Eastern Cooperative Oncology Group; PK = pharmacokinetic; Q8W = once every 8 weeks; RECIST = Response Evaluation Criteria in Solid Tumors; SAE = serious adverse event; TSH = thyroid stimulating hormone.

Note: all samples to be collected on dosing days are collected prior to dosing unless otherwise specified. AK105 will be administered by infusion every 2 weeks. Continuous infusion of AK105 must maintain a dosing interval of at least 12 days and no more than 18 days. Each cycle is 28 days.

a. During AK105 treatment, assessments will be performed every 8 weeks (± 7 days) in the first year and every 12 weeks (± 7 days) thereafter until the start of new anti-tumor therapy, the first or re-occurrence of disease progression, subject's withdrawal of ICF, or death. Assessments should be obtained before the scheduled time of study drug administration. If radiological disease progression is observed in the absence of clinical deterioration, a confirmatory scan should be performed no less than 4 weeks later. For subjects who discontinue treatment for reasons other than radiological disease progression, tumor assessments should be continued whenever possible: assessments should be performed every 8 weeks (± 7 days) in the first year (relative to the date of the first dose of AK105) and every 12 weeks (± 7 days) after one year (starting at Week 56), until initiation of new anti-tumor therapy, disease progression, withdrawal of ICF by subject, or death. The investigator may perform additional scans or more frequent assessments if clinically indicated. Unscheduled radiographic tests may be performed when disease progression is suspected (eg, worsening symptoms). The same radiology procedures must be followed throughout the study for each subject. Assessments should be

performed by the same assessor, if possible, to ensure internal consistency across visits.

[b.] Bone scans should be repeated if bone metastases are present at screening but are not seen on CT or MRI scans, or if clinically indicated, when complete response is identified in target lesions or when progression of bone metastases is suspected. Objective response should be confirmed by repeat assessments ≥4 weeks after the first demonstration of response. Following confirmatory scans, tumor assessments should be performed as originally planned.

[c.] A targeted physical examination should be performed based on symptoms and prior to administration of AK105.

[d.] A single examination is performed for all ECGs. ECGs will be recorded within 24 h prior to the start of infusion on the date of administration on which an ECG is required, unless otherwise stated or clinically indicated. In the event of chest pain, palpitations, or other cardiac symptoms, ECG or echocardiography may be additionally performed at any time as clinically indicated.

[e.] On the dosing date of AK105, vital signs will be assessed within 30 min before the start of infusion, 30 min (± 10 min) during 1 h infusion, (+ 10 min) after the end of infusion, 30 min (± 10 min) and 60 min (± 10 min) after the end of infusion. If a subject experiences an infusion-related reaction, additional vital sign measurements will be performed 3 h after infusion and 3 h after each subsequent infusion. Additional monitoring and assessment of vital signs will be performed at the discretion of the investigator in accordance with clinical routine practice.

[f.] Testing is performed as clinically indicated.

[g.] A 24-h urine collection should be provided for further analysis if the subject shows proteinuria 2+ or higher on urine dipstick or routine analysis.

[h.] Only for women of childbearing potential. Urine or serum pregnancy tests will be performed as clinically indicated during treatment and at the safety follow-up. If a urine pregnancy test is positive, a serum pregnancy test must be performed (at each study site or designated laboratory), whichever is the serum pregnancy result.

[i.] Unless otherwise specified, PK blood sampling will be performed within 30 min before the start of AK105 infusion.

[j.] Blood samples for immunogenicity are collected prior to study drug infusion in each cycle.

6.3. End of Treatment and Follow-up Period

Table 9 shows all procedures performed during the end of treatment follow-up and follow-up period for (1) subjects who discontinue study drug due to toxicity in the absence of PD and (2) subjects with confirmed PD.

Subjects will continue to participate in follow-up until death, withdrawal of consent, loss to follow-up, or end of study, whichever occurs earliest.

Table 9 End of treatment schedule and follow-up procedure

**Procedure/Study Days**	**Follow-up Period**		
	**Follow-up after End of Treatment (D 30 ± 3 after last dose) [a]**	**D 90 (± 7) after last dose**	**Every 3 months (± 14 D) after last dose**
AE/SAE assessment	X	X	
Concomitant medications/treatment	X	X [b]	
Physical examinations (including weight)	X		
ECOG performance status	X		
Vital signs	X		
ECG[c]	X		
**Tumor Imaging Evaluation**			
Tumor imaging assessments by RECIST v1.1 (Including use of appropriate imaging techniques)	**See footnote [d]**<br>(Every 8 weeks for the first 12 months and every 12 weeks thereafter)		
**Laboratory Tests**			
Routine Urinalysis (see Section 9.4) [e]	X		
Blood chemistry (see Section 9.4)	X		
Thyroid function test (TSH, free T3, free T4)	X		
Hematology (see Section 9.4)	X		
Pregnancy test [f]	X		
**Pharmacokinetics and Immunogenicity**			
AK105 PK	X	X	
Immunogenicity	X	X	
**Biomarkers**			
Plasma EBV-DNA	X		
**Survival Follow-up and Subsequent Anti-cancer Therapy**		X	X

Abbreviations: AE = adverse event; ECG = electrocardiogram; ECOG = Eastern Cooperative Oncology Group; hCG = human chorionic gonadotropin; PK = pharmacokinetics; RECIST = Response Evaluation Criteria in Solid Tumors; SAE = serious adverse event; TSH = thyroid stimulating hormone.

a. Follow-up at the end of treatment will start 30 days ± 3 days after the last dose of AK105, or

before the start of a new anti-tumor treatment, whichever occurs first, or if the drug discontinuation duration is more than 33 days from the last dose of AK105, the follow-up date at the end of the study is the date (± 3 days) when AK105 discontinues.

b. The collection of concomitant medications will be discontinued 90 days after the last dose, unless the subject continues to experience an AE while on treatment, which should continue to collect concomitant medications until the event resolves.

c. A single examination is performed for ECG.

d. For subjects who discontinue study drug due to toxicity or reasons other than disease progression, tumor assessments should be performed according to the schedule shown below for the date of first dose: every 8 weeks (± 7 days) for the first 12 months (relative to the date of the first dose of AK105), every 12 weeks (± 7 days) thereafter until initiation of new anti-tumor therapy, disease progression, withdrawal of ICF by subject, or death. The investigator may perform additional scans or more frequent assessments if clinically indicated. Unscheduled radiographic tests may be performed when disease progression is suspected (eg, worsening symptoms). The same radiology procedures must be followed throughout the study for each subject. Assessments should be performed by the same assessor, if possible, to ensure internal consistency across visits. If radiological disease progression is observed in the absence of clinical deterioration, a confirmatory scan should be performed no less than 4 weeks later.

e. A 24-h urine collection should be provided for further analysis if the subject shows proteinuria 2+ or higher on urine dipstick or routine analysis.

f. Only for women of childbearing potential. Urine or serum pregnancy tests will be performed as clinically indicated during treatment and at the safety follow-up. If a urine pregnancy test is positive, a serum pregnancy test must be performed (at each study site or designated laboratory), whichever is the serum pregnancy result.

7. EFFICACY ASSESSMENTS

7.1. Tumor Response Assessed per RECIST v1.1

Tumor response assessments will be performed based on RECIST guidelines (version 1.1) and according to the schedule in Section 6. The schedule of assessments also applies to subjects who have radiological disease progression but continue to receive AK105. Guidelines for RECIST v1.1 are presented in **Appendix 1** for measurable, non-measurable, target and non-target lesions and objective tumor response criteria (CR, PR, SD, or PD). For subjects receiving the treatment, the clinical decision will be made by investigator's assessment per RECIST v1.1, including confirmation of progressive disease.

Baseline assessments should be completed within 28 days prior to the first dose and ideally as close as possible to the time of first dose of AK105. The results of tumor assessments performed prior to obtaining ICF and within 28 days prior to the first dose can be used for screening assessments rather than repeating such assessments. Follow-up tumor assessments are performed every 8 weeks (±7 days) (relative to the date of the first dose of AK105) during the first 12 months of treatment, and then every 12 weeks (±7 days) until the patient starts subsequent anti-cancer therapy, has confirmed disease progression, withdraws ICF, dies, or until the study is closed, whichever occurs first. Confirmatory scans should be performed at the next follow-up visit and within no less than 4 weeks after the initial assessment of disease progression (without significant clinical deterioration). If the performance status of the subject is stable, the investigator believes that the subject can benefit from continuous treatment, and the subject meets other criteria (see Section 3.1.1) listed in the protocol, the subject may continue to receive study treatment until intolerable toxicity occurs, there is no longer clinical benefit as judged by the investigator (as assessed by the investigator via imaging, laboratory tests, clinical status), death or loss to follow-up, subject's withdrawal of ICF, and study termination by the sponsor, whichever occurs first. In the event of treatment delay, tumor assessments should be performed as originally planned.

Response will be assessed by the investigator using RECIST v1.1 (see **Appendix 1**). Assessments should be performed by the same assessor, if possible, to ensure internal consistency across visits. Objective response should be confirmed by repeat imaging assessments ≥ 4 weeks after the first demonstration of response. Following confirmatory scans, tumor assessments should be performed as originally planned.

CT scan or MRI of the nasopharynx, neck, chest, abdomen, and pelvis are required for baseline assessments at screening. Bone scans are also required for baseline assessments at screening. Bone scans are not required to be repeated if they have been performed within 42 days prior to dosing on Day 1. Bone scans should be repeated if bone metastases are present at screening but are not seen on CT or MRI scans, or if clinically indicated, when complete response is identified in target lesions or when progression of bone metastases is suspected. If there is evidence of metastases to any other site at screening, CT scans of these sites should be performed based on the clinical symptoms and signs of the individual subject and followed throughout the study. Brain MRI (CT may be used instead if MRI is contraindicated) is

required for suspected and confirmed brain metastases . For subsequent tumor assessments, the same radiological procedures used to assess disease sites at screening are required throughout the study, e.g., the same contrast regimen for CT scans. All known lesion sites must be recorded at screening and reassessed at each subsequent tumor assessment. For subjects who discontinue study drug due to toxicity or for reasons other than confirmed disease progression, tumor assessments should be performed every 8 weeks (± 7 days) for the first 12 months (starting at Week 56 relative to the date of the first dose of AK105), and then every 12 weeks (± 7 days) until the patient starts subsequent anticancer therapy, has confirmed disease progression, withdraws ICF, dies, or until the study is closed, whichever occurs first. The investigator may perform additional scans or more frequent assessments if clinically indicated. Unscheduled radiographic tests may be performed when disease progression is suspected (eg, worsening symptoms). If an unscheduled assessment is performed and the subject has no progression, tumor assessments will need to follow the original plan for subsequent assessments.

The primary endpoint of this study is ORR assessed by IRRC per RECIST v1.1. The sponsor will designate a contract research organization to conduct independent central imaging assessments for this study. Refer to the IRRC charter for specific imaging requirements and guidance on the collection of results.

7.2. Exploratory Evaluation of Tumor Response Using irRECIST

Responses to immunotherapy may differ from typical responses observed with cytotoxic chemotherapy, including clinical objective responses and/or stable disease that may occur after disease progression, and new lesions that do not represent disease progression with immunotherapy, from which clinical benefit is still possible. After initial disease progression (per RECIST v1.1), the investigator will assess whether continued AK105 treatment can provide clinical benefit to patients (e.g., immune-related partial response (irPR); immune-related stable disease (irSD)) (see Section 3.1.2).

The sponsor may designate a contract research organization to assess immunotherapy for cancer per irRECIST (**Nishino M, 2013**) for this study. It differs from RECIST v1.1 in the following key aspects:

1) The appearance of new lesions represents PD per RECIST v1.1. However, new measurable lesions are included in the tumor burden to determine immune-related disease progression (irPD), immune-related partial response (irPR), and immune-related complete response (irCR) according to irRECIST. irCRs are excluded from new non-measurable diseases.
2) No confirmation of PD is performed per RECIST v1.1. Consecutive scans (with an interval of at least 4 weeks) are required to determine response and irPD, assuming no clinical deterioration per irRECIST.

Guidelines for irRECIST are provided in **Appendix 1** for measurable, non-measurable, target and non-target lesions, and objective tumor response criteria (irCR, irPR, irSD, or irPD).

7.3. Survival Assessment

Survival assessments will be performed every 3 months after the discontinuation of treatment drug. Survival information can be obtained by telephone contact with the patient or patient's family, or by contacting the patient's current physician, or by reviewing clinical records from the case. Information on the first and subsequent cancer treatment after discontinuation will be collected. In addition, patients may be contacted at the time of treatment or survival follow-up to obtain complete survival data after the data cutoff date for the primary analysis and all subsequent survival analyses. Contacts are usually made within 7 days of the data cutoff.

8. PHARMACOKINETIC/IMMUNOGENICITY ASSESSMENT

8.1. Pharmacokinetics

PK sample collection will be performed at sites with sufficient capacity for PK sampling and sample processing, and blood samples will be collected at the time points indicated in Sections 6.2 and 6.3 (refer to Table 10 and Table 11), approximately 4 mL of peripheral venous blood will be collected each time for PK analysis. Sparsely collected PK blood samples will be continuously evaluated for PK parameters of AK105 and their correlation with demographic variables and clinical endpoints using the population PK method. Intensive PK samples will be collected from 6-9 subjects at selected sites, the time points are shown in Table 10. For these subjects, PK parameters for each AK105 concentration will be calculated using non-compartmental methods. For the remaining subjects, PK samples will be sparsely sampled at the time points shown in Table 11. Subject samples will be analyzed for AK105 concentrations at a central laboratory using a validated bioanalytical method. Specific instructions on serum PK sample collection and transfer will be provided in the laboratory manual. If a subject experiences any ≥ Grade 3 irAE or infusion reaction, additional blood samples may be collected for determination of serum concentrations of AK105, ADA, etc.

Table 10 PK sampling schedule for AK105 (intensive sampling)

**Number of Doses (Cycles)**	**Days**	**Time Relative to AK105 Infusion**
Dose 1 (Cycle 1)	D1	Pre-infusion (within 30 min before infusion)
		End of infusion (+ 5 min)
		3 h (± 15 min) after the end of infusion
	D2	24 h (± 3 h) after the end of infusion
	D3	48 h (± 3 h) after the end of infusion
	D8	168 h (± 1 day) after the end of infusion
Dose 2 (Cycle 1)	D15	Pre-infusion (within 30 min before infusion)
		End of infusion (+ 5 min)
Dose 3 (Cycle 2)	D29	Pre-infusion (within 30 min before infusion)
		End of infusion (+ 5 min)
Dose 4 (Cycle 2)	D43	Pre-infusion (within 30 min before infusion)
		End of infusion (+ 5 min)
Dose 5 (Cycle 3)	D57	Pre-infusion (within 30 min before infusion)
		End of infusion (+ 5 min)
Dose 6 (Cycle 3)	D71	Pre-infusion (within 30 min before infusion)
		End of infusion (+ 5 min)
		3 h (± 15 min) after the end of infusion
	D72	24 h (± 3 h) after the end of infusion
	D73	48 h (± 3 h) after the end of infusion
	D78	168 h (± 1 day) after the end of infusion
Dose 7 (Cycle 4) or subsequent dosing	D85 and after	For Cycles 4, 5, and 6, pre-infusion (within 30

(every 4 weeks until Cycle 7; then every 12 weeks)	D85	min before infusion) and after infusion (+ 5 min). Subsequently, only pre-infusion samples (within 30 min before infusion) are collected.
Follow-up at the end of the study (D30 ± 3, after the last dose)		
D90 ± 7 after the last dose		

Table 11 PK sampling schedule for AK105 (sparse sampling)

Number of Doses (Cycles)	Days	Time Relative to AK105 Infusion
Dose 1 (Cycle 1)	D1	Pre-infusion (within 30 min before infusion)
		End of infusion (+ 5 min)
Dose 3 (Cycle 2)	D29	Pre-infusion (within 30 min before infusion)
		End of infusion (+ 5 min)
Dose 5 (Cycle 3)	D57	Pre-infusion (within 30 min before infusion)
		End of infusion (+ 5 min)
Dose 7 (Cycle 4)	D85	Pre-infusion (within 30 min before infusion)
		End of infusion (+ 5 min)
Dose 9 (Cycle 5)	D113	Pre-infusion (within 30 min before infusion)
		End of infusion (+ 5 min)
Dose 11 (Cycle 6)	D141	Pre-infusion (within 30 min before infusion)
		End of infusion (+ 5 min)
Dose 13 or subsequent doses (Cycle 7 or subsequent cycles) every 12 weeks	D169 and after D169	Pre-infusion (within 30 min before infusion)
Follow-up at the end of the study (D30 ± 3, after the last dose)		
D90 ± 7 after the last dose		

8.2. Immunogenicity

ADA sample collection will be performed at sites with sufficient capacity for ADA sampling and sample processing, and 4 mL of blood will be collected each time for the determination of anti-AK105 antibody titers in serum (refer to Sections 6.2 and 6.3 for sampling time points). The following bridging immunoassays will be developed and validated. Once the tested sample is confirmed to be ADA positive, the specificity of ADA will be assessed to confirm whether ADA-positive serum samples are neutralizing antibodies (Nabs). Samples will be collected and stored to assess the specificity of ADA and this step will be performed after completion of assay development and validation for the detection of neutralizing antibodies.

Detailed information on blood sample collection, processing, storage, and transfer of serum samples for ADA evaluation are provided in the laboratory manual.

8.3. Biomarkers

8.3.1. Archival Tumor Tissue Samples and Tumor Biopsies

Subjects who meet the inclusion criteria will be required to provide archival tumor tissue within 3 years prior to enrollment (at least 3 unstained FFPE pathological sections. If the

samples are insufficient for PD-L1 IHC testing as determined by the site laboratory, 3 additional unstained FFPE pathological sections are required) and sent to the central laboratory designated by the sponsor for PD-L1 IHC testing. If there is no archival tumor tissue or archival tumor tissue is insufficient for PD-L1 IHC testing, fresh tumor biopsies must be collected at screening (at least 3 FFPE pathological sections, and 3 additional unstained FFPE pathological sections are required if the sample is insufficient for PD-L1 IHC testing as determined by the site laboratory). It is preferred that newly obtained tumor tissue samples are sent to a central laboratory for IHC determination of PD-L1.

Fresh tumor biopsies should be obtained preferentially from tumor tissues that are safely collected as determined by the investigator, rather than from the sites that are obtained by major-risk surgery (e.g., brain biopsy) . Tumor lesions planned for biopsy should not be used as target lesions for disease assessment unless there are no other lesions suitable for biopsy. If there is no archival tumor tissue samples within 3 years, and biopsy may increase the subject's risk as judged by the investigator, the archival tumor tissue sample beyond 3 years may be collected after discussion with the medical monitor.

Shipping, storage, and handling of archival tumor, fresh tumor, and leftover tumor tissue for IHC determination of PD-L1 will be managed through a site laboratory. Refer to the Laboratory Manual for details of sample handling.

8.3.2. Biomarkers in Blood

Blood samples will be collected to evaluate the correlation between EBV-DNA expression at baseline and after AK105 treatment and anti-tumor response in blood of subjects with nasopharyngeal carcinoma (refer to Sections 6.2 and 6.3 for sampling time points).

Details of blood sample collection, handling, storage and transfer will be provided in the Laboratory Manual.

9. SAFETY ASSESSMENTS

9.1. Adverse Event

At each follow-up, subjects will be evaluated by the investigator to determine if an AE has occurred. At each study follow-up, adverse events may be observed directly, reported voluntarily by the subject, or questioned by the subject. Subjects should be asked questions about their general condition and not about any specific symptoms. The investigator must assess the clinical significance of all laboratory findings. All abnormal post-baseline laboratory results considered clinically significant by the investigator must be recorded on the AE page of the CRF or electronic data capture (EDC).

All AEs (whether observed by the investigator or reported by the subject) occurring after the subject signs the ICF and up to 90 days after the last dose of AK105 will be recorded on the AE page of the ICF. Abnormalities observed during screening assessments will be recorded as part of the medical history and will not be recorded in the medical history of the eCRF unless judged by the investigator to be an AE. AEs or SAEs are reported using diagnostic medical terms; when the diagnosis is not available, symptoms and signs are reported as separate entries for AEs or SAEs until a final diagnosis is obtained. For death, the cause of death should be reported as an SAE.

All SAEs should be reported according to the investigator' s SAE reporting process described in Section 9.2. In addition, SAEs occurring after the reporting period should also be reported and handled as SAEs if it is considered to be possibly related to study drug by the investigator. A pre-planned process or hospitalization for an existing condition without serious deterioration should not be reported as an SAE (refer to the definition in Section 9.1.1).

The investigator should follow up the subject for adverse events until resolution of the event or until the stability of condition. At the end of the treatment assessment, if unresolved AEs include significantly abnormal test results, these events will be followed up until the event has resolved to baseline, the event is assessed by the investigator as stable, the subject is lost to follow-up, the subject withdraws ICF, or it is confirmed that study treatment or participation is not the cause of the adverse event.

9.1.1. Definition

9.1.1.1. Adverse Event (AE)

AEs represent all adverse medical events that occur in clinical studies after subjects receive study drug, and a clear causal relationship to the study drug is not necessarily inferred. Therefore, whether considered drug-related or not, AE can be any adverse effect and unplanned sign (including, for example, laboratory findings), symptoms or diseases (ICH E2A guidelines) associated with the use of the study drug. Clinical Safety Data Management: Definitions and Standards for the Expedited Reporting System, October 1994).

Investigators are obligated to determine which conditions or abnormal laboratory findings should be AEs based on their knowledge and experience. AEs include, but are not limited to,

the following:

- Worsening of pre-existing (before entering the clinical trial) medical condition/disease (including worsening of symptoms, signs, laboratory abnormalities);
- Any new adverse medical condition (including symptoms, signs, newly diagnosed diseases);
- Abnormal laboratory values or results with clinical significance.

9.1.1.2. Serious adverse event

Any adverse medical event at any dose:

- Death;
- Life-threatening;
- Hospitalization or postponement of discharge from hospital;
- Hospitalization or prolonged hospitalization indicated, excluding the following:
    - ✓ Rehabilitation facilities
    - ✓ Nursing homes
    - ✓ Routine emergency rooms
    - ✓ Day surgery (such as outpatient/daytime surgery/ambulatory surgery)
    - ✓ Any hospitalization or prolongation of existing hospitalization due to any causes other than AE deterioration will not be considered as a SAE. For example, admission due to the original disease, and no new adverse events or aggravation of the original disease (e.g., to check for laboratory abnormalities that persisted prior to the trial); hospitalization for management (e.g., annual physical examination); hospitalization as specified in the protocol during the clinical trial (e.g., procedures as required by the protocol); elective hospitalization not associated with worsening of adverse events (e.g. elective surgery); scheduled treatment or surgery should be recorded throughout the protocol and/or in the individual subject's baseline data; admitted for blood product use only.
- Resulting in permanent or serious disability/incapacity;
- Congenital abnormality/birth defect;
- Other medically significant events.

Note: The term "life-threatening" in the definition of "serious adverse event" refers to the possible death of the subject at the time of the event; it does not refer to a possible death if the event is assumed to be further serious (ICH E2A guideline). Clinical Safety Data Management: Definitions and Standards for the Expedited Reporting System, October 1994).

Medical and scientific judgments should be made in deciding whether expedited reporting is appropriate in other circumstances, for example, the significant medical event may not be

immediately life-threatening, or may result in death, or require hospitalization but may jeopardize the subject, or may require intervention to prevent other consequences listed in the above definition. Examples include allergic bronchospasm, convulsion, and blood cachexia, or drug dependence or drug abuse.

Notes:

- A process is not an AE or SAE, but the cause of the process may be an AE.
- Preplanned surgeries or hospitalization for pre-existing conditions that do not worsen in severity are not SAEs.
- Disease progression at the endpoint is not considered an SAE and is not required to be reported in the SAE report. Disease progression leading to a fatal outcome is not considered an SAE.

9.1.1.3. Adverse event of special interest

Adverse events of special interest (AESIs) are events of special scientific and medical interest that contribute to understanding the safety profile of the investigational product AK105, and require close observation by the investigator and rapid communication with the sponsor/CRO. AESIs may or may not be serious. The expedited reporting of AESIs (only to the sponsor according to the procedures described in Section 9.2) is ongoing monitoring of these events to characterize and understand their relevance to the use of the investigational product.

AK105 is a humanized IgG1 monoclonal antibody directed against PD-1. Potential risks based on mechanism of action are associated with AK105, which activates the immune system. AESIs in this study include, but are not limited to, events with underlying inflammatory or immune-mediated mechanisms and that may require more frequent monitoring and/or intervention, such as treatment with glucocorticoids, immunosuppressants, and/or hormone replacement therapies.

≥grade 3 immune-related AEs (including but not limited to colitis, dermatitis, pneumonia, hepatitis, encephalitis, nephritis, and endocrine disorders) , ≥ grade 3 infusion-related reactions, and systemic allergic reactions/serious type I hypersensitivity are considered AESIs, and are required to be reported as soon as possible in accordance with this study (only to the sponsor according to the procedures described in Section 9.2).

<u>Immune-related Adverse Event</u>

An irAE is defined as an AE which is associated with drug exposure and consistent with an immune-mediated mechanism of action, with no clear alternative etiology. Serologic, immunologic, and histological (biopsy) data should be used to support the diagnosis of irAEs, as appropriate. Appropriate measures should be taken to exclude tumors, infection, metabolism, toxins, or other causes for irAEs.

Based on the mechanism of action of AK105 leading to T cell activation and proliferation, it is possible to observe irAEs during this study. Potential irAEs, including immune-mediated colitis, dermatitis, pneumonia, hepatitis, encephalitis, nephritis, and endocrine disorders, may

be similar to those with anti-PD-1/L1 inhibitor.

Patients should be monitored for signs and symptoms of irAEs. In the absence of an alternative etiology (e.g., infection or PD), an immune-related etiology associated with clinical signs or symptoms of colitis, dermatitis, pneumonia, hepatitis, encephalitis, nephritis, and endocrine disorders should be considered. Guidelines for the management of subjects with irAEs are described in Section 3.1.3.

9.1.1.4. Severity of AE

All AEs are graded according to NCI CTCAE Version 4.03 (from Grade 1 to 5, as shown below).

- Grade 1, mild AE
- Grade 2, moderate AE
- Grade 3, severe AE
- Grade 4, life-threatening or disabling AE
- Grade 5, AE-related death

Severity vs Seriousness: Severity is used to describe the intensity of a particular event, which itself may be of relatively minor medical significance (e.g., severe headache). The difference is that "seriousness" is based on the outcome of the subject/event at the time of the event. For example, NCI CTCAE Grade 4 (life-threatening or disabling AEs) is assessed based on clinical descriptions specific to the severity of each AE, and these criteria may differ from those used to assess the seriousness of the AE. AEs assessed as Grade 4 based on NCI CTCAE grade may not be serious based on seriousness assessment.

9.1.1.5. Causality Assessment

Based on the criteria for causality assessment of the drug and adverse events, the causality between adverse events and the tested drug will be categorized into five levels, i.e., definitely related, possibly related, unlikely related, not related and not determined. Of these, definitely related, possibly related and not determined are assessed as adverse drug reactions. Refer to the table below for specific judgments. The relationship between an AE and AK105 will be determined by the Investigator on the basis of clinical judgment and the following definitions:

Table 12 Criteria for causality determination of adverse drug reactions

Grading	Judgment criteria
Definitely related	The event occurs in a reasonable temporal relationship after administration, and is consistent with the known reaction type of suspected study drug; the event is improved after drug withdrawal but reappears after re-administration.
Possibly related	The event occurs in a reasonable temporal relationship after administration, and is not consistent with the known reaction type of suspected study drug; the event may be caused by the subject's clinical status or other treatments.
Unlikely related	The event does not occur in a reasonable chronological order after administration, and the event is not consistent with the known type of reactions of the suspected investigation product; the subject's clinical status or other treatment regimens may also contribute to the event.
Not related	The event occurs in an unreasonable temporal relationship after administration, and is not consistent with the known reaction type of suspected study drug; the event may be caused by the subject's clinical status or other treatments; the event is resolved when the disease state is improved or the other treatments are discontinued, but reappears when the other treatments are resumed.
Not determined	The occurrence of event has no clear relationship with the time sequence after administration and is similar to the known reaction type of the investigational product, and other drugs used concomitantly may also have caused the corresponding event.

When an SAE occurs, the investigator' s initial report to the sponsor/CRO safety team may contain very little information. However, it is important for the investigator to assess the causality of each event before sending the SAE CRF to the sponsor/CRO safety team. The investigator may modify his/her opinion on causality based on subsequent information and modify the SAE CRF accordingly. The causality assessment is one of the criteria used when determining regulatory reporting requirements.

9.1.1.6. Actions Taken with Study Drug

- 1 = None: There is no change in the administration dose of study drug.
- 2 = Permanent discontinuation: Permanently discontinue the study drug.
- 3 = Dose reduction: Reduce the dose of study drug. (This study is not applicable)
- 4 = Interruption: Temporarily discontinue the study drug .
- 5 = Not applicable

9.1.1.7. Outcomes of Adverse Events

- 1 = Recovered/resolved
  - The subject fully recovers from the AE and no sequelaes are observed.

- 2 = Recovered/resolved with sequelae
  - AE is ongoing with sequelaes.
  - With definite sequelae/residual effects.
- 3 = Not recovered/not resolved
  - The AE itself is ongoing and still observable.
- 4 = Fatal.
- 5 = Unknown.

9.1.1.8. Other Actions Taken for the Event

- 1 = None.
  - No treatment required.
- 2 = Medication required.
  - Prescription and/or over-the-counter medication is required for this AE.
- 3 = Hospitalization or prolongation of hospitalization required.
  - Hospitalization or prolongation of hospitalization is required due to AE (regardless of need for medication).
- 4 = Other.

9.1.2. Recording of Adverse Events

The investigator is required to fully document any adverse event, including diagnosis (if no diagnosis, symptoms, signs, including laboratory abnormalities should be recorded), start and end date and time (if applicable), CTCAE severity grades and changes, serious adverse events or not, adverse events of special interest or not, action taken with study drug, treatment given due to AEs, outcome of the event, and relationship of adverse event to study drug.

For serious adverse events, the investigator should also provide the date of AE that met the criteria for an SAE, the date of SAE that the investigator is informed, the rationale for the AE being an SAE, date of hospitalization, date of discharge, possible cause of death, date of death, whether an autopsy is performed, causality assessment with the study procedure, causality assessment with other drugs, and other possible cause of SAEs. The investigator should also provide the rationale for judging the relevance and the description of SAEs. In the description of SAEs, it is also necessary to include the subject's number, age, gender, height and weight; the indication for treatment with the investigational drug and disease stage of the subject and relevant systemic conditions; and the clinical course such as the occurrence, development, outcome and consequence of the SAE; laboratory findings related to SAEs (test time, units and normal range must be provided); SAE-related past history, concomitant diseases, occurrence and duration, etc.; SAE-related medication history, concomitant drugs and their start, duration of treatment, dosage and administration, etc.; details of initiation, duration,

dosage and administration of the investigational product.

Items regarding the recording of AEs are described as follows:

Diagnosis, Symptoms and Signs

If a diagnosis has been made, the diagnosis should be recorded on the CRF rather than the individual symptoms and signs (for example, liver failure should be recorded, rather than jaundice, elevated transaminase, and asterixis). If it is not determined that symptoms and signs are caused by the diagnosis at the time of reporting, they will be recorded as individual AE/SAE. If it is determined that symptoms and signs are caused by the diagnosis, only the diagnosis is reported individually, and symptoms and signs are included in the diagnosis. Records of symptoms and signs should be deleted for AEs, and follow-up update reports should be sent for SAEs.

Adverse Event Secondary to Other Event

In general, primary events should be recorded for adverse events secondary to other events (such as those caused by other events or those that are clinical sequelae), unless the secondary event is severe or serious adverse events. However, a secondary event with significant clinical relevance should be recorded as an independent adverse event in the CRF if its time of occurrence is different from the primary event. If the association between events is unclear, the event should be recorded separately in the CRF.

Persistent or Recurring Adverse Events

Persistent adverse events refer to adverse events that persist and have not been alleviated between two time points of the subject. Such adverse events should be recorded only once on the CRF. The initial severity of the event should be recorded and then updated in case the event is aggravated to record the highest severity of the event.

Recurrence of adverse events refers to the adverse events that have been alleviated between two time points, but have occurred later. The occurrence of the event should be recorded separately in the CRF.

Laboratory Test abnormality

Laboratory abnormalities with clinical significance should be reported as AEs. It is the responsibility of the investigator to review all laboratory abnormalities and to make medical judgment on whether each laboratory abnormality should be reported as an AE.

Death

All deaths occurring during the entire study, including that during the 90-day follow-up period after the last dose, whether related to the study drug or not, should be recorded in the death report form in the CRF and reported to the sponsor as SAEs in a timely manner.

When recording an event of death, the cause of death will be recorded as an adverse event if the cause of death is clear, and the outcome of the adverse event will be recorded as death and

the event will be reported as an SAE; if the cause of death is unknown at the time of reporting, it should be recorded as "unexplained death" in the AE form of the CRF, and be reported as an SAE of "unexplained death" before further investigation of its exact cause.

If the death is clearly caused by tumor progression, it should be recorded on the death report form of the CRF and be notified to the sponsor in a promptly manner, by referring to the general SAEs reporting timelines and requirements for recording and reporting.

Pre-existing Medical Condition

Symptoms/signs that have been present in the screening period of the study should be recorded and reported as adverse events only if there is an increase in severity, frequency, and nature (except for the deterioration of the disease condition under study). Changes relative to pre-existing conditions, such as "increase in headache frequency", should be reflected in the record.

Hospitalization, Prolongation of Hospitalization, or Surgery

Any adverse events that result in hospitalization or prolongation of existing hospitalization should be recorded and reported as SAE, with the following exceptions:

- Planned hospitalization or prolongation of hospitalization per protocol requirements (e.g. for dosing, response evaluation, etc.)
- Hospitalization due to unchanged pre-existing medical conditions prior to study participation. For example, elective surgery/treatment that has been scheduled prior to study participation.

However, if the pre-existing disease has been deteriorated in the study (e.g requires surgery/treatment earlier than originally planned), elective surgery/treatment that is required due to the deterioration of the disease will be considered an adverse event.

Progressive Disease

Progressive disease is defined as worsening of the subject's condition due to the primary tumor to which the investigational drug is directed, the appearance of a new lesion relative to the primary tumor, or progression of an existing lesion is considered progressive disease. In general, disease progression is not recorded as an AE or reported as an SAE, and death due to symptoms and signs of disease progression that occur within 90 days of the last dose should be reported with reference to the SAEs.

9.2. Serious Adverse Event Reporting-Investigator Process

9.2.1. Initial Report

The SAE should be reported in accordance with regulatory requirements:

- The investigator shall fill in the "Collection Form for Serious Adverse Events" provided by the sponsor, sign and date it, report it to the sponsor or its representative by mail within 24 hours, and report it to the Ethics Committee in time according to the requirements of GCP; specific contact information for reporting is listed in the

Investigator File provided to each site. The original SAE report form and fax confirmation form should be kept at the study site along with the CRF file.

- If the investigator does not have all information regarding an SAE, he/she will not to wait to receive additional information before notifying the sponsor or the designee and completing the form. The form will be updated when additional information is received.
- The investigator should provide other information (autopsy report, medical records of termination phase, and other necessary information) on SAEs (including reported deaths or adverse drug reactions) as required by the sponsor, medical institution, and ethics committee.
- The sponsor should promptly scrutinize the SAEs with the investigator, take necessary measures to ensure the safety, rights and interests of the subjects, report them in a timely manner to drug regulatory authorities, and notify other investigators of clinical studies involving the same drug.
- All SAEs should be followed up as far as possible until resolution or improvement to baseline, subject death, loss of contact, or final confirmation by the investigator that the SAE is not related to study treatment.

All SAEs from the signing of the ICF up to 90 days after the last dose of study drug are to be documented and reported during the study. Investigators are required to report all SAEs related to study treatment regardless of whether they occur after the study. In addition, if there are unresolved AEs or abnormal laboratory results considered related to study treatment, the subjects will continue to be followed up until resolution or return to baseline status. SAEs occurring outside the above-mentioned period, if considered to be related with the study drug, should also be reported to the sponsor.

9.2.2. Follow-up Report

Follow-up information should be filled in a new SAE report form, and be identified as a follow-up report.

Upon receipt of new information on the reported SAE, the follow-up report is uploaded within the specified time and the subsequent reporting process is the same as the initial reporting process specified in Section 9.2.

9.2.3. Notifying Investigators or Ethics Committee/Institutional Review Board

In accordance with the Good Clinical Practice (2020 Version), the sponsor should report SUSARs to all investigators participating in clinical trials, clinical trial institutions and the Ethics Committee; and the sponsor should report suspected and unexpected serious adverse reactions to the drug regulatory authorities and the National Health and Family Planning Commission (NHC) of the People's Republic of China.

For study drug-related SUSARs that are fatal or life-threatening, the sponsor should submit them to the drug regulatory authorities (CDE) and the Health Administration (Health Commission) within 7 days of being first informed, and distribute them to all investigators

participating in the clinical trials, clinical trial institutions, and ethics committees. Follow-up reports will be submitted to the CDE and the Health Commission within the next 8 days and distributed simultaneously to all investigators, clinical trial facilities, and ethics committees.

For study drug-related SUSARs that are not fatal or life-threatening, the sponsor should submit them to CDE and the Health Commission within 15 days of being first informed and distribute simultaneously to all investigators, clinical trial institutions, and ethics committees.

9.3. Other Events Requiring Immediate Reporting

9.3.1. Overdose

An overdose is defined as the dose of study drug received by the subject exceeds 20% of the dose specified in the protocol.

- AE-related overdose is recorded as AE diagnosis/symptoms on the respective AE module and the overdose CRF module in the CRF, or by using the corresponding recorded overdose report form.
- Asymptomatic overdose events are reported only on the overdose CRF module or by using the corresponding recorded overdose report form.

All overdose with and without AEs must be reported electronically or in writing to the sponsor/CRO by the investigator or other personnel in the study site (when this person becomes aware of the event) via EDC within the specified time. For applicable timeline for reporting of SAE-related overdose events, please refer to Section 9.2.

9.3.2. Combined Elevations of Transaminases and Bilirubin

Concurrent elevation of aminotransferases and bilirubin, whether serious or nonserious, or causally related, if in accordance with the underlying Hy's law (ALT or AST $\geq 3 \times$ ULN and TBil $\geq 2 \times$ ULN), should be reported to the sponsor/CRO via EDC or SAE in written format with the investigator's judgment and detailed description of seriousness and causality. Elevation of transaminase should be consistent with elevation of TBil (i.e., on the same day) for potential conditions that meet Hy's Law, but there is no specific time range for the elevation of transaminases and TBil. These events should be reported as soon as possible in accordance with the SAE reporting process specified in Section 9.2. The site will promptly initiate follow-up investigations, laboratory tests, detailed medical history collection and inquiries to determine if the results are reproducible and/or if there is objective evidence that clearly supports the results are caused by disease (e.g., gallstones and bile duct obstruction with gallbladder dilatation) or other medications other than study drug. Further testing may include testing for acute hepatitis A, B, C, and E and imaging of liver.

9.3.3. Other Adverse Events of Special Interest

AEs of immune-related reaction $\geq$ Grade 3, infusion-related reaction $\geq$ Grade 3, and systemic anaphylaxis/serious type I hypersensitivity (as defined in Section 9.1.1.3) are required to be reported per the requirements in Section 9.2.

9.3.4. Reporting and Follow-up of Pregnancy during Clinical Study

Any subject who becomes pregnant while on study drug or within 120 days of discontinuation of study drug must notify the sponsor or Drug Safety Associate of the CRO. Although pregnancy is not technically an AE, all pregnancy events must be tracked to determine their outcome. This information is important for both drug safety and public health issues. If any subject becomes pregnant during the study, the investigator or designee is obligated to report the in-process event using the Pregnancy Report Form. The investigator should make every effort to follow up the subject until the end of pregnancy. If the outcome of the pregnancy meets the criteria for immediate classification as an SAE (i.e., postpartum complication, spontaneous abortion, stillbirth, neonatal death or congenital anomaly, including abnormalities of aborted fetuses), the investigator should follow the procedures for reporting an SAE. If a female subject becomes pregnant during the clinical trial, the subject will be excluded from the study. When the investigator becomes aware that the spouse of a male subject is pregnant, the investigator must follow up the pregnancy as much as possible. The investigator should promptly report all pregnancies to the sponsor.

9.4. Clinical Laboratory Evaluation

Safety laboratory assessments will include hematology test, blood chemistry test, coagulation test, thyroid function tests, routine urinalysis, pregnancy tests, etc. Refer to Section 6 for test schedule.

- Coagulation: Prothrombin time (PT), international normalized ratio (INR), and activated partial thromboplastin time (APTT, at screening only).
- Hematology: Including hemoglobin (Hb), packed cell volume (PCV), white blood cell differential count (WBC, including absolute count), red blood cell (RBC), blood platelets count (BPC), mean corpuscular hemoglobin concentration (MCHC) and mean corpuscular volume (MCV).
- Blood chemistry: Calcium, phosphorus, magnesium, albumin (ALB), glucose (GLU), serum creatinine (Cr), uric acid (UA), total protein (TP), blood urea nitrogen (BUN) or urea (Urea), aspartate aminotransferase (AST), alanine aminotransferase (ALT), alkaline phosphate enzymes (ALP), total bilirubin (TBil) and direct bilirubin (DBil), glutamyltransferase (GGT), lactate dehydrogenase (LDH), sodium, potassium, chloride, triglycerides, cholesterol, and serum C-reactive protein (CRP).

  (Note: For blood chemistry: tests for AST, ALT, ALP, and TBil must be performed concurrently and evaluated concurrently.)
- Thyroid function test: TSH, free T4 and free T3
- Routine Urinalysis: Including acidity and alkalinity, protein, specific gravity, glucose, bilirubin, ketones, occult blood and microscopic examination (including, if necessary, white blood cells and red blood cells in urine)
- Serum β-hCG pregnancy test or urine hCG pregnancy test
- Hepatitis B virus screening includes HBsAg, HBsAb, and HBcAb (HBV DNA by hepatitis B virus DNA load test if necessary) and hepatitis C antibody (qualitative

testing for hepatitis C RNA if antibody test is positive) (at screening only)

- Human immunodeficiency virus antibody (HIV-Ab) (at screening only)

The investigator must assess the clinical significance of all laboratory findings. All abnormal laboratory results considered clinically significant by the investigator should be recorded on the AE page of the CRF. If the abnormal laboratory value constitutes an SAE, the SAE should be reported through EDC or written SAE form and other relevant procedures must be followed (see Section 9.2). Abnormal laboratory test results (NCI CTCAE Grade 3 or 4) that occur during clinical studies should be followed up until the repeat test results return to normal (baseline), stabilize, or are no longer clinically significant.

9.5. Vital Signs

Vital sign including blood pressure, pulse rate, respiratory rate, and temperature will be measured according to the visit schedule in Section 6. During treatment, vital signs will be measured within 30 min before starting AK105 administration, 30 min (± 10 min) within 1 h of infusion, (+ 10 min) at the end of infusion, 30 min (± 10 min) and 60 min (± 10 min) after the end of infusion. On Day 1 of Cycle 1, additional vital sign measurements will be performed 3 h (± 15 min) after the first infusion. For subsequent dosing, a 3-h observation period is not required unless the subject experiences an infusion-related reaction. If a subject experiences an infusion-related reaction, additional vital sign measurements will be performed 3 h after infusion and 3 h after each subsequent infusion. Additional monitoring and assessment of vital signs will be performed at the discretion of the investigator in accordance with clinical routine practice.

9.6. Electrocardiogram

A 12-lead ECG will be recorded for all subjects at the scheduled visit described in Section 6. All ECGs will be reviewed by the principal investigator or designated physician. If any clinically significant condition is observed on the ECG, it will be recorded as an AE by the investigator on the CRF, which represents a change from baseline in the result. In the presence of clinically significant ECG, abnormalities include ECG showing QTcF value > 500 msec and two additional 12-lead ECGs should be obtained over a short period of time (e.g., 30 min). The original ECG tracing results for each 12-lead ECG will be saved together with the subject’s original file.

9.7. Physical Examination

Physical examinations will be performed according to the schedule in Section 6. A complete physical examination including general condition, skin, mucosa, superficial lymph nodes throughout the body, head and organs, neck, chest, abdomen, rectoanal and external genitalia, spine and extremities, nervous system, others will be performed at screening and follow-up at the end of treatment (Day 30 after the last dose). A targeted physical examination based on symptoms should be performed during treatment and prior to study drug administration.

9.8. ECOG Performance Status

ECOG performance status will be assessed at screening and then at each scheduled follow-up visit throughout the study (see Section 6). ECOG performance status will be assessed at the time specified in the Assessment Schedule based on the following content:

Performance Status Score	Performance Status
0	Fully active, able to carry on all pre-disease performance without restriction.
1	Restricted in physically strenuous activity but ambulatory and able to carry out work of a light or sedentary nature, e.g., light house work, office work.
2	Ambulatory and capable of all self-care but unable to carry out any work activities. Up and about more than 50% of waking hours.
3	Capable of only limited self-care, confined to bed or chair more than 50% of waking hours.
4	Completely disabled; cannot carry on any self-care; totally confined to bed or chair.
5	Death

10. OTHER ASSESSMENTS

Not applicable.

11. STATISTICAL METHOD

11.1. Analysis Set

11.1.1. Enrollment Analysis Set

The Enrollment Analysis Set includes all subjects who have signed the ICF and been enrolled into the study.

11.1.2. Full Analysis Set

The Full Analysis Set (FAS) includes all subjects in the Enrollment Analysis Set who have received at least one dose of AK105 at any dose and met the following inclusion criteria:

- Subjects diagnosed with disease stage of IVb in accordance with Chinese Nasopharyngeal Carcinoma Staging 2017 (2008 Expert Consensus on Nasopharyngeal Cancer Staging Revision) at enrollment
- Failure of prior first-line platinum-containing chemotherapy (monotherapy or concomitant medication) and second-line monotherapy or concomitant medication. Treatment failure is defined as disease progression occurring during or after chemotherapy. For subjects who have received prior neoadjuvant chemotherapy, concurrent chemoradiotherapy, or adjuvant chemotherapy, the original treatment regimen should be the first-line regimen if recurrence/metastasis occurs within 6 months after the end of the last treatment. All treatment changes due to drug intolerance are not considered as treatment failure
- At least one measurable lesion. If used for the data analysis of IRRC assessment, the presence or absence of measurable lesion should be determined based on the baseline assessed by IRRC (defined per RECIST v1.1).

11.1.3. Safety Analysis Set

The Safety Analysis Set (SS) includes all enrolled subjects who have received at least one dose of the study drug. The analysis is conducted according to the treatment actually received by the subjects.

11.2. General Statistical Considerations

Appropriate descriptive statistics will be performed for selected efficacy endpoints related to population, safety, PK, immunogenicity and anti-tumor activity. Descriptive statistics for continuous data includes mean, median, standard deviation, and range, and categorical data are summarized using frequency counts and percentages at the same time. Data summaries may be present in graphs.

Changes from baseline to post-treatment or ratios of post-treatment to baseline are assessed only for those subjects who have both baseline and post-treatment measurements. Unless otherwise specified, the last non-missing value for a variable collected before the first dose of study drug will be used as the baseline value. Missing or out-of-control data are generally not

included in data analysis.

Safety analysis will be performed based on the Safety Analysis Set and anti-tumor activity-related efficacy endpoints will be analyzed on the basis of the FAS. Individual efficacy data will be presented in listings and/or graphical formats.

A detailed Statistical Analysis Plan (SAP) will be prepared after the first subject is enrolled and finalized prior to database lock. The SAP will provide the full content of the analysis and the description of the results. The statistical methods described in the SAP may change with the progress of the study. All statistical analyses will be performed using SAS 9.2 (or higher).

11.3. Study Population Data

For subjects in the Enrollment Analysis Set, the reason for discontinuation of treatment and study will be summarized and listed.

Demographic and baseline characteristics such as age, sex, baseline ECOG performance status, prior therapy methods, and best response to prior therapy will be summarized for the FAS and the Safety Analysis Set. If 2 analysis sets are identical in the study, only one of them will be present in the table.

Study drug exposure, treatment duration, and compliance with study treatment will be summarized for the safety analysis set using descriptive statistics.

11.4. Efficacy Analysis

The following efficacy endpoints will be derived. Please refer to SAP for details.

- ORR is defined as the proportion of subjects achieving a best overall response (BOR) of confirmed CR or PR per RECIST v1.1. Best overall response is defined as the best response recorded from the start of treatment until progressive disease (sequentially CR, PR, SD, PD, or not evaluable), or best response recorded (from the start of treatment to assessment of the last evaluable tumor imaging) if PD is not achieved prior to initiation of subsequent anti-cancer therapy or study discontinuation (whichever occurs first). The best overall response requires confirmation if it is CR or PR, which is defined as confirmation of CR or PR response by repeated imaging assessments performed not less than 28 days (4 weeks) after the first observation of a CR or PR response, and there is no evidence of PD between the follow-up of the first observed CR or PR and the follow-up for confirmation of CR or PR. Patients with no post-baseline response assessment (for any reason) will be considered non-responders for BOR.

- Duration of response (DoR) is defined as the period from the first documentation of confirmed response (CR or PR) to the first documentation of progressive disease (as per RECIST v1.1) or death due to any cause (whichever occurs first). Subjects who are alive and have no progressive disease at the time of analysis data cutoff will be censored on the date of last tumor determination. DoR will be evaluated only for subjects who have a confirmed CR or PR.

- Disease control rate (DCR) is defined as the proportion of subjects achieving a best overall response of confirmed CR or PR or SD per RECIST v1.1. Disease control

rates at week 16 and week 24 are defined as the proportion of subjects achieving a best overall response of confirmed CR, PR or SD for at least 16 weeks and for at least 24 weeks respectively.

- Progression-free survival (PFS) is defined as the period from the start of treatment until documentation of progressive disease (per RECIST v1.1) or death due to any cause (whichever occurs first). Subjects who are alive and have no progressive disease at the time of analysis data cutoff will be censored on the date of last tumor assessment. Subjects without post-baseline assessments will be censored on the date of first dose. The 6-month PFS (6M PFS%) is defined as the proportion of subjects who have no progressive disease within 6 months after the first dose.
- Overall survival (OS) is defined as the period from the start of treatment until death due to any cause. Subjects who are alive at the time of analysis data cutoff will be censored on the last known survival date. Subjects who do not provide any follow-up information will be censored on the day of enrollment. The 1-year survival rate (1YR OS%) is defined as the proportion of subjects alive within 1 year after the first dose.

The primary endpoint of this study is ORR assessed by IRRC per RECIST v1.1 in the FAS population. The analysis of the primary endpoint is performed after the last subject has completed at least 24 weeks of follow-up (an interval between the data cutoff date and the first dose date ≥ 24 weeks) (this follow-up assessment is not required if the subject has achieved radiographic disease progression before 24 weeks).

ORR, DCR, and PFS will be analyzed based on the FAS. The two-sided 95% confidence interval (CI) for ORR and DCR is estimated with the Clopper-Pearson method. Time to event endpoints (DoR, PFS, and OS) will be analyzed using the Kaplan-Meier method. The graphical analysis will include a spider gram of the tumor burden percentage of the target lesion changing from baseline over time, and a waterfall gram of the optimum percentage of the target lesion tumor burden changing from baseline.

11.5. Pharmacokinetic/Immunogenicity Analysis

11.5.1. Pharmacokinetic Analysis

Individual AK105 concentrations will be tabulated by descriptive statistics. The relevant descriptive statistics of the PK parameters of the non-compartmental model may include AUC, C_{max}, CL, $t_{1/2}$, etc.

A population PK model will be established in this trial. The impact of physiological relevant subjects characteristics (covariates) on PK will be evaluated. The relationship between exposure and response or safety will be evaluated.

11.5.2. Immunogenicity Analysis

The immunogenic potential of AK105 will be assessed by summarizing the number and percentage of subjects who develop detectable ADA. The impact of ADA on PK will be assessed if data is available. Samples will be collected for evaluating the neutralizing capacity of ADAs in the future. Only subjects who have received at least one dose of AK105 and provided a baseline sample and at least one post-treatment sample will be evaluated.

11.6. Safety Analysis

Safety analysis will be performed using the safety analysis set according to the treatment actually received by the subjects. Safety analysis usually summarizes descriptive information appropriately in a table by dose group.

11.6.1. Adverse Events Analysis

Treatment-emergent adverse events (TEAE) is defined as an AE that occurs during treatment (from the date of first dose up to 90 days after the last dose date) but does not occur before treatment; or an AE that reoccurs during the treatment period and is present at baseline but stops prior to treatment; or a persistent AE with severe deterioration after treatment relative to that before treatment.

AEs will be coded using the Medical Dictionary for Regulatory Activities (MedDRA) and graded according to NCI CTCAE Version 4.03. The number and percentage of subjects reporting TEAEs will be tubulated by worst CTCAE grade, system organ class, and preferred term.

The number and percentage of subjects reporting treatment-emergent SAEs and TEAEs/SAEs considered related to AK105 will also be tabulated in the same manner.

Listings by subject AE (including TEAE) data should include, but not limited to, verbatim terms, preferred terms, system organ class, CTCAE grade, and relationship to study drug.

Deaths, other SAEs, and other significant AEs including those that lead to permanent discontinuation of AK105 will be listed.

11.6.2. Clinical Laboratory Evaluation Analysis

Clinical laboratory results and changes from baseline will be summarized using descriptive statistics by scheduled evaluation time, including follow-up at the end of treatment and the maximum and minimum values after baseline.

Laboratory abnormalities are graded according to NCI CTCAE version 4.03. A two-way frequency table of CTCAE grade at baseline and the worst grade after baseline will be shown in the shift table of clinical laboratory tests. Abnormal clinical laboratory test results of CTCAE Grade 3 or 4 will be listed.

11.6.3. Vital Signs Analysis

Clinical sign measurements and changes from baseline will be summarized using descriptive statistics by scheduled evaluation time, including follow-up at the end of treatment and maximum and minimum values after baseline.

11.6.4. Electrocardiogram Analysis

ECG parameters (PR, RR, QRS, QT, QT, QTcB, and QTcF) are summarized using descriptive statistics by planned evaluation time for actual values and changes from baseline, including follow-up at the end of treatment and maximum value after baseline. QTcF is recognized as

the primary correction method for assessing cardiac safety in patients.

The incidence of outliers of maximum absolute QTcF and QTcB intervals (new value > 450 ms, new value > 480 ms, new value > 500 ms) and maximum absolute uncorrected QT intervals (new value > 500 ms) in all post-baseline evaluations and the incidence of outliers of maximum changes of QTcF and QTcB (>30 and >60 ms) from baseline in all post-baseline evaluations will be summarized. A "new value" is defined as a QTc abnormal value that does not exist at baseline but occurs at least once in post-baseline ECG assessments.

11.7. Biomarker Analysis

In this study, the cut point of TPS for PD-L1 expression is pre-specified as 50% until the data for subject's individual PD-L1 expression are available (blinded to the individual PD-L1 data). Based on this cut-off value and PD-L1 expression at baseline, TPS ≥ 50% is defined as the positive group and < 50% as the negative group. In this study, immunohistochemistry is used to detect PD-L1 expression using SAB028 antibody. As of May 25, 2020, a total of 132 subjects (including subjects who failed screening) were tested, and the incidence of TPS ≥ 50% for PD-L1 expression was 42% (56/132). The TPS cut-off value is selected based on the incidences of patients with PD-L1-positive nasopharyngeal carcinoma at the selected cut-off value provided by the testing unit and the incidence of patients with PD-L1-positive nasopharyngeal carcinoma reported in the literature (Ma BBY, 2018), which is basically consistent. The analysis of ORR, DCR, and PFS will be performed in the PD-L1 subgroup (positive and negative) based on the FAS, respectively.

Investigators and subjects are blinded to individual PD-L1 data until the end of the study. The sponsor will be blinded to individual PD-L1 data prior to cleaning and locking of efficacy data.

The relationship between PD-L1 at baseline expression (not limited to pre-specified cut point) and the assessment of AK105 anti-tumor activity will be explored. The relationship between EBV-DNA expression at baseline, EBV-DNA expression with post-dose change from baseline and the anti-tumor activity of AK105 will be explored, respectively. The analytical procedures are described in the Statistical Analysis Plan (SAP).

11.8. Interim Analysis

In this study, an interim analysis may be planned after at least 110 subjects have been enrolled and completed at least 16 weeks of follow-up (an interval between the data cutoff date and the date of first dose ≥ 16 weeks), and the results of the interim analysis will likely be used to communicate with CDE. Efficacy analysis will be performed primarily based on the FAS with at least 16-week follow-up (at least 2 response assessments at the time of data cutoff). Safety analyses will be performed based on the Safety Analysis Set. The sponsor does not plan to prematurely discontinue the study based on these efficacy or safety findings of planned analysis.

11.9. Sample Size Determination

This study is a single-arm clinical study with the primary endpoint of ORR in the FAS population. The lower limit of the 95% CI for ORR in the FAS population is not less than 15% in the efficacy analysis. If an ORR of 26% is assumed for the efficacy of AK105 in the FAS population, there is 82% power to observe at least 25 CR or PR in 110 evaluable subjects , with the lower limit of the 95% confidence interval not less than 15%. ORR and its confidence interval are estimated based on the proportion of subjects who are expected to have a CR or PR (see Table 13). Considering possible reasons such as dropouts of subjects, assuming a dropout rate of 15%, a total of approximately 130 subjects are planned to be enrolled to ensure 110 evaluable cases.

Table 13 ORR and 95% CI estimates in 110 evaluable subjects

Number (%) of subjects achieving CR or PR among 110 subjects	21(19.1)	23(20.9)	24(21.8)	25(22.7)	27(24.5)	29(26.4)
Lower limit of 95% CI	12.2%	13.7%	14.5%	15.3%	16.8%	18.4%
Upper limit of 95% CI	27.7%	29.7%	30.7%	31.7%	33.7%	35.6%

12. DATA INTEGRITY AND QUALITY ASSURANCE

The investigator/study site is required to open the direct access to the source data/documents and allow study-related monitoring, audits, IRB/IEC review and regulatory audits. Direct access includes permission to examine, analyze, verify and reproduce any records and reports that are essential to the evaluation of the clinical study.

12.1. Monitoring and Audit

The clinical monitor is responsible for conducting regular follow-up to the study site throughout the study to verify its compliance with the protocol, its compliance with the completeness, accuracy, and consistency of data and its compliance with ICH GCP and local regulations during the conduct of the clinical study. The clinical monitor is responsible for examining the CRFs and ensuring the integrity of the critical study documents and should have the right to access to the subject's medical records and other study-related records (as required when checking the contents of the CRF).

The clinical monitor is required to communicate to the investigators about their deviations from the protocol, SOPs, GCP, and applicable regulations and ensure that appropriate measures designed to prevent recurrence of the identified deviations have been taken and documented.

The investigator agrees to cooperate with the clinical monitor to ensure that any issues identified during monitoring follow-up are resolved and documented.

The selected study may be audited by representatives of the sponsor in accordance with the ICH GCP and the sponsor's audit plan. To evaluate the conduct of the study and its compliance with the protocol, ICH GCP, and applicable regulatory requirements, site facilities (e.g., pharmacies, drug storage areas, laboratories, etc.) need to be inspected and study-related records (e.g., CRFs, source data, and other relevant documents) should be reviewed.

12.2. Data Collection

All data collected during the study will be recorded in the subject's individual CRF. The sponsor or its designee will provide CRF and instructions for completing the CRF. Any modifications made to the CRF will be automatically recorded through the "audit traces" function in the EDC system.

The CRF should be kept up to date to assist the monitor in reviewing the status of subjects throughout the study. The CRF should be completed as soon as possible after follow-up, and the questions should also be answered as soon as possible. The CRF should be completed, reviewed and signed by the investigator as soon as possible after the last subject follow-up.

Each subject who has signed the ICF and passed all screening procedures should have a completed CRF. If a subject is not treated, the reason should be recorded in the CRF.

All external data (e.g. site laboratory, PK and PD data) will be integrated with those in the subject's CRF according to the data management plan.

The subject number and date of study participation, along with the study code, should be recorded in the subject's medical/study file by the investigator along with the study code. The investigator should also record the following information in the medical/study file: written and oral confirmation of informed consent, subject's clinical status, date of each study follow-up, date of administration of the study drug, concomitant medications, copies of all relevant reports and laboratory findings, comments on the results, and any AEs mentioned.

The investigator will sign and date the CRF at the designated location via the electronic signature procedure of the EDC system. These signatures indicate that the investigator has examined or reviewed the data on the CRF, data queries, and site notifications and agrees with the contents.

All information and other materials used by subjects and investigators should be described in clear and understandable terms and sentences.

12.3. Data Management

The sponsor may identify each subject in the database using unique SID as defined by the sponsor.

To ensure the quality of clinical data for all subjects and sites, a clinical data management review of the subject data will be performed. The data in the CRF will be checked electronically and manually, when the data is electronically checked using internal procedural data rules, the checks and queries established for data entry in the EDC system should be edited. Post-entry validation will be performed as described in the Data Management Plan. Queries arising from the rules and those raised by the auditors will arise within the EDC application. During this review, subject data will be checked for consistency, omissions, and any apparent discrepancies. In addition, compliance of the data with the protocol and GCP will also be reviewed. To address all issues that arise during the review of clinical data management, CRF queries will be raised and resolved within the EDC application.

Data obtained from external sources (eg, site laboratory) will be consistent with those in the clinical database. SAEs in the clinical database will be consistent with those in the safety database.

All AEs will be coded using MedDRA. Concomitant medications selected according to the reference directory of the World Health Organization Drug Dictionary (WHO-DD) and prior tumor therapies will also be recorded on the CRF.

12.4. Study Documents and Storage

The investigator will maintain a signed list of appropriately qualified persons who are responsible for the study. All persons authorized to enter any content or make modifications to the CRF are included in the signature list.

Source documents refer to original documents, data and records, the subject's CRF data are all obtained from it. These documents include, but not limited to, hospital records, clinical and

outpatient medical records, laboratory tests and pharmacy records, diaries, microfilms, x-rays, and correspondence. It is the responsibility of the investigator and study personnel to maintain a complete central file archiving system (Trial Master File) for all study-related (critical) documents for inspection at any time by representatives of the sponsor and/or applicable regulatory authorities. Critical documents include:

- Subject files, containing completed CRF, ICF and copies of supporting source documents, if available. For studies using EDC, a PDF file (file in portable document format) will be generated for each subject's data (CRFs and queries) at the end of the study, which will be stored in the Trial Master File.
- Study files, containing the protocol with all amendments, the Investigator Brochure, copies of all relevant clinical documents required before initialization of study, and all correspondences to and from the IEC/IRB and the sponsor.
- Study drug-related records, including site receipt, accountability records, final reconciliation, and corresponding correspondence.

In addition, all original documents supporting entries in the CRF must be retained and easily accessible. All critical documents will be retained by the study facility for at least 5 years or longer after completion of the study (if specified by other applicable regulations or requirements). When these documents are no longer required, it is the responsibility of the sponsor to notify the investigator/study facility. No study documents shall be destroyed until written consent is reached between the sponsor and the investigator. If the investigator wishes to assign the study records to another party or move to another location, he/she must notify the sponsor in writing of the new responsible person and/or the new location.

12.5. Records Retention

Subject records, source documents, monitoring follow-up logs, data correction forms, CRFs, study drug inventory listings, regulatory documents (eg, protocols and amendments, IRB/IEC correspondence and approvals, approved and signed ICFs, investigator agreements, clinical supply receipts, distribution and return records) and other study-related correspondence from and to the sponsor must be maintained in the appropriate study file room at the study site. Source documents include all records and observations or markers of clinical manifestations, as well as all reports and records necessary to evaluate and reconstruct clinical studies. These records will be retained in the safety file for the time required by the policy of the study facility or study site. Prior to transfer or destruction of these records, the sponsor must be notified in writing and provided with the opportunity to continue maintaining them.

13. ETHICAL AND REGULATORY REQUIREMENTS

13.1. Ethical Conduct of the Study

This study will be conducted in accordance with GCP and all applicable regulatory requirements, including, where applicable, the current version of the Declaration of Helsinki. The investigator (or sponsor, as appropriate) is responsible for ensuring that this protocol, the study site's CRF, and any other information to be presented to potential subjects (e.g., advertisements or information to support or supplement the ICF) are reviewed and approved by the appropriate IEC/IRB. The investigator agrees to allow the IEC/IRB direct access to all relevant documents. The IEC/IRB must be constituted in accordance with all applicable regulatory requirements. The sponsor will provide the investigator with relevant document(s)/data that are needed for IEC/IRB review and approval of the study. Before the study drug can be shipped to the study site, the sponsor must receive copies of the IEC/IRB approval letter, the approved ICF, and any other information that the IEC/IRB has approved for presentation to potential subjects.

If the protocol, the study site's ICF, or any other information that the IEC/IRB has approved for presentation to potential subjects is amended during the study, the investigator is responsible for ensuring the IEC/IRB reviews and approves (where applicable) these amended documents. The investigator must comply with all applicable regulatory requirements regarding the use of the amended ICF, including that subjects enrolled after the new version of the ICF has been approved by the IEC/IRB should use the new version of the ICF. Copies of the IRB/IEC approval letter of the amended ICF/other information and the approved amended ICF/other information must be forwarded to the sponsor promptly.

13.2. Confidentiality of Subjects

In accordance with GCP and local regulations, all subjects participating in the study will be kept confidential by the investigator and the sponsor. The investigator must ensure that the subject's name is not exposed. Subjects should be identified with a unique subject number designated by the sponsor in CRF or other documents submitted to the sponsor and the contract research organization (CRO). Documents not submitted to the sponsor and CRO (eg, signed ICFs) should be kept strictly by the investigator.

As required by ICH GCP guidelines, the investigator and the study facility allow direct access to the subject's original medical records for review by authorized representatives of the company, regulatory authority and the IEC or IRB in order to verify study-related processes and data. The investigator is obligated to inform the subject that the study-related records of the subject will be reviewed by the above-mentioned representative without violating the confidentiality of the subject.

13.3. Compliance

Prior to the start of the study, the protocol, subject information and ICF, Investigator's Brochure, any subject diary card or written instructions to subjects, available safety information, subject recruitment process (e.g., advertisements), payment and compensation

information available to subjects, and documentation demonstrating investigator qualification should be submitted to the IEC or IRB for ethical review and approval in accordance with local regulations. Names and versions of all reviewed documents should be confirmed in the written approval.

Changes that occur during the conduct of the study or planned analysis are documented in protocol amendments and/or SAPs.

When necessary, the investigator must submit all subsequent protocol amendments and changes in informed consent documents or changes in study sites, facilities or personnel to the IEC or IRB and/or the sponsor for approval. The investigator should notify the IEC or IRB of protocol deviations or site-emergent SAEs and other AE reports obtained from CRO in accordance with local procedures.

In accordance with local regulatory requirements, the local regulatory affairs team or designee of the sponsor is required to ensure that all legal issues are resolved and approved by the relevant regulatory authorities prior to the start of the study, and that changes to the initial protocol or other relevant study documents are not implemented until appropriate notification or approval is received from the relevant regulatory authority.

It is the responsibility of each principal investigator to provide the IRB/IEC with reports of any serious and unexpected adverse drug reactions occurring in other studies conducted with the study drug. The sponsor will provide this information to the principal investigator to ensure that the investigator can meet these reporting requirements.

According to local requirements, the sponsor will provide safety updates/reports including suspected unexpected serious adverse reactions (if relevant) to regulatory authorities, IRB/IEC and principal investigators.

13.4. Informed Consent Process

Before a subject participates in the study, it is the responsibility of the investigator to fully explain to the subject the study objectives, methods, expected benefits, and potential hazards, thereby obtaining written consent voluntarily issued by the subject or his/her legal representative (if permitted by local regulations). A legal representative is an individual or institution that, in accordance with the requirements of law, can consent to participate in a clinical study on behalf of a subject after being authorized by the subject or law. The ICF should be written in the mother tongue of the potential subject population.

Investigators should comply with applicable regulatory requirements, GCP, and ethical principles derived from the Declaration of Helsinki to obtain the written consent from subjects. The ICF and its revisions should be approved by the IEC/IRB prior to being provided to potential subjects. The investigator should ensure that each subject is informed that he/she is free to voluntarily discontinue the study at any time and that each subject has the opportunity to ask questions and have time to consider the information provided.

The ICF should be obtained prior to the subject's participation in the study and will be kept in

the form of documents at the study site. The ICF should be signed and dated by the subject or his/her legal representative (if permitted by local regulations), and by the investigator. The original signed ICF should be kept at the study site and a copy of the signed ICF should be provided to the subject or his/her legal representative. The date on which the ICF is obtained should be documented in the medical record and CRF.

If the subject or his/her legal representative (if permitted by local regulations) is unable to read, an impartial witness should be present throughout the discussion of the informed consent. The witness should sign the ICF after the subject or his/her legal representative agrees to participate in the study and signs the ICF (if possible). Signing the ICF indicates that the witness confirms that the information in the ICF and any other written information has been fully explained to the subject or his/her legal representative, that they have understood this information clearly and that the subject or his/her legal representative have agreed to sign the written informed consent.

The ICF approved by the IRB/IEC should describe any incentive measures for subjects participating in the study and the protective measures against possible injury to the subject due to participation in the study.

13.5. Changes to the Protocol and Informed Consent Form

The sponsor will communicate to the investigator any amendments to the protocol, all of which require approval from the IRB/IEC. Protocol amendments will not be implemented until IRB/IEC approval and national regulatory approval (if applicable) are available, except that changes must be implemented immediately for the safety of subjects. If the change must be implemented immediately, the details must be documented and reported to the IRB/IEC within 5 working days. The sponsor should ensure that protocol amendments are submitted to regulatory authorities in a timely manner.

If a protocol amendment requires a change to the ICF of the study site, the amended ICF document needs to be approved by the sponsor and the IRB/IEC of the study site prior to use.

14. FINANCING AND INSURANCE

14.1. Finance

Prior to initiation of the study, the principal investigator and/or the study facility will enter into a clinical study agreement with the sponsor. The agreement will include financial information agreed upon between the parties.

14.2. Reimbursement, Compensation and Insurance

Reimbursement, compensation and insurance agreements shall be signed separately in the terms agreed upon between the parties.

15. PUBLICATION POLICY

The investigator should keep the information and data related to this trial confidential and consult with the sponsor in advance and obtain the written consent of the sponsor in order to publish any conclusions drawn from the trial. In order to protect its rights and interests, the sponsor may require the investigator not to publish relevant information until the investigational product is approved for marketing. The sponsor has the right to issue or publish information or data related to this study, or submit them to the drug regulatory authority.

16. REFERENCES

Boutros C, Tarhini A, Routier E, et al. Safety profiles of anti-CTLA4 and anti-PD-1 antibodies alone and in combination. Nat Rev Clin Oncol.201613(8):473-86.

Brahmer JR, Lacchetti C, Schneider BJ, et al.Management of Immune-Related Adverse Events in Patients Treated With Immune Checkpoint Inhibitor Therapy: American Society of Clinical Oncology Clinical Practice Guideline. J Clin Oncol 2018 Feb 14:JCO2017776385.

Chan A, Hsu MM, Goh BC, et al. Multicenter, Phase II study of cetuximab in combination with carboplatin in patients with recurrent or metastatic nasopharyngeal carcinoma. J Clin Oncol,2005.23(15): p.3568-76.

Chen, L. Co-inhibitory molecules of the B7-CD28 family in the control of T-cell immunity. Nat. Rev. Immunol.2004(4):336-347.

Cockcroft DW, Gault MH. Prediction of creatinine clearance from serum creatinine. Nephron 1976(16):31-41.

Dahan R, Sega E, Engerlhardt J, et al. FcγRs Modulate the Anti-tumor Activity of Antibodies Targeting the PD-1/PD-L1 Axis. Cancer Cell.2015 Sep 14;28(3):285-95.

Eisenhauer EA, Therasse P, Bogaerts J, et al. New response evaluation criteria in solid tumours: revised RECIST guidelines (version 1.1). Eur J Cancer.2009;45(2):228-47.

Escudier B, Faivre S, Van Cutsem E, et al. A Phase II Multicentre, Open-Label, Proof-of-Concept Study of Tasquinimod in Hepatocellular, Ovarian, Renal Cell, and Gastric Cancers. Target Oncol.2017 Aug 10.

Hsu C, Se-Hoon L, et al. Safety and antitumor activity of Pembrolizumab in Patients with Programmed Death-Ligand 1- Positive Nasopharyngeal Carcinoma: Result of the KEYNOTE-028 Study. J Clin Onco 35:4050-4056

Iwai Y, Ishida M, Tanaka Y, et al. Involvement of PD-L1 on tumor cells in the escape from host immune system and tumor immunotherapy by PD-L1 blockade. N.Proc Natl Acad Sci U S A.2002;99(19):12293-7.

Lim WT, et al, A Phase II study of pazopanib in Asian patients with recurrent/metastatic nasopharyngeal carcinoma. Clin Cancer Res,2011.17(16): p.5481-9.

Ma BBY, Lim WT, Goh BC, et al. Antitumor activity of nivolumab in recurrent and metastatic

nasopharyngeal carcinoma: an international, multicenter study of the mayo clinic phase 2 consortium

(NCI-9742). J Clin Oncol. doi:10.1200/JCO.2017.77.0388.

Nakae S, Suto H, Iikura M, et al. Mast cells enhance T cell activation: importance of mast cell costimulatory molecules and secreted TNF. J. Immunol.(2006)176,2238-2248.

Ngan Rk，Yiu HH，Lau WH, et al: Combination gemcitabine and cisplatin chemotherapy for metastatic or recurrent nasopharyngeal carcinoma: Report of a phase II study. Ann Oncol 13:1252-1258,2002

Nishino M, Giobbie-Hurder A, Gargano M, et al. Developing a common language for tumor response to immunotherapy: immune-related response criteria using unidimensional measurements. Clin Cancer Res.2013;19:3936-43.

Okazaki, T, Honjo, T. The PD-1-PD-L pathway in immunological tolerance. Trends Immunol.2006(27),195-201.

Schreiner B, Mitsdoerffer M, Kieseier BC, Chen L, Hartung HP, Weller M, Wiendl H. Interferon-beta enhances monocyte and dendritic cell expression of B7-H1(PD-L1), a strong inhibitor of autologous T-cell activation: relevance for the immune modulatory effect in multiple sclerosis. J Neuroimmunol.2004;155:172-82.

Sharpe AH, Wherry EJ, Ahmed R, Freeman GJ. The function of programmed cell death 1 andits ligands in regulating autoimmunity and infection. Nat Immunol.2007,8(3):239-45.

Tran E, Robbins PF, Rosenberg SA.'Final common pathway' of human cancer immunotherapy: targeting random somatic mutations. Nat Immunol.201718(3):255-262.

Wang X, Teng F, Kong L, Yu J. PD-L1 expression in human cancers and its association with clinical outcomes. Onco Targets Ther.2016;9:5023-39.

Wolchok JD, Hoos A, O'Day S, Weber JS, et al. Guidelines for the evaluation of immune therapy activity in solid tumors: immune-related response criteria. Clin Cancer Res.2009;15:7412-20.

Zhang L， Huang Y， Hong S， et al. Gemcitabine plus cisplatin versus fluorouracil plus cisplatin in recurrent or metastatic nasopharyngeal carcinoma: a multicentre，randomised，open-label，phase 3 trial. Lancet 2016;388:1883-92.

17. PROTOCOL CHANGES

The major changes from the previous protocol are summarized as follows:

1. The PD-L1 positive analysis set will be removed from the study objectives and endpoints, and ORR in the planned FAS population and PD-L1 positive population will not be used as a co-primary endpoint in this study.

2. To further clarify that the primary endpoint is ORR assessed by IRRC per RECIST v1.1

3. Clarify PD-L1 cut-off value and definition of PD-L1 positive/negative subgroup

4. Redefine subject discontinuation criteria

5. Modify the planned enrollment sample size and adjust the corresponding statistical analysis plan, including the interim analysis

6. To further clarify whether disease progression is required for reporting as SAEs and the reporting requirements for AESIs

7. Updated SAE reporting process instructions in accordance with the requirements of Good Clinical Practice (Version 2020)

18. APPENDICES

Appendix 1: Response Evaluation Criteria in Solid Tumors Version 1.1 and Immune-related Response Evaluation Criteria

1. Measurability of Tumor at Baseline

1.1. Definition

At baseline, tumor lesions/lymph nodes will be categorized measurable or non-measurable as follows:

1.1.1. Measurable

- Tumor lesions: Must be accurately measured in at least one dimension (longest diameter in the plane of measurement is to be recorded) with a minimum size of:

  – 10 mm by CT scan (CT scan slice thickness no greater than 5 mm).

  – 10 mm caliper measurement by clinical exam (lesions which cannot be accurately measured with calipers should be recorded as non-measurable).

  – 20 mm by chest X-ray.

Measurable malignant lymph nodes: To be considered pathologically enlarged and measurable, a lymph node must be ≥ 15 mm in short diameter when assessed by CT scan (CT scan slice thickness recommended to be no greater than 5 mm). At baseline (ie, screening phase) and in follow-up (ie, all measurements after screening), only the short axis will be measured and followed. See notes below on 'Baseline documentation of target and non-target lesions' for information on lymph node measurement.

1.1.2. Non-measurable

All other lesions, including small lesions (longest diameter <10 mm or pathological lymph nodes with ≥10 to <15 mm short axis) as well as truly non-measurable diseases. Lesions considered truly non-measurable include: leptomeningeal disease, ascites, pleural or pericardial effusion, inflammatory breast disease, lymphangitic involvement of skin or lung, abdominal masses/abdominal organomegaly identified by physical examination that is not measurable by reproducible imaging techniques.

1.1.3. Special Considerations Regarding Measurability of Lesions

Bone lesions, cystic lesions, and lesions previously treated with local therapy require particular comments:

1.1.4. Bone Lesions

- Bone scan, positron emission tomogram (PET) scan or plain films are not considered adequate imaging techniques to measure bone lesions. However, these techniques can be used to confirm the presence or disappearance of bone lesions.

- Lytic bone lesions or mixed lytic-blastic lesions, with identifiable soft tissue components, that can be evaluated by cross sectional imaging techniques (such as CT or MRI) can be considered as measurable lesions if the soft tissue component meets the definition of measurability described above.
- Blastic bone lesions are non-measurable.

1.1.5. Cystic Lesions

- Lesions that meet the criteria for radiographically defined simple cysts should not be considered as malignant lesions (neither measurable nor non-measurable) since they are, by definition, simple cysts.
- "Cystic lesions" thought to represent cystic metastases can be considered as measurable lesions, if they meet the definition of measurability described above. However, if non-cystic lesions are present in the same patient, these are preferred for selection as target lesions.

1.1.6. Lesions with Prior Local Treatment

- Tumor lesions located in previously irradiated areas or an area subjected to other loco-regional therapy cannot be considered measurable unless there has been demonstrated progression in the lesion.

1.2. Specification for Measuring Methods

1.2.1. Measurement of Lesions

All measurements should be recorded in metric notation using calipers if clinically assessed. All baseline evaluations should be performed as close as possible to the treatment start and never more than 4 weeks before the beginning of the treatment.

1.2.2. Evaluation Methods

The same method of assessment and the same technique should be used to characterize each identified and reported lesion at baseline and during follow-up. Imaging based evaluation should always be done rather than clinical examination unless the lesions being followed cannot be imaged but are assessable by clinical examination.

CT, MRI: CT is the best currently available and reproducible method to measure lesions selected for response assessment. This guideline has defined measurability of lesions on CT scan based on the assumption that CT slice thickness is 5 mm or less. When CT scans have slice thickness greater than 5 mm, the minimum size for a measurable lesion should be twice the slice thickness. MRI is also acceptable in certain situations (eg, for body scans).

2. Tumor Response Evaluation

2.1. Assessment of Overall Tumor Burden and Measurable Disease

To assess objective response or future progression, it is necessary to estimate the overall

tumor burden at baseline and use this as a comparator for subsequent measurements. In this study, only patients with measurable disease at baseline should be included. (See definition "1.1.2")

2.2. Baseline Documentation of 'Target' and 'Non-target' Lesions

When more than one measurable lesion is present at baseline, all lesions up to a maximum of five lesions totally representing all involved organs should be identified as target lesions and will be recorded and measured at baseline (this means in instances where subjects have only one or two organ sites involved, a maximum of two and four lesions will be recorded, respectively).

Target lesions should not be selected on the basis of their size (lesions with the longest diameter), and should be representative of all involved organs, but in addition should be those that lend themselves to reproducible repeated measurements. It may be the case that, on occasion, the largest lesion does not lend itself to reproducible measurement in which circumstance the next largest lesion with reproducible measurement should be selected.

Lymph nodes merit special mention since they are normal anatomical structures which may be visible by imaging even if not involved by tumor. As noted above, pathological nodes which are defined as measurable and may be identified as target lesions must meet the criterion of a short axis of ≥15 mm by CT scan. Only the short axis of these nodes will contribute to the baseline sum. The short axis of the node is the diameter normally used by radiologists to judge if a node is involved by solid tumor. Node size is normally reported as two dimensions in the plane in which the image is obtained (for CT scan, this is almost always the axial plane; for MRI, the plane of acquisition may be axial, saggital or coronal). The smaller of these measures is the short axis. For example, an abdominal node which is reported as being 20 mm × 30 mm has a short axis of 20 mm qualifies as a malignant, measurable node. In this example, 20 mm should be recorded as the node measurement. All other pathological nodes (those with short axis ≥10 mm but <15 mm) should be considered non-target lesions. Nodes that have a short axis <10 mm are considered non-pathological and should not be recorded or followed.

A sum of the diameters (longest for non-node lesions, short axis for node lesions) for all target lesions will be calculated and reported as the baseline sum diameters. If lymph nodes are to be included in the sum, then as noted above, only the short axis is added into the sum. The baseline sum diameters will be used as reference to further characterize any objective tumor regression in the measurable dimension of the disease.

All other lesions (or sites of disease) including pathological lymph nodes should be identified as non-target lesions and should also be recorded at baseline. Measurements are not required and these lesions should be followed as "present", "absent", or in rare cases "definite progression". In addition, multiple non-target lesions involving the same organ may be recorded as a single item on the CRF, such as 'multiple enlarged pelvic lymph nodes' or 'multiple liver metastases'.

2.3. Response Criteria (RECIST v1.1)

This section provides the definitions of the objective tumor response criteria used to determine target, non-target and new lesions based on RECIST v1.1.

2.3.1. Evaluation of Target Lesions

Complete Response (CR): Disappearance of all target lesions. Any pathological lymph nodes (whether target or non-target lesions) must have decreased in size to have a short axis of <10 mm.

Partial Response (PR): At least a 30% decrease in the sum of diameters of target lesions, taking as reference the baseline sum diameters.

Progressive Disease (PD): At least a 20% increase in the sum of diameters of target lesions, taking as a reference the smallest sum on study (this includes the baseline sum if that is the smallest on study). In addition to the relative increase of 20%, the sum must also demonstrate an absolute increase of at least 5 mm. (Note: the appearance of one or more new lesions is also considered progression.)

Stable Disease (SD): Neither sufficient shrinkage to judge as PR nor sufficient increase to judge as PD, taking as reference the smallest sum diameters while on study.

2.3.2. Special Notes on the Assessment of Target Lesions

Lymph nodes: Lymph nodes identified as target lesions should always have the actual short axis measurement recorded (measured in the same anatomical plane as the baseline examination), even if the nodes regress to below 10 mm on study. This means that when lymph nodes are included as target lesions, the "sum" of lesions may not be zero even if complete response criteria are met, since a normal lymph node is defined as having a short axis of <10 mm. For PR, SD and PD, the actual short axis measurement of the lymph nodes is to be included in the sum of target lesions.

Target lesions that become "too small to measure": While on study, all lesions (nodal and non-nodal) recorded at baseline should have their actual measurements recorded at each subsequent evaluation, even if the lesions are very small (e.g., 2 mm). However, sometimes lesions or lymph nodes which are recorded as target lesions at baseline become so faint on CT scan that the radiologist considers it inappropriate to provide an exact measurement and may report them as being "too small to measure". When this occurs, a value needs to be recorded on the CRF. If it is the opinion of the radiologist that the lesion has likely disappeared, the measurement should be recorded as 0 mm. If the lesion is believed to be present and is faintly seen but too small to measure, a default value of 5 mm should be assigned. (Note: It is less likely that this rule will be used for lymph nodes since they usually have a definable size when normal and are frequently surrounded by fat such as in the retroperitoneum; however, if a lymph node is believed to be present and is faintly seen but too small to measure, a default value of 5 mm should be assigned in this circumstance as well). This default value is derived from the 5 mm CT slice thickness (but should not be changed with varying CT slice thickness). The measurement of these lesions is potentially non-reproducible, therefore

providing this default value will prevent misjudgement of disease remission or progression due to measurement error. To reiterate, however, if the radiologist is able to provide an actual measure, that should be recorded, even if it is below 5 mm.

Lesions that split or coalesce on treatment: When non-nodal lesions “fragment”, the longest diameters of the fragmented portions should be added together to calculate the target lesion sum. Similarly, as lesions coalesce, a plane between them may be maintained that would aid in obtaining maximal diameter measurements of each individual lesion. If the lesions have truly coalesced such that they are no longer separable, the vector of the longest diameter in this instance should be the maximal longest diameter for the “coalesced lesion”.

2.3.3. Non-Target Evaluation

This section provides the definitions of the criteria used to determine the tumor response for the group of non-target lesions. While some non-target lesions may actually be measurable, they need not be measured and instead should be assessed only qualitatively at the time points specified in the protocol.

CR: Disappearance of all non-target lesions and normalization of tumor marker level. All lymph nodes must be non-pathological in size (<10 mm short axis).

Non-CR/Non-PD: Persistence of one or more non-target lesions and/or maintenance of tumor marker level above the normal limits.

PD: Definite progression of existing non-target lesions. (see comments below; note: the appearance of one or more new lesions is also considered progression.)

2.3.4. Special Notes on Assessment of Progression of Non-target Disease

If the subject has additional measurable diseases, the concept of progression of non-target diseases requires additional explanation as follows. In this case, to achieve “definite progression” on the basis of the non-target disease, there must be an overall level of substantial worsening in non-target disease such that, even in presence of SD or PR in target disease, and the overall tumor burden has increased sufficiently to discontinue treatment. A modest “increase” in the size of one or more non-target lesions is usually not sufficient to qualify for definite progression status. Therefore, when target disease achieves SD or PR, the determination of overall progression only on the basis of changes in non-target disease will be extremely rare.

2.3.5. New Lesions

The appearance of new malignant lesions denotes disease progression; therefore, some comments on the detection of new lesions are important. There are no specific criteria for the identification of new radiographic lesions; however, the finding of a new lesion should be definite: i.e. not attributable to differences in scanning technique, change in imaging modality or findings thought to represent something other than tumor (for example, some “new” bone lesions may be simply treated or worsening of pre-existing lesions). This is particularly

important when the patient's baseline lesions show PR or CR. For example, necrosis of a liver lesion may be reported on a CT scan report as a "new" cystic lesion, which it is not.

A lesion identified on a follow-up study in an anatomical location that was not scanned at baseline is considered a new lesion and will indicate disease progression. For example, a subject with visceral disease at baseline has a brain CT or MRI during the study which shows metastatic tumor. The brain metastasis of the patients is considered to be evidence of PD even if he/she did not have brain imaging at baseline.

If a new lesion is equivocal, for example because of its small size, continued therapy and follow-up evaluation will clarify if it represents truly new disease. If repeat scans confirm there is definitely a new lesion, then progression should be declared using the date of the initial scan.

2.4. Evaluation of Overall Response

The patient's overall response assignment will depend on the findings of both target and non-target disease and will also take into consideration the appearance of new lesions. It is assumed that at time point specified in each protocol, a response assessment occurs. The following table provides a summary of the overall response status calculation at each time point for patients who have measurable disease at baseline.

Table 14 Overall response

**Time point response: patients with target (+/- non-target) disease**			
**Target lesions**	**Non-target lesions**	**New Lesions**	**Overall response**
CR	CR	None	CR
CR	Non-CR/Non-PD	None	PR
CR	Not evaluated	None	PR
PR	Non-PD or not all evaluated	None	PR
SD	Non-PD or not all evaluated	None	SD
Not all evaluated	Non-PD	None	NE
PD	Any	Yes or No	PD
Any	PD	Yes or No	PD
Any	Any	Yes	PD

Abbreviations: CR = complete response; PR = partial response; SD = stable disease; PD = progressive disease; NE = not evaluable.

2.4.1. Missing Assessment and Inevaluable Designation

When no imaging/measurement is done at all at a particular time point, the subject is not evaluable at that time point. If only a subset of lesion measurements are made at an assessment, usually the case is also considered not evaluable at that time point, unless a convincing argument can be made that the contribution of the individual missing lesion would not change the assigned time point response. This would be most likely to happen in the case of PD. For example, if a patient has a baseline sum of 50 mm with 3 measured lesions and at

follow-up only 2 lesions are assessed, but those give a sum of 80 mm, the patient will have achieved PD status, regardless of the contribution of the missing lesion.

2.4.2. Special Notes on Response Assessment

When nodal disease is included in the sum of target lesions and the nodes decrease to “normal” size (<10 mm), they may still have a measurement reported on scans. This measurement should be recorded even though the nodes are normal in order to avoid overstating disease progression based on increase in size of the nodes. As noted earlier, this means that patients with CR may not have a total sum of “0” on the CRF.

Patients with a global deterioration of health status requiring discontinuation of treatment without objective evidence of disease progression at that time should be reported as “symptomatic deterioration”. Every effort should be made to document objective progression even after discontinuation of treatment. Symptomatic deterioration is not a descriptor of an objective response: it is a reason for stopping study therapy. The objective response status of such subjects is to be determined by evaluation of target and non-target disease.

For equivocal findings of progression (e.g. very small and uncertain new lesions; cystic changes or necrosis in existing lesions), treatment may continue until the next scheduled assessment. If at the next scheduled assessment, progression is confirmed, the date of progression should be the earlier date when progression was suspected.

Appendix 2: Immune-related Response Evaluation Criteria in Solid Tumors (irRECIST)

The irRECIST (**Nishino M, 2013**) is an integration of RECIST guideline v1.1 and the initial irRC (**Wolchok JD, 2009**). The latter criterion incorporates important principles used for the assessment of cancer immunotherapy, but is intrinsically different from RECIST in the use of two-dimensional tumor assessments. In contrast, irRECIST is based on one-dimensional tumor measurements. Response assessments should be performed on the basis of irRECIST at each time point based on the following:

- Immune-related complete response (irCR): Complete disappearance of all tumor lesions (target and non-target lesions) with no new measurable or non-measurable diseases for at least 4 weeks from the date of documentation of irCR. All lymph nodes must have a short axis < 10 mm.
- Immune-related partial response (irPR): The sum of diameters of all target lesions is measured and collected as the baseline sum diameters. At each subsequent tumor assessment, the sum of diameters of all target lesions is added to the sum of diameters of new measurable diseases as the overall tumor burden. In the absence of irCR, a decrease of ≥ 30% in overall tumor burden relative to baseline is considered an irPR. It must be determined within no less than 4 weeks after the first irPR assessment.
- Immune-related progressive disease (irPD): At least a 20% increase in tumor burden (ie, sum of diameters of target lesions and any new measurable diseases), taking as an reference the smallest sum on study (this includes the baseline sum if that is the smallest on study). In addition to the relative increase of 20%, the sum must also demonstrate an absolute increase of at least 5 mm. Tumor assessments using immune-related criteria for progressive disease include the contribution of new measurable disease. Each net percentage change in tumor burden per assessment takes into account the size and growth kinetics of new and old lesions at their appearance. Assuming no clinical deterioration, progressive disease will need to be determined by continuous scans (with an interval of at least 4 weeks).
- Immune-related stable disease (irSD): Neither sufficient shrinkage to judge as irPR nor sufficient increase to judge as irPD, taking as reference the smallest sum diameters while on study.

Appendix 3: Listing of Autoimmune Diseases Present Prior to Inclusion

Subjects will be carefully asked if they have any acquired or innate immunodeficiency or autoimmune diseases, and such subjects should be excluded from the study. Unless the likelihood of the subject's history of previous allergic disease and childhood arthralgia being suspected of autoimmune disease is very low. In addition, transient autoimmune manifestations (eg, Lyme arthritis) due to acute infectious disease may be included if cured with antibiotics. Contact the medical monitor if an unconfirmed autoimmune disease requires exclusion.

Diseases associated with autoimmunity, including but not limited to:

Acute sporadic encephalomyelitis
IgA nephropathy
Addison's disease
Inflammatory bowel disease
Alopecia universalis
Interstitial cystitis
Ankylosing spondylitis
Myasthenia gravis syndrome
Antiphospholipid antibody syndrome
Lupus erythematosus
Aregenerative anemia
Lyme disease-chronic
Asthma
Meniere's syndrome
Autoimmune haemolytic anaemia
Corneal ulcer
Autoimmune hepatitis
Localized autoimmune hypophysitis
Multiple sclerosis
Autoimmune hypoparathyroidism
Myasthenia gravis
Autoimmune myocarditis
Neuromyotonia
Autoimmune oophoritis
Myoclonic syndrome
Autoimmune orchitis
Optic neuritis
Autoimmune thrombocytopenic purpura
Ords thyroiditis
Behcet's disease
Pemphigus
Bullous pemphigoid
Pernicious anaemia
Gluten allergy
Multiple arteritis
Chronic fatigue syndrome
Polyarthritis
Chronic inflammatory demyelination
Polyneuropathy
Autoimmune syndrome
Churg-Strauss syndrome
Primary biliary cirrhosis
Crohn's disease
Psoriasis/dermatomyositis
Reiter's syndrome
Type I diabetes mellitus
Rheumatoid arthritis
Autonomic dysfunction
Sarcoidosis
Eczema
Scleroderma
Sjogren's syndrome
Epidermolysis bullosa
Stiff-man syndrome
Pemphigoid gestationis
Polyarteritis
Giant cell arteritis
Ulcerative colitis
Goodpasture's syndrome
Vitiligo
Graves' disease
Vogt-Kovanagi-Harada disease
Guillain-Barré syndrome
Vulval pain
Hashimoto's disease
Wegener's granulomatosis
Kawasaki's disease

Appendix 4: New York Heart Association Classification Criteria for Cardiac Function

Categorical	Symptoms
I	No limitation in physical activity. Ordinary physical activity does not cause undue fatigue, palpitation, or dyspnea (shortness of breath).
II	Limitation of physical activity. Comfortable at rest, but ordinary physical activity causes fatigue, palpitation, dyspnea (shortness of breath).
III	Marked limitation of physical activity. Comfortable at rest, but less than ordinary activity causes fatigue, palpitation, or dyspnea.
IV	Any physical activity causes discomfort. Symptoms of heart failure at rest. If any physical activity is undertaken, discomfort increases.

Adapted from

Dolgin M, Association NYH, Fox AC, Gorlin R, Levin RI, New York Heart Association. CriteriaCommittee.Nomenclature and criteria for diagnosis of diseases of the heart and great vessels.9th ed. Boston, MA:LippincottWilliams and Wilkins; March 1,1994.

Original Source:Criteria Committee, New York Heart Association, Inc. Diseases of the Heart andBloodVessels. Nomenclature and Criteria for diagnosis,6th edition Boston, Little, Brown and Co.1964, p114.

Appendix 5: Guidelines for Diagnostic Assessment of Immune-related Adverse Events

The recommendations below for the diagnosis of any irAE are intended as a guidance. This document should be used in conjunction with expert clinical judgment (by specialist physicians experienced in the treatment of cancer using immunological agents), and individual institutional guidelines or policies.

Criteria used to diagnose irAEs include blood tests, diagnostic imaging, histopathology, and microbiology assessments to exclude alternative causes such as infection, disease progression, and adverse effects of concomitant drugs. In addition to the results of these tests, the following factors should be considered when making an irAE diagnosis:

- What was the temporal relationship between initiation of anti-PD-1 and CTLA-4 antibodies and the adverse event?
- How did the patient respond after discontinuation of anti-PD-1 and CTLA-4 antibodies?
- Did the event recur after resumption of treatment with anti-PD-1 and CTLA-4 antibodies?
- Was there a clinical response to corticosteroids?
- Is the event an autoimmune endocrinopathy?
- Is PD or an alternative diagnosis a more likely explanation?

Immune-related Toxicity	Diagnostic Evaluation Guideline
Thyroid disorder	Scheduled and repeat thyroid function tests (TSH and T4).
Hypophysitis	Check visual fields and consider pituitary endocrine axis blood profile. Perform pituitary and whole brain MRI in patients with headache, visual disturbance, unexplained fatigue, asthenia, weight loss and unexplained constitutional symptoms. Consider consultation with an endocrinologist if an abnormality is detected.
Noninfectious pneumonitis	All patients presenting with new or worsened pulmonary symptoms or signs, such as an upper respiratory infection, new cough, shortness of breath or hypoxia should be assessed by high-resolution CT. Consider pulmonary function test including DLCO. Radiographic appearance is often nonspecific. Depending on the location of the abnormality, bronchoscopy and bronchoalveolar lavage or lung biopsy may be considered. Consult with a respiratory medicine physician for cases of uncertain cause.
Neurotoxicity	Perform a comprehensive neurological examination and brain MRI for all CNS symptoms; review alcohol history and other medications. Conduct a diabetic screen, and assess blood B12/folate, HIV status, TFTs, and consider autoimmune serology. Consider the need for brain/spine MRI/MRA and

Immune-related Toxicity	Diagnostic Evaluation Guideline
	nerve conduction study for peripheral neuropathy. Consult with a neurologist if there are abnormal findings.
Colitis	Review dietary intake and exclude steatorrhea. Consider comprehensive testing, including the following: FBC, UEC, LFTs, CRP, TFTs, stool microscopy and culture, viral PCR, Clostridium difficile toxin, cryptosporidia (drug-resistant organism). In case of abdominal discomfort, consider imaging, eg, X-ray, CT scan. If a patient experiences bleeding, pain or distension, consider colonoscopy with biopsy and surgical intervention, as appropriate.
Eye Disorders	If patients experience eye inflammation, blurred vision, or other visual disturbances, refer the patient urgently to an ophthalmologist for evaluation and management.
Hepatitis	Check ALT/AST/total bilirubin, INR/albumin; the frequency will depend on the severity of the AE (eg, daily if Grade 3-4; every 2-3 days if Grade 2, until recovering). Review medications (eg, statins, antibiotics) and alcohol history. Perform liver screen including hepatitis A/B serology, hepatitis E PCR and assess anti-ANA/SMA/LKM/SLA/LP/LCI, and iron metabolism studies. Consider imaging, eg, ultrasound scan for metastases or thromboembolism. Consult with a hepatologist and consider liver biopsy.
Renal Toxicity	Review dehydration status, water-sodium retention and medication history. Routine Urinalysis and urine culture. Consider renal ultrasound scan, protein assessment (dipstick/24-hour urine collection), or phase-contrast microscopy. Refer to nephrology for further management assistance.
Dermatology	Consider other causes by conducting a physical examination; consider dermatology referral for skin biopsy.
Rheumatology	Conduct musculoskeletal history and perform complete musculoskeletal examination. Consider joint X-ray and other imaging as required to exclude metastatic disease. Perform autoimmune serology and refer to rheumatology for further management assistance.

Abbreviations: AE = adverse event; ALT = alanine aminotransferase; ANA = antinuclear antibody; AST = aspartate aminotransferase; CNS = central nervous system; CRP = C-reactive protein; CT = computed tomography; DLCO = diffusing capacity for carbon monoxide; FBC = full blood count; HIV = human immunodeficiency virus; INR = international normalized ratio; LCI = liver cystolic antigen; LFT = liver function test; LKM = liver kidney microsomal antibody; LP = liver pancreas antigen; MRA = magnetic resonance angiogram; MRI = magnetic resonance imaging; PCR = polymerase chain reaction; SLA = soluble liver antigen; SMA = smooth muscle antibody; T4 = thyroxine; TFT = thyroid function tests; TSH = thyroid stimulating hormone; UEC = urea electrolytes and creatinine.

Signature Page of Sponsor

Sponsor's Approval

A Single-arm, Open-label, Multicenter Phase II Clinical Study of AK105 in Patients with Metastatic Nasopharyngeal Carcinoma after Failure of Second and Subsequent Lines of Chemotherapy

This clinical study protocol has been reviewed and approved by the following representative of Akeso Tiancheng (Guangdong) Co., Ltd.

______________________	______________________
**Print Name**	**Signature**
	MM DD YYYY
______________________	______________________
**Title**	**Date (MM/DD/YYYY)**

Investigator's Signature Page

A Single-arm, Open-label, Multicenter Phase II Clinical Study of AK105 in Patients with Metastatic Nasopharyngeal Carcinoma after Failure of Second and Subsequent Lines of Chemotherapy

I have reviewed the protocol and agree to comply with the ethical principles derived from the Declaration of Helsinki and International Council for Harmonization guidelines on GCP, and the requirements of any regulatory authority and/or Institutional Review Board/Independent Ethics Committee (IRB/IEC) in conducting the protocol.

I understand that modifications to the protocol will not be made without obtaining the written approval from the sponsor. All protocol changes must be submitted to the applicable regulatory authority and the IRB/IEC and be approved by the IRB/IEC before implementation unless it is necessary to eliminate direct injury to the subject or, in the opinion of the sponsor, the changes involve only logistics or administration.

I agree to open my subject study records to representatives of the sponsor and relevant regulatory authorities so that they can verify the data I have entered into the CRF. I understand my responsibilities as the sponsor-designated principal investigator.

I understand that the sponsor may decide to suspend or prematurely terminate the study at any time for any reason; and such decision will be communicated to me in writing. Conversely, if I decide to withdraw from the study, I shall immediately inform the sponsor in writing.

__________________________ __________________________

Print Name **Signature**

MM DD YYYY

__________________________ __________________________

Title **Date (MM/DD/YYYY)**
